# Wild-Type TP53 Predicts Poor Prognosis in Patients with Gastric Cancer

**DOI:** 10.26502/jcsct.50790107

**Published:** 2021-03-18

**Authors:** Wenhong Deng, Qiongyu Hao, Jaydutt Vadgama, Yong Wu

**Affiliations:** 1 Department of General Surgery, Renmin Hospital of Wuhan University, Wuhan, China; 2 Division of Cancer Research and Training, Department of Internal Medicine, Charles Drew University of Medicine and Science, CA, USA

**Keywords:** TP53, Gastric cancer, Prognosis, Mutation, Survival rate

## Abstract

TP53 gene is often mutated in gastric cancer (GC), nonetheless its relationship with clinicopathological characteristics and prognosis is still unclear. Here, we sought to ascertain the difference in clinical phenotypes between TP53 wild-type and mutant tumors in confirmed gastric cancer patients. To this end, we analyzed TP53 mutation status of 415 TCGA GC patients in relation to their clinical and pathological features as well as prognosis. Longrank Test showed that the survival rate of gastric cancer patients with TP53 WT was significantly lower than that of TP53 mut. Compared with TP53 mut gastric cancer patients with low mRNA expression, TP53 WT patients with low mRNA expression have lower overall survival rate. The death risk of TP53 WT gastric cancer patients is 1.395 times that of TP53 mut gastric cancer patients. The death risk of TP53 mut gastric cancer patients is not related to age, and advanced age is not a risk factor. However, the death risk of TP53 WT patients with gastric cancer increases with age, and the death risk of patients over 70 years old is 1.899 times that of patients under 60 years old. These results suggest that the prognosis of elderly gastric cancer patients with TP53 WT is worse.

**Conclusion:**

our results indicate that the status of TP53 mutation in GC is significantly correlated with clinical or molecular categories and that the prognosis of GC patients with WT TP53 is worse than that of patients with mutant TP53. Therefore, our data emphasize the importance of distinguishing TP53 WT to predict poor overall survival and relapse-free survival in patients with GC.

## 1. Introduction

Gastric cancer (GC) is one of the most prevalent cancers in the world, responsible for around 70,000 new cases and 650,000 deaths annually [1, 2]. Although diagnostic techniques have improved, many patients still suffer from advanced diseases at the time of diagnosis, and the treatment results of such patients are poor [3]. In addition, the clinical course and prognosis of GC are heterogeneous. Nonetheless, the basic mechanisms for addressing this issue have not been completely understood. With regard to molecular abnormalities in GC, some genetic alterations in gastric carcinogenesis have been clarified, including oncogenes ERBB2, KRAS, b-catenin, and PIK3CA [4–7] as well as tumor suppressor genes TP53, CDH1, p16, and ARID1A) [8–11]. TP53 is the most studied gene in various cancers and can be a potential GC biomarker candidate. TP53 protein has crucial cellular functions such as cell cycle control, apoptosis and DNA repair [12, 13]. Through advances in new technologies, for example whole exome sequencing, TP53 has been identified as the most commonly mutated gene in human cancers, with changes occurring in approximately 50% of human cancers [8–11]. Specifically, about half of reported cases of GC, the primary cause of cancer death in the Asia-Pacific region, show somatic mutations in TP53 [14]. Furthermore, the long-standing role of TP53 in the response to DNA damaging drugs in cancer chemotherapy [15] is an additional imperative aspect of treatment. Although mutations in TP53 are prevalent in cancer, a number of retrospective studies have yet to determine association between TP53 defects (i.e. mutations and amplification) and clinicopathological phenotypes [16], and lack of recognized clinical significance between prognosis and TP53 status has become one of the most contentious subjects in cancer research including GC [16–19]. It is believed that differences in reported correlations are mainly due to tumor heterogeneity, complexity of p53 pathways and clear different clinical stages [16]. Therefore, mutation status of TP53 is not only significant but also has important clinical significance for prognosis prediction of tumor patients, and hence further exploration should be performed accordingly.

Here we aimed to elucidate the relationship between clinicopathological characteristics and prognosis of GC and TP53 status by systematically exploring TP53 mutations in The Cancer Genome Atlas (TCGA) [10] GC patient datasets. The survival time and clinical characteristics of GC patients in different groups were compared at three levels (wild type and mutant TP53 gene, high and low expression of TP53 mRNA, and high and low expression of TP53 protein). Moreover, we conducted a cross-sectional analysis on whether the TP53 gene was mutated or not and the various levels of TP53 mRNA/protein expression to see the differences among patients from different subgroups. The highlight of this study is that the survival rate of TP53 WT GC patients is significantly lower than that of TP53 mut GC patients, suggesting that clinically, patients with gastric cancer have worse prognosis if they do not have TP53 mutation and that wild type TP53 is an indicator of poor prognosis in GC patients.

## 2. Materials and Methods

### 2.1 Clinical cohorts

The genomic, clinical and protein expression data of 478 stomach adenocarcinoma patients were obtained from The Cancer Genome Atlas (TCGA)(www.cbioportal.org). The detail information will been found in Stomach Adenocarcinoma (TCGA, Firehose Legacy).

### 2.2 Study design

TP53 mRNA Expression Z-scores has been taken into consideration. In this study, 63 patients who has no TP53 mRNA expression data was excluded from 478 patients. Unfortunately, 23 patients who has TP53 mRNA expression data, but without TP53 protein expression data. Therefore, 415 patients were analyzed based on TP53 mRNA expression (See supplementary material Figure S1 for details), and 392 patients were analyzed based on TP53 protein expression. The specific grouping is as follows:

### 2.3 Immunohistochemistry

After being deparaffinized and pretreated in citrate buffer, sections were incubated with normal goat serum for 15 min at room temperature. Sections were applied with PBS overnight at 4°C, with mouse anti P53 monoclonal antibody (Thermo Scientific, MS-187), rabbit anti CCNE1 polyclonal antibody (Sigma-Aldrich, HPA018169), mouse anti CDKN2A monoclonal antibody (Santa Cruz Biotechnology, sc-56330). The secondary antibody, biotinylated anti-rabbit and mouse immunoglobulin, was applied for 15 min at room temperature and sections were rinsed in PBS. Peroxidase conjugated streptavidin was applied for 15 min, followed by Diaminobenzidine (DAB) substrate for 10 min and hematoxylin for5 min. Finally, sections were rinsed with water, dehydrated, cleared and mounted with permanent mounting medium. Immunohistochemical staining was analyzed using Image Pro-Plus (version 6.0; Media Cybernetics, Silver Spring). Briefly, the positive staining area was selected as the area of interest (AOI). The area sum and integrated optical density (IOD) of the AOI were selected as the measurement parameters. The target protein expression was analyzed by comparing the IOD (P53, CCNE1and CDKN2A) in different groups. Finally, statistical analysis of the mean expression index for each duplicate was performed.

### 2.4 Statistical analysis

Data analysis were performed using SPSS20.0(SPSS Inc. Chicago, IL). Survival description was illustrated by the Kaplan-Meier curves, with P value determined by Log-rank Test. Hazard’s ratio (HR) was determined through the univariate and multivariate COX regression. Kruskal Wallis Test was used to analyze measurement data and Chi-squared Test was use to analyze counting data.

## 3. Results

### 3.1 The survival rate was significantly lower in gastric cancer patients with TP53 wild type versus TP53 mut

**Figure 1:**
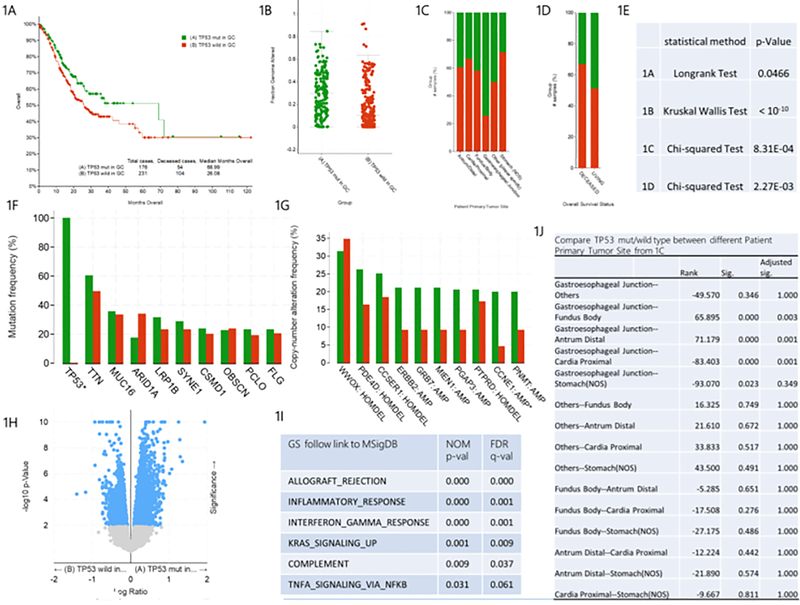
Clinical and genomic differences between TP53 mut and TP53 wild type gastric patients. A: Maplan-Meier curve of overall survival months (Longrank Test, P=0.0466); B: Fraction gene altered (Kruskal Wallis Test, P<10^−10^); C: Primary Tumor Site (Chi-squared Test, 8.31E-04); D: Overall Survival Status Site (Chi-squared Test, 2.27E-03); E: P-value of 1A-1D; F: Mutation Frequency(%); G: Copy-number Altered Frequency(%); H: Difference of RNA Seq RESM; I: Enrichment of specific and highly expressed gene pathways in TP53 wild gastric patients, compared with TP53 mut gastric patients; J: Pairwise comparison between different primany tumor sites in 1C. * means P<0.05.

As shown in Figure 1A, the median survival time of gastric cancer patients with TP53 mut was 68.99 months, and that of gastric cancer patients with TP53 wild type was 26.08 months. There was significant difference between the two groups by Longrank Test. We demonstrated that the survival rate of gastric cancer patients with TP53 wild type is significantly lower than that of gastric cancer patients with TP53 mut. In addition, we observed that the fraction gene altered of gastric cancer patients with TP53 mut was significantly higher than that of gastric cancer patients with TP53 wild type, suggesting that TP53 mutation caused more gene alteration (Figure 1B). The mutation rate of TP53 is different in different primary tumor sites of gastric cancer patients (Figure 1C and Figure 1J). The pairwise comparisons showed that the mutation rate of TP53 in gastroesophageal junction tumors is higher than that in fundus body, antrum distal and cardia proximal tumors. In Figure 1D, the analysis of overall survival status indicated that among the dead gastric cancer patients, the proportion of TP53 mut patients is smaller than that of TP53 wild type patients, while the proportion of TP53 mut patients is higher than that of TP53 wild type patients in the surviving gastric cancer patients.

Mutation Frequency analysis indicated that compared with TP53 wild type gastric cancer patients, TP53 mut gastric cancer patients had statistical difference only in TP53 gene mutation, while other gene mutations had no statistical difference. This further confirmed the key role of TP53 in the pathogenesis of gastric cancer (Figure 1F). Furthermore, Copy-number Altered Frequency analysis suggested that compared with TP53 wild type gastric cancer patients, only CCNE1 gene amplification increased significantly in TP53 mut gastric cancer patients and there is no statistical difference in the copy number of other genes. This suggests that TP53 mutation may increase the amplification copy of CCNE1 gene, which plays an important role in the pathogenesis of TP53 mutant gastric cancer (Figure 1G). RNA sequencing analysis (RNA Seq RESM) indicated that the expression and regulation range of TP53 downstream mRNA was significantly different between TP53 mut and TP53 wild type patients (Figure 1H). Compared with TP53 mut gastric cancer patients, the enrichment analysis of 1363 specific high-expression gene pathways (Table S1) in TP53 wild type gastric cancer patients showed that the following six pathways had obvious statistical differences, namely ALLOGRAFT_REJECTION, INFLAMMATORY_RESPONSE, INTERFERON_GAMMA_RESPONSE, KRAS_SIGNALING_UP, COMPLEMENT and TNFA_SIGNALING_VIA_NFKB (Figure 1I). We speculate that the six pathways may be related to the pathogenesis of TP53 wild type gastric cancer.

### 3.2 Comparison of clinical and genetic data of gastric cancer patients with high and low expression of TP53 mRNA

**Figure 2:**
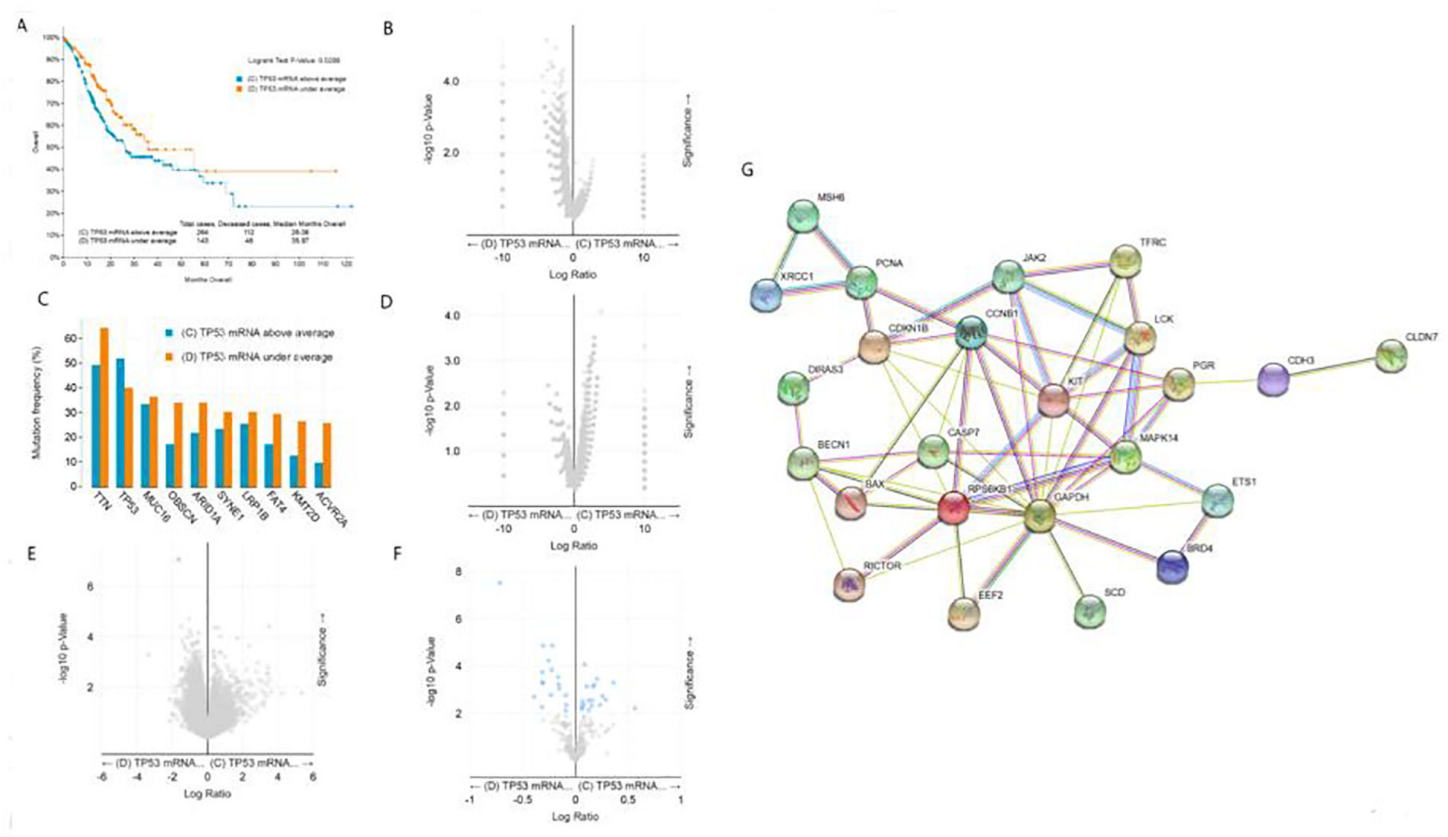
Clinical and genomic differences between gastric cancer patients with high (above average) and low (under average) expression of TP53 mRNA. A: Maplan-Meier curve of overall survival months (Longrank Test, P=0.0288); B: Gene mutation (no difference); C: Mutation Frequency(%) (no difference); D: Copy-number(no difference); E: mRNA change(only TP53 mRNA has difference); F: Protein expression difference; G: Protein-Protein Interaction Networks.

The median survival time of gastric cancer patients with high expression of TP53 mRNA was 26.08 months, while that of gastric cancer patients with low expression of TP53 mRNA was 35.97 months. Longrank Test showed that there was a significant statistical difference between them (P < 0.05). We reasoned that the survival rate of gastric cancer patients with low expression of TP53 mRNA was significantly higher than that of gastric cancer patients with high expression of TP53 mRNA (Figure 2A). Intriguingly, there was no significant difference in gene mutation including TP53 gene mutation between gastric cancer patients with low expression of TP53 mRNA and gastric cancer patients with high expression of TP53 mRNA (Figure 2B and 2C). In addition, there was also no significant difference in copy-number between the two groups (Figure 2D). As shown in Figure 2E, only the expression of TP53 mRNA was different between gastric cancer patients with low expression of TP53 mRNA and those with high expression of TP53 mRNA, but there was no difference in the expression of other mRNAs. Further analysis showed that the expression of TP53 protein was also different between the two groups (Figure 2F), and we speculate that the change of these proteins was caused by the difference of TP53 mRNA transcription. Figure 2G shows the protein interaction network related to TP53 mRNA transcription in gastric cancer patients. Taken together, the highlight of these results is that the survival rate of gastric cancer patients with low expression of TP53 mRNA is significantly higher than that of gastric cancer patients with high expression of TP53 mRNA. According to our analysis in Figure 1, i.e. the survival rate of gastric cancer patients with TP 53 mut is higher than that of gastric cancer patients with TP53 wild, we now ask what the relationship between TP53 gene mutation and mRNA expression is. We will conduct further stratified analysis.

### 3.3 Comparison of survival rate of patients with TP53 mutation accompanied with different mRNA expression

**Figure 3:**
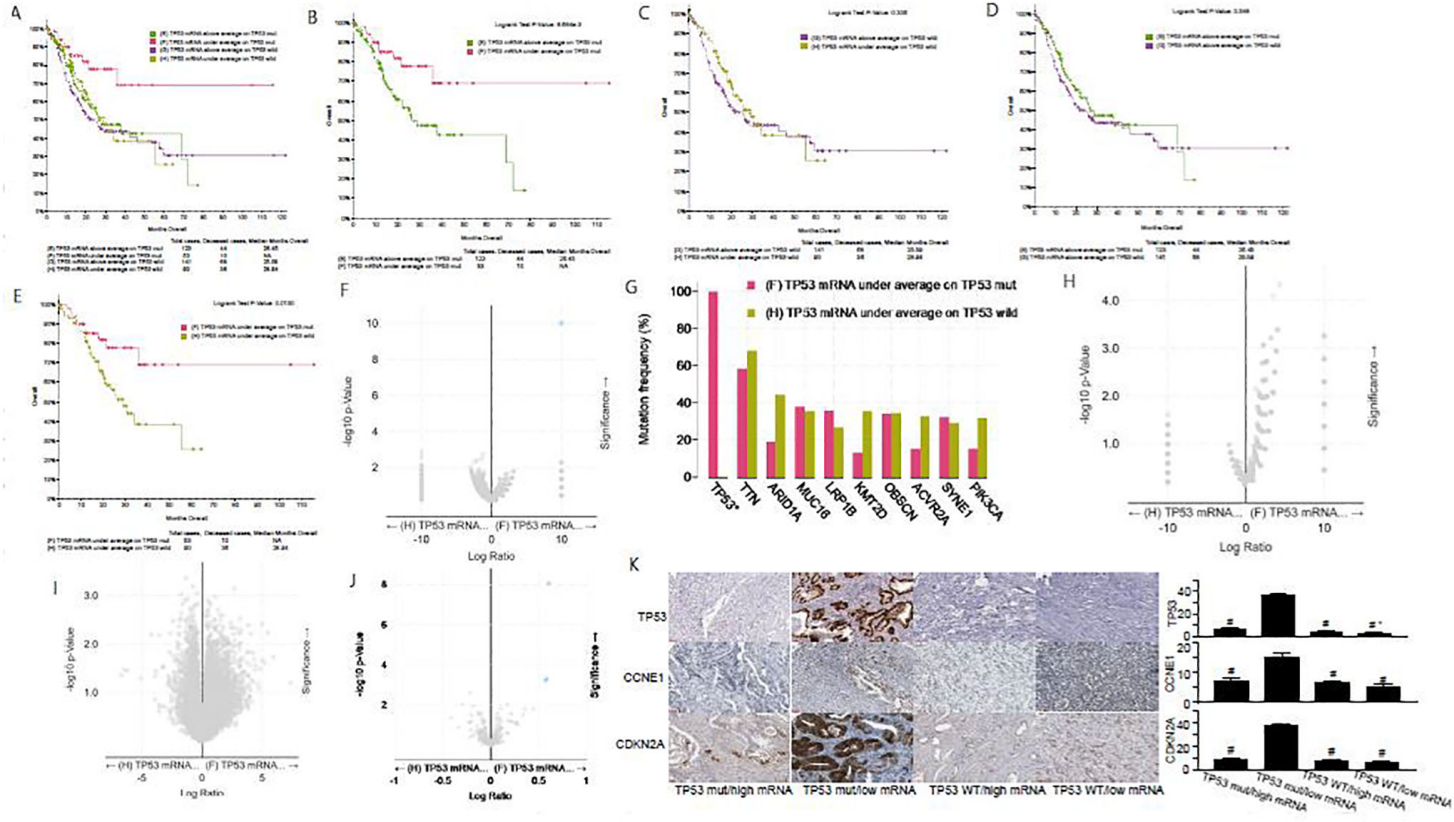
Clinical and genomic differences among 4 gastric cancer subgroups: TP53 mRNA high expression/TP53 mut, TP53 mRNA low expression/TP53 mut, TP53 mRNA high expression/TP53 WT, and TP53 mRNA low expression/TP53 WT. A: Maplan-Meier curve of overall survival months among 4 subgroups; B: Maplan-Meier curve between TP53 mRNA high expression(above average) in TP53 mut gastric patients and TP53 mRNA low expression(under average) in TP53 mut gastric patients (Longrank Test, P=8.684E-03); C: Maplan-Meier curve between TP53 mRNA high expression(above average) in TP53 wild gastric patients and TP53 mRNA low expression(under average) in TP53 wild gastric patients (Longrank Test, P=0.336); D: Maplan-Meier curve between TP53 mRNA high expression(above average) in TP53 mut gastric patients and TP53 mRNA high expression(above average) in TP53 wild gastric patients (Longrank Test, P=0.348); E: Maplan-Meier curve between TP53 mRNA low expression(under average) in TP53 mut gastric patients and TP53 mRNA low expression(under average) in TP53 wild gastric patients (Longrank Test, P=0.013); F: Mutations between TP53 mRNA low expression(under average) in TP53 mut gastric patients and TP53 mRNA low expression(under average) in TP53 wild gastric patients (only TP53 mutation has difference); G: Mutation Frequency(%) in 3F; H: Copy number change between TP53 mRNA low expression(under average) in TP53 mut gastric patients and TP53 mRNA low expression(under average) in TP53 wild gastric patients (no difference); I: mRNA change between TP53 mRNA low expression(under average) in TP53 mut gastric patients and TP53 mRNA low expression(under average) in TP53 wild gastric patients (no difference); J: protein expression between TP53 mRNA low expression(under average) in TP53 mut gastric patients and TP53 mRNA low expression(under average) in TP53 wild gastric patients; K: Typical expression of TP53, CCNE1 and CDKN2A protein in different subgroups. * means P<0.05.

The overall comparison among the four groups (TP53 mutation with high and low TP53 mRNA expression; TP53 wild type with high and low TP53 mRNA expression) shows that the 10-year survival rate of patients with TP53 mutation and low expression of TP53 mRNA is over 50% (Figure 3A), suggesting a good survival effect. In Figure 3B, we observed that among gastric cancer patients with TP53 mut, the survival rate of patients with low expression of TP53 mRNA was significantly higher than that of patients with high expression of TP53 mRNA. These results suggested that the increase of TP53 mRNA transcription in the presence of TP53 mut is an unfavorable factor for gastric cancer patients. In gastric cancer patients with TP53 wild type, the survival rate of patients with low expression of TP53 mRNA was not significantly different from that of patients with high expression of TP53 mRNA (Figure 3C). Moreover, in gastric cancer patients with high expression of TP53 mRNA, there was no significant difference in survival rate between TP53 mut and wild type patients (Figure 3D). In Figure 3E, we observed that the survival rate of TP53 mut patients with low expression of TP53 mRNA was significantly higher than that of TP53 wild type patients with low expression of TP53 mRNA.

The survival rate of TP53 wild type gastric cancer patients with low expression of TP53 mRNA was significantly higher than that of TP53 mut gastric cancer patients with low expression of TP53 mRNA (Figure 3F, 3G). However, there is no difference in gene mutation between the two groups except TP53, which also confirms that the difference in survival rate is only related to the mutation state of TP53. To sum up, the lower expression of TP53 mRNA in gastric cancer patients with TP53 wild type indicates worse prognosis. In addition, in gastric cancer patients with low expression of TP53 mRNA, the survival rate of TP53 wild type patients was significantly higher than that of mut patients. However, there is no difference in copy number and mRNA between the two groups, which further confirms that the difference in survival rate is only related to the mutation state of TP53 (Figure 3H and 3I). Of note, TP53 mut gastric cancer patients with low expression of TP53 mRNA have obvious high expression of TP53, CCNE1 and CDKN2A protein (Figure 3J), which were also confirmed by the immunohistochemistry analysis of tumor specimens from the corresponding clinical cases (Figure 3K). These results suggested that TP53 protein may play a role in the pathogenesis of gastric cancer by regulating CCNE1 and CDKN2A proteins. Taken together, we demonstrate that there is no significant difference in survival rate between TP53 mut gastric cancer patients with high expression of TP53 mRNA and TP53 wild type gastric cancer patients with high expression of TP53 mRNA and that the survival rate of TP53 mut gastric cancer patients with low TP53 mRNA expression is significantly higher than that of TP53 wild type gastric cancer patients with low TP53 mRNA expression.

### 3.4 Comparison of overall survival rate, mRNA and copy-number of gastric cancer patients with high expression and low expression of TP53 protein

**Figure 4:**
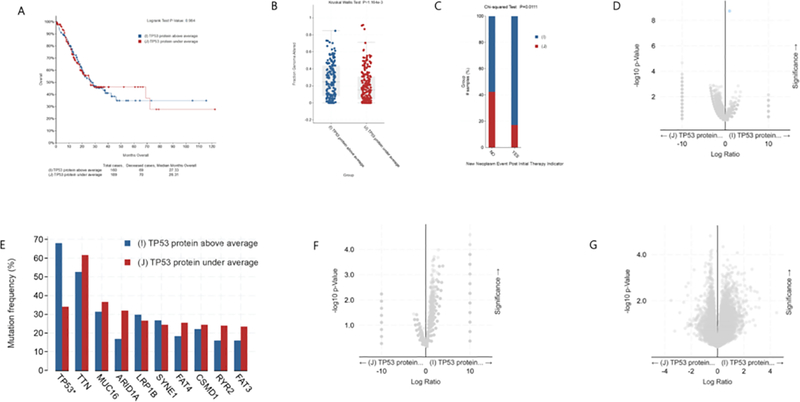
Clinical and genomic differences between gastric cancer patients with high (above average) and low (under average) expression of TP53 protein. A: Maplan-Meier curve of overall survival months (no difference); B: Fraction gene altered (Kruskal Wallis Test, P=1.164E-03); C: New neoplasm event post initial therapy indicator(Chi-squared Test, 0.0111); D: Gene mutation (only TP53 mutation has difference); E: Mutation Frequency(%) (only TP53 muation has difference); F: Copy-number(no difference); G: mRNA change(no difference).

As shown in Figure 4A, there was no significant difference in survival rate between gastric cancer patients with high expression and low expression of TP53 protein. The median survival time was 27.33 month (high expression) and 26.31month (low expression), respectively. However, the clinical feature Fraction Genome Altered of gastric cancer patients with high expression of TP53 protein are more changed than those with low expression of TP53 (Figure 4B). Intriguingly, after the initial treatment, patients with low expression of TP53 protein have lower probability of subsequent tumor recurrence than patients with high expression of TP53 protein (Figure 4C). There is no difference in gene mutation between gastric cancer patients with high expression of TP53 protein and those with low expression of TP53 protein except TP53 gene (Figure 4D, 4E). In addition, there is also no difference in mRNA and copy number between gastric cancer patients with high expression of TP53 protein and those with low expression of TP53 protein (Figure 4F and 4G). Together, our analysis shows that there is no difference in the overall survival rate, mRNA, copy-number, etc. between gastric cancer patients with high expression and low expression of TP53 protein.

### 3.5 Comparison of survival rate of patients with TP53 mutation and wild type accompanied with different TP53 protein expression

**Figure 5:**
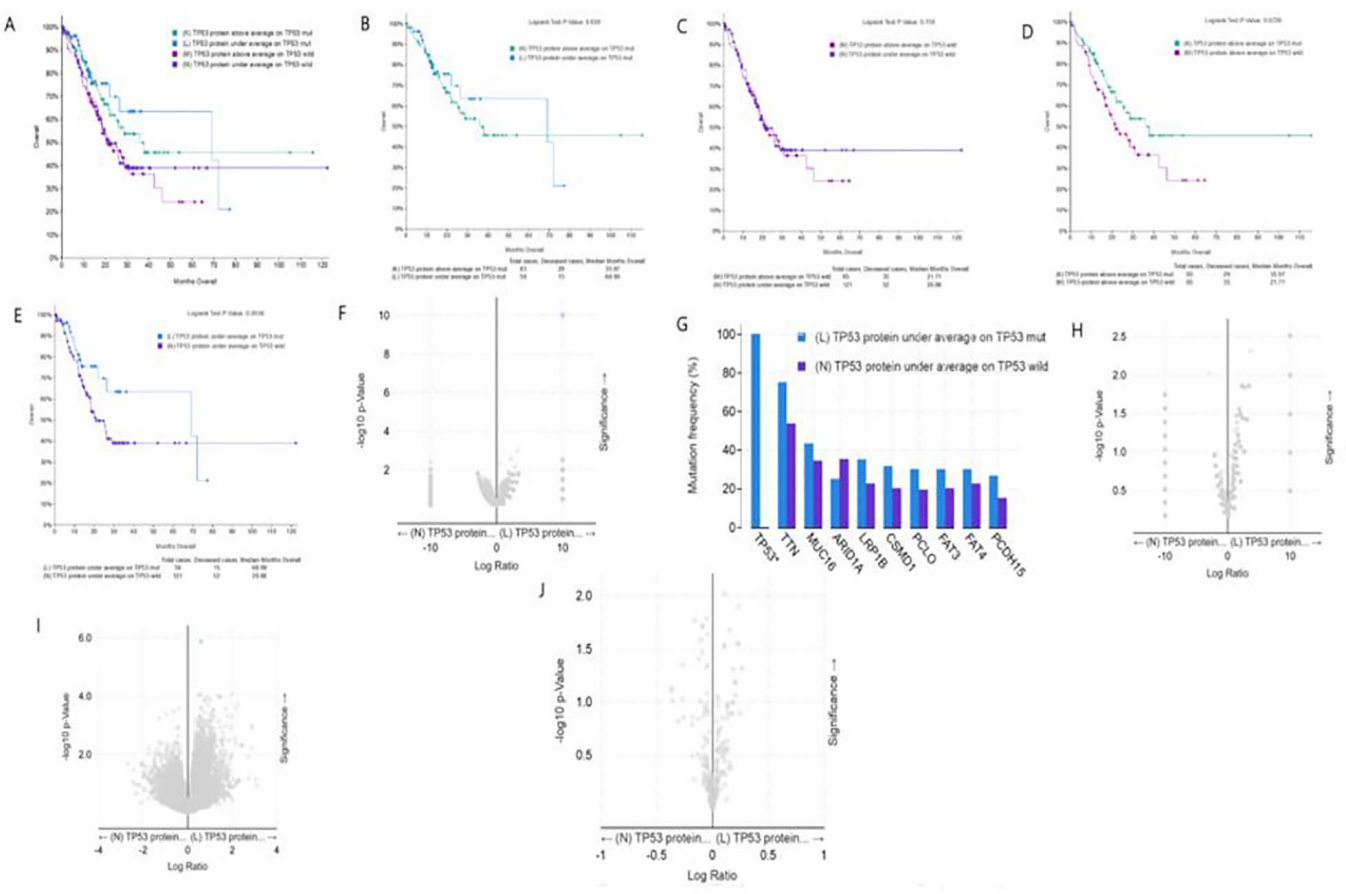
Clinical and genomic differences among 4 gastric cancer subgroups: high TP53 protein expression/TP53 mut, low TP53 protein expression/TP53 mut, high TP53 protein expression/TP53 WT, and low TP53 protein expression/TP53 WT. A: Maplan-Meier curve of overall survival months among 4 subgroups; B: Maplan-Meier curve between TP53 protein high expression(above average) in TP53 mut gastric patients and TP53 protein low expression(under average) in TP53 mut gastric patients (Longrank Test, P=0.639); C: Maplan-Meier curve between TP53 protein high expression(above average) in TP53 wild gastric patients and TP53 protein low expression(under average) in TP53 wild gastric patients (Longrank Test, P=0.759); D: Maplan-Meier curve between TP53 protein high expression(above average) in TP53 mut gastric patients and TP53 protein high expression(above average) in TP53 wild gastric patients (Longrank Test, P=0.0726); E: Maplan-Meier curve between TP53 protein low expression(under average) in TP53 mut gastric patients and TP53 protein low expression(under average) in TP53 wild gastric patients (Longrank Test, P=0.0558); F: Mutations between TP53 protein low expression(under average) in TP53 mut gastric patients and TP53 protein low expression(under average) in TP53 wild gastric patients (only TP53 mutation has difference); G: Mutation Frequency(%) in 3F; H: Copy number change between TP53 protein low expression(under average) in TP53 mut gastric patients and TP53 protein low expression(under average) in TP53 wild gastric patients (no difference); I: mRNA change between TP53 mRNA low expression(under average) in TP53 mut gastric patients and TP53 mRNA low expression(under average) in TP53 wild gastric patients (only RIF1 mRNA high expression in TP53 wild gastric patients, compared with TP53 mut gastric patients); J: protein expression between TP53 protein low expression(under average) in TP53 mut gastric patients and TP53 protein low expression(under average) in TP53 wild gastric patients (no difference). * means P<0.05.

Figure 5A shows the overall comparison of the four groups, i.e. TP53 mut/high expression; TP53 WT/high expression; TP53 mut/low expression; TP53 WT/low expression. In tumor patients with TP53 mut, the median survival time of patients with high expression of TP53 protein and low expression of TP53 protein was 35.97 and 68.99 months, respectively. Through statistical analysis, it was found that there was no statistical difference (Figure 5B). In tumor patients with TP53 WT, the median survival time of patients with high expression of TP53 protein and low expression of TP53 protein was 21.71 and 20.86 months, respectively, and there was no statistical difference (Figure 5C). In gastric cancer patients with high expression of TP53 protein, the median survival time of patients with TP53 mut and TP53 WT was 35.97 and 21.71 months respectively, and there was no statistical difference (Figure 5D). In gastric cancer patients with low expression of TP53 protein, the median survival time of patients with TP53 mut and TP53 WT was 68.99 and 20.86 months respectively. The p value is 0.058, which is very close to the threshold value of 0.05 (Figure 5E). Although there is no statistical difference, in fact, the median survival time of the two groups is quite different. In gastric cancer patients with low TP53 protein expression, the survival time of TP53 WT patients is less than one third of that of TP53 mut patients. In gastric cancer patients with low expression of TP53 protein, there is no difference in mutation between TP53 mut and TP53 WT patients except TP53 gene mutation (Figure 5F, 5G). In addition, there was no difference in copy number between these two groups (Figure 5H). In gastric cancer patients with low TP53 protein expression, the expression of RIF1 mRNA in TP53 WT patients was significantly higher than that in TP53 mut patients, suggesting that RIF1 may play a regulatory role in the low expression of TP53 protein in TP53 WT patients (Figure 5I). Among gastric cancer patients with low TP53 protein expression, there was no difference in TP53 protein content between TP53 mut and TP53 WT patients, and it was speculated that there may be a difference in protein activity (Figure 5J).

### 3.6 COX survival analysis of patients with gastric cancer

Because if multivariate COX survival analysis is used, almost all indicators have no statistical significance, so here we only adopt univariate COX survival analysis. COX regression analysis (Table 1) showed that the death risk of TP53 WT gastric cancer patients was 1.395 times that of TP53 mut patients. There is no difference in mortality among patients with gastric cancer of different genders. Age is directly proportional to mortality, and the older the age, the higher the risk of death. According to the cumulative percentage of patients' frequency, the age values at 33% and 66% were 60 and 70 years old, respectively, which were divided into three groups. Further grouping analysis indicated that the mortality of gastric cancer patients over 70 years old was 1.697 times higher than that of patients under 60 years old.

### 3.7 The COX model analysis of lymph node staging has statistical significance

Compared with gastric cancer patients without lymph node metastasis (N0), the death risk of gastric cancer patients with 1–2 lymph node metastasis (N1), 3–6 lymph node metastasis (N2) and 7 or more lymph node metastasis (N3) is 1.699, 1.677 and 2.751 times higher than that of N0 gastric cancer patients, respectively (Table 1). The detection rate of lymph nodes refers to the ratio of the number of metastatic lymph nodes to the total number of detected lymph nodes, which is more sensitive to the assessment of death risk of gastric cancer patients. Further grouping analysis showed that the death risk_of gastric cancer patients with lymph node detection rate of 20.1%-40%, 40.1%-60%, 60.1%-80% and 80.1%-100% was 1.557, 1.871, 5.603 and 3.462 times higher than that of gastric cancer patients with 0–20% of lymph node detection rate, respectively. It is suggested that patients with gastric cancer with a lymph node detection rate of 60.1%-80% have the highest risk of death.

### 3.8 The COX model analysis of tumor invasion depth staging was statistically significant

Gastric cancer can be divided into several stages according to the depth of invasion, namely T1, tumors invading the lamina propria, muscularis mucosae, or submucosa, T2, Tumors invading the muscularis propria; T3, Tumors penetrating the subserosal connective tissue without invasion of the visceral peritoneum or adjacent structures; and T4, tumors invading the serosa (visceral peritoneum) or adjacent structures. As shown in Table 1, compared with T1 gastric cancer patients, the death risk of T2, T3, and T4 gastric cancer patients is 7.306, 11.134 and 11.149 times of T1 gastric cancer patients, indicating that T3 and T4 gastric cancer patients have poor prognosis.

### 3.9 The COX model analysis of disease staging has statistical significance

Compared with patients with stage I gastric cancer, the death risk of patients with stage II, stage III and stage IV gastric cancer was 1.494, 2.413 and 3.978 times higher than that of patients with stage I gastric cancer, suggesting that the death risk is directly proportional to the disease stage.

### 3.10 The COX model analysis of tumor pathological types has statistical significance

Compared with patients with Diffuse Type Stomach Adenocarcinoma, the death risks of patients with Tubular Stomach Adenocarcinoma, Mucinous Stomach Adenocarcinoma, Papillary Stomach Adenocarcinoma and Signet Ring Cell Carcinoma were 1.040, 0.290, 1.047 and 2.742 times higher than those of patients with Diffuse Type Stomach Adenocarcinoma respectively. The results suggested that the pathological type of gastric cancer patients with the lowest risk of death is Mucinous Stomach Adenocarcinoma, and the prognosis of gastric cancer patients with Signet Ring Cell Carcinoma is the worst.

### 3.11 Univariate COX risk analysis of overall survival rate in patients with TP53 wild type and mut gastric cancer

COX regression analysis showed that there was no difference in the risk of death related to the ratio of male to female between TP53 mut and WT patients with gastric cancer (Table 2). The death risk in TP53 mut patients with gastric cancer was not related to age, indicating that advanced age was not a risk factor. However, the death risk of TP53 WT patients with gastric cancer increased with age, and the death risk of patients over 70 years old was 1.899 times that of patients under 60 years old. This suggests that the prognosis of elderly gastric cancer patients with TP53 WT is worse. The mortality risk of TP53 mut and TP53 WT patients with 7 or more lymph node metastasis (N3) was 3.451 and 2.361 times higher than that of patients without lymph node metastasis (N0), respectively. The detection rate of lymph nodes in TP53 mut gastric cancer patients was consistent with that in TP53 WT gastric cancer patients. In TP53 mut gastric cancer patients, the death risk of gastric cancer patients with lymph node detection rate of 60.1%-80% and 80.1%-100% was 5.675 and 3.498 times that of gastric cancer patients with lymph node detection rate of 0–20%, respectively. Among TP53 WT gastric cancer patients, the death risk of gastric cancer patients with lymph node detection rate of 60.1%-80% and 80.1%-100% was 5.587 and 3.506 times higher than those with lymph node detection rate of 0–20%, respectively.

The staging data of tumor invasion depth of TP53 WT instead of TP53 mut gastric cancer patients could construct COX model to predict the death of patients. The death risk of TP53 WT gastric cancer patients with T3 and T4 is 8.657 and 7.525 times higher than that of T1 gastric cancer patients, respectively. Compared with stage I gastric cancer patients, the death risk of TP53 mut gastric cancer patients increased significantly only in stage IV gastric cancer patients, which was 4.538 times higher than that of stage I patients. However, the death risk of TP53 WT patients with stage III and IV gastric cancer was 2.170 and 3.694 times higher than that of stage I gastric cancer patients, respectively. Lastly, there was almost no statistical difference in tumor pathological types between TP53 mut and WT gastric cancer patients.

## 4. Discussion

The NCI-MATCH trial (Molecular Analysis for Therapy Choice) has been developed towards precision medicine to identify drug-mutation pairs in a disease subgroup [20]. In precision medicine, genomic changes such as mutation and copy number have been employed as biomarkers to "individualize" different patient subsets with specific "targeted" drugs. Since TP53 is one of the most common mutations in cancer, its role in cancer biology has been comprehensively investigated to ascertain its biological importance in the development of cancer [14, 19, 21]. Nonetheless, the clinical significance of the relationship between TP53 mutation status and molecular/clinical categories such as OS and molecular subtypes remains to be answered [14, 19, 21]. In this spirit, our approach uses the TP53 mutation state to find significant associations with TP53 in clinical or molecular categories. It may be used to identify subtypes of certain diseases, for prognosis prediction or molecular targeted therapy.

In the study, we analyzed the TCGA database of gastric cancer patient data, stomach adenocarcinoma (TCGA, Firehose Legacy), which has clinical data, gene expression information and protein expression information of 478 gastric cancer patients. Of note, the TCGA Gastric Cancer Study Group [10] found that GC samples did not show any significant batch effects. Unexpectedly, our results prove that wild type TP53 is an indicator of poor prognosis of gastric cancer patients. Several lines of evidence support this conclusion. First, the median survival time of gastric cancer patients with TP53 mut and TP53 WT was 68.99 months and 26.08 months respectively. Longrank Test showed that there was a significant statistical difference between them (P<0.05), suggesting that the survival rate of gastric cancer patients with TP53 WT was significantly lower than that of TP53 mut (Figure 1). Second, compared with TP53 mut gastric cancer patients with low mRNA expression, TP53 WT patients with low mRNA expression have lower overall survival rate (P<0.05) (Figure 3). Third, the median survival time of gastric cancer patients with TP53 mut/low protein expression was 68.99 months, while that of patients with TP53 WT/low protein expression was 20.86 months, with a difference of 3 times, P=0.0558, which was close to the limit of P=0.05. We have reason to believe that with the increase of sample size, its P value may further decrease (Figure 5). Fourth, Table 1 provides further evidence that the death risk of TP53 WT gastric cancer patients is 1.395 times that of TP53 mut gastric cancer patients. Finally, Table 2 indicates that the death risk of TP53 mut gastric cancer patients is not related to age, and advanced age is not a risk factor. However, the death risk of TP53 WT patients with gastric cancer increases with age, and the death risk of patients over 70 years old is 1.899 times that of patients under 60 years old. These results suggest that the prognosis of elderly gastric cancer patients with TP53 WT is worse. Furthermore, among TP53 mut gastric cancer patients, only stage IV patients have higher death risk than stage I patients (4.538 times), while the death risk of TP53 WT patients with stage III and IV is higher than that of stage I patients (2.170 and 3.694 times respectively). Together, our results indicate that the prognosis of gastric cancer patients with WT TP53 is worse than that of patients with mutant TP53.

There are contradictory results about the prevalence of TP53 mutation and its relationship with the clinicopathological characteristics of gastric cancer [19], which may be partially due to differences in patients' physique or mutation detection methods. Because the mutation detection rate appears to be reliable even with the use of microbiopsy samples, previous data have shown that endoscopic biopsies are potentially useful for molecular analysis, exclusively for advanced patients who do not have information about surgically resected specimens [22]. TP53 mutations have long been thought to be associated with more aggressive phenotypes of various cancer types, including esophageal and colorectal cancer [23, 24]. Previous studies have also shown that GCs subsets with specific TP53 mutations (hot spots in the central core: R175, G245, R248, R273, R282) are related to poor overall survival and relapse-free survival [22]. The same type of mutation is also associated with the recurrence of gastric cancer. These results underline the importance of distinguishing TP53 mutations to differentiate GC patients for better clinical management. Since there has been a conflict between TP53 status and chemotherapy response in patients with gastric cancer [25–27], whether these mutations are able to predict chemotherapy response needs to be assessed in a larger cohort. Here, we obtained unexpected results by analyzing the data of gastric cancer patients in the TCGA database, namely, patients with mutant TP53 had a better prognosis than those with WT TP53. The mechanism behind this phenomenon may be that p53 activation preferentially induces a senescence program rather than cell death in TP53 WT cancer. Previous studies suggest that chemotherapy induces a dormant state such as cellular senescence in TP53 WT tumor cells, which has been revealed in patients [28], mouse models [29], and cell lines [29–31]. Various studies have shown that senescent and dormant cells promote relapse through producing cytokines that stimulate proliferation, survival, angiogenesis, as well as an increase in the number of cancer stem cells [32]. Our results show a link between WT TP53 and the prognosis of gastric cancer, which will provide substantial information, particularly for patients who cannot undergo surgical treatment because of disease progression. In conclusion, the status of TP53 may show significant correlation in clinical or molecular classification and wild type TP53 is an indicator of poor prognosis in patients with gastric cancer. TP53 wild type GC may require stronger adjuvant chemotherapy or frequent follow-up to avert recurrence after gastrectomy.

## Supplementary Material

2

## Figures and Tables

**Table 1:** COX hazard analysis of overall survival for gastric cancer patients.

Parameter	Univariate analysis			
	P	HR	95%CI	
			down	upper
TP53 status	0.048	1.395	1.004	1.941
Sex	0.264	1.211	0.866	1.694
Age	0.007	1.021	1.006	1.037
Age group	0.032	-	-	-
Age group (60–70 years old)	0.055	1.478	0.992	2.205
Age group (>70 years old)	0.01	1.697	1.135	2.537
Lymph Node Stage	<0.001	-	-	-
Lymph Node Stage(N1)	0.024	1.699	1.074	2.688
Lymph Node Stage(N2)	0.045	1.677	1.011	2.783
Lymph Node Stage(N3)	<0.001	2.751	1.735	4.361
Positive Lymph Node ratio	<0.001	4.818	2.951	7.866
Positive Lymph Node ratio group	<0.001	-	-	-
20.1%-40%	0.081	1.557	0.947	2.558
40.1%-60%	0.012	1.871	1.145	3.056
60.1%-80%	0	5.603	3.134	10.017
80.1%-100%	0	3.462	2.116	5.667
Tumor Stage	0.002	-	-	-
Tumor Stage(T2)	0.051	7.306	0.993	53.732
Tumor Stage(T3)	0.017	11.134	1.549	80.06
Tumor Stage(T4)	0.017	11.149	1.536	80.907
Disease Stage	<0.001	-	-	-
Disease Stage(Ⅱ)	0.23	1.494	0.776	2.879
Disease Stage(Ⅲ)	0.005	2.413	1.311	4.443
Disease Stage(Ⅳ)	0	3.978	1.988	7.962
Cancer Type	0.018	-	-	-
Tubular Stomach Adenocarcinoma	0.885	1.04	0.611	1.771
Mucinous Stomach Adenocarcinoma	0.042	0.29	0.088	0.959
Papillary Stomach Adenocarcinoma	0.94	1.047	0.316	3.467
Signet Ring Cell Carcinoma	0.009	2.742	1.281	5.868

**Table 2:** Univariate COX hazard analysis of overall survival for TP53 wild and mut gastric cancer patients.

Parameter	TP53 mut gastric cancer patients				TP53 wild gastric cancer patients			
	P	HR	95%CI		P	HR	95%CI	
			down	upper			down	upper
Sex	0.585	0.85	0.475	1.521	0.347	0.82	0.542	1.24
Age	0.146	1.019	0.993	1.045	0.014	1.025	1.005	1.046
Age group	0.452				0.044			
Age group (60–70 years old)	0.254	1.499	0.748	3.005	0.168	1.413	0.865	2.31
Age group (>70 years old)	0.267	1.472	0.744	2.913	0.013	1.899	1.147	3.145
Lymph Node Stage	0.042				0.017			
Lymph Node Stage(N1)	0.11	1.909	0.863	4.223	0.12	1.562	0.89	2.742
Lymph Node Stage(N2)	0.165	1.839	0.779	4.343	0.134	1.619	0.863	3.04
Lymph Node Stage(N3)	0.002	3.451	1.577	7.556	0.003	2.362	1.331	4.192
Positive Lymph Node ratio	<0.001	4.833	2.12	11.018	<0.001	4.906	2.66	9.05
Positive Lymph Node ratio group	0.002				<0.001			
20.1%–40%	0.394	1.47	0.606	3.565	0.121	1.613	0.881	2.952
40.1%–60%	0.064	2.163	0.956	4.89	0.06	1.809	0.976	3.352
60.1%–80%	<0.001	5.675	2.213	14.557	<0.001	5.587	2.646	11.798
80.1%–100%	0.003	3.498	1.535	7.97	<0.001	3.506	1.889	6.508
Tumor Stage	0.136				0.048			
Tumor Stage(T2)	0.903	>9000	0	>E^60^	0.063	6.807	0.902	51.367
Tumor Stage(T3)	0.898	>9000	0	>E^60^	0.033	8.657	1.196	62.678
Tumor Stage(T4)	0.895	>9000	0	>E^60^	0.047	7.525	1.025	55.269
Disease Stage	0.023				0.009			
Disease Stage(Ⅱ)	0.467	1.51	0.497	4.594	0.293	1.551	0.685	3.512
Disease Stage(Ⅲ)	0.06	2.771	0.959	8.009	0.043	2.17	1.025	4.591
Disease Stage(Ⅳ)	0.014	4.538	1.352	15.235	0.003	3.694	1.576	8.662
Cancer Type	0.078				0.224			
Tubular Stomach Adenocarcinoma	0.065	2.658	0.942	7.5	0.865	0.958	0.583	1.574
Mucinous Stomach Adenocarcinoma	0.37	1.716	0.527	5.594	0.84	0.94	0.512	1.724
Papillary Stomach Adenocarcinoma	0.887	1.132	0.207	6.203	0.035	0.115	0.016	0.855
Signet Ring Cell Carcinoma	0.5	2.129	0.237	19.101	0.8	0.829	0.195	3.535
