# Supplementary material for: Wild-Type TP53 Predicts Poor Prognosis in Patients with Gastric Cancer
Source: J Cancer Sci Clin Ther. Author manuscript; Available in PMC 2021 Dec 22. (PMC8694034; doi:10.26502/jcsct.50790107)
Supplement: 2 [file NIHMS1750514-supplement-2.pdf]

J Cancer Sci Clin Ther 2021; 5 (1): 134-153 *DOI: 10.26502/jcsct.50790107*

Research Article

Wild-Type TP53 Predicts Poor Prognosis in Patients with Gastric Cancer

Wenhong Deng[1, 2], Qiongyu Hao[2], Jaydutt Vadgama[2*], Yong Wu[2*]

[1]Department of General Surgery, Renmin Hospital of Wuhan University, Wuhan, China

[2]Division of Cancer Research and Training, Department of Internal Medicine, Charles Drew University of Medicine and Science, CA, USA

[*]**Corresponding Authors:** Jaydutt V Vadgama, Department of Internal Medicine, Charles R. Drew University of Medicine and Science, 1748 E. 118th Street, Los Angeles, CA 90059, USA, Tel: 323-563-9397; Fax: 323-563-4889; E-mail: jayvadgama@cdrewu.edu

Yong Wu, Department of Internal Medicine, Charles Drew University of Medicine and Science, David Geffen UCLA School of Medicine and UCLA Jonsson Comprehensive Cancer Center, Los Angeles, CA, USA, E-mail: yongwu@cdrewu.edu

Received: 17 December 2020; **Accepted:** 11 January 2021; **Published**: 18 March 2021

Citation: Wenhong Deng, Qiongyu Hao, Jaydutt Vadgama, Yong Wu. Wild-Type TP53 Predicts Poor Prognosis in Patients with Gastric Cancer. Journal of Cancer Science and Clinical Therapeutics 5 (2021): 134-153.

Supplemental data

ene	Cytoband	(A) TP53 m	(B) TP53 wi	(A) TP53 m	(B) TP53 wi	Log Ratio	p-Value	q-Value	Higher exp-ression in
MDM2	12q15	10.46	11.28	0.58	0.94	-0.82	1.25E-24	1.65E-20	(B) TP53 wild in GC
DDB2	11p11.2	8.3	8.92	0.6	0.72	-0.62	1.35E-19	8.92E-16	(B) TP53 wild in GC
TENT2	5q14.1	9.6	9.92	0.42	0.41	-0.32	1.87E-13	4.94E-10	(B) TP53 wild in GC
FAS	10q23.31	8.08	8.9	1.08	1.1	-0.81	2.96E-13	5.57E-10	(B) TP53 wild in GC
RPS27L	15q22.2	9.95	10.48	0.68	0.75	-0.52	5.64E-13	8.26E-10	(B) TP53 wild in GC
ZMAT3	3q26.32	7.93	8.47	0.69	0.83	-0.54	1.64E-12	1.95E-09	(B) TP53 wild in GC
PDE4D	5q11.2-q12	8.65	9.38	1.01	1.04	-0.74	1.98E-12	2.01E-09	(B) TP53 wild in GC
CCNG1	5q34	10.61	11.09	0.7	0.65	-0.48	4.64E-12	3.65E-09	(B) TP53 wild in GC
SPATA18	4q12	6.39	7.32	1.29	1.43	-0.93	1.38E-11	9.08E-09	(B) TP53 wild in GC
AP3B1	5q14.1	10.22	10.49	0.44	0.4	-0.28	2.23E-10	1.13E-07	(B) TP53 wild in GC
RPL22L1	3q26.2	9.12	9.93	1.33	1.26	-0.81	8.24E-10	3.10E-07	(B) TP53 wild in GC
JAK2	9p24.1	8.95	9.58	0.98	1.06	-0.63	9.10E-10	3.21E-07	(B) TP53 wild in GC
TIGAR	12p13.32	8.75	9.13	0.61	0.61	-0.38	9.21E-10	3.21E-07	(B) TP53 wild in GC
WDR41	5q13.3-q14	8.61	8.9	0.53	0.41	-0.3	1.91E-09	5.75E-07	(B) TP53 wild in GC

RASA1	5q14.3	9.32	9.63	0.52	0.49	-0.31	2.21E-09	6.21E-07	(B) TP53 wild in GC
FDXR	17q25.1	7.86	8.41	0.85	0.99	-0.55	3.13E-09	8.10E-07	(B) TP53 wild in GC
CLEC2D	12p13.31	8.23	8.66	0.74	0.7	-0.43	4.26E-09	9.52E-07	(B) TP53 wild in GC
XPC	3p25.1	9.41	9.67	0.44	0.41	-0.25	4.49E-09	9.87E-07	(B) TP53 wild in GC
TNFAIP8	5q23.1	8.32	8.82	0.82	0.89	-0.5	6.47E-09	1.30E-06	(B) TP53 wild in GC
PARP8	5q11.1	8.54	8.98	0.84	0.63	-0.44	1.44E-08	2.43E-06	(B) TP53 wild in GC
RRM2B	8q22.3	9.5	9.84	0.54	0.67	-0.34	1.62E-08	2.67E-06	(B) TP53 wild in GC
TRIM22	11p15.4	9.51	10.19	1.14	1.26	-0.68	1.90E-08	2.94E-06	(B) TP53 wild in GC
AHCYL2	7q32.1	10.13	10.84	1.25	1.26	-0.7	3.02E-08	4.07E-06	(B) TP53 wild in GC
ZFP2	5q35.3	3.61	4.26	1.19	1.1	-0.65	3.38E-08	4.48E-06	(B) TP53 wild in GC
FBXL5	4p15.32	10.45	10.72	0.43	0.53	-0.27	3.50E-08	4.48E-06	(B) TP53 wild in GC
YTHDC2	5q22.2	9.2	9.54	0.63	0.58	-0.34	4.37E-08	5.39E-06	(B) TP53 wild in GC
CIRBP	19p13.3	10.52	10.82	0.54	0.58	-0.3	7.28E-08	7.86E-06	(B) TP53 wild in GC
AEN	15q26.1	9.19	9.54	0.62	0.71	-0.35	1.19E-07	1.17E-05	(B) TP53 wild in GC
ZFYVE16	5q14.1	9.64	9.9	0.48	0.52	-0.26	1.35E-07	1.28E-05	(B) TP53 wild in GC
MRPS27	5q13.2	9.95	10.21	0.49	0.48	-0.26	1.45E-07	1.33E-05	(B) TP53 wild in GC

AGGF1	5q13.3	9.56	9.79	0.45	0.4	-0.23	1.81E-07	1.58E-05	(B) TP53 wild in GC
CNOT8	5q33.2	9.78	9.96	0.34	0.34	-0.18	1.86E-07	1.62E-05	(B) TP53 wild in GC
TTC3	21q22.13	11.54	11.81	0.47	0.57	-0.27	1.96E-07	1.68E-05	(B) TP53 wild in GC
UBLCP1	5q33.3	8.91	9.16	0.5	0.44	-0.25	2.08E-07	1.75E-05	(B) TP53 wild in GC
RNF145	5q33.3	10.42	10.74	0.61	0.64	-0.33	2.20E-07	1.81E-05	(B) TP53 wild in GC
HSPA4L	4q28.1	6.68	7.61	1.88	1.62	-0.93	2.25E-07	1.84E-05	(B) TP53 wild in GC
EEF1A1	6q13	15.69	15.96	0.5	0.51	-0.26	2.59E-07	2.05E-05	(B) TP53 wild in GC
CNOT6L	4q21.1	9.95	10.22	0.53	0.52	-0.27	2.91E-07	2.24E-05	(B) TP53 wild in GC
RPS23	5q14.2	12.73	13.06	0.64	0.59	-0.32	3.00E-07	2.28E-05	(B) TP53 wild in GC
RABEP1	17p13.2	9.78	10.04	0.48	0.5	-0.25	3.04E-07	2.29E-05	(B) TP53 wild in GC
RPL3	22q13.1	14.06	14.35	0.57	0.55	-0.29	3.52E-07	2.52E-05	(B) TP53 wild in GC
ARL6IP5	3p14.1	11.2	11.5	0.6	0.56	-0.3	3.91E-07	2.69E-05	(B) TP53 wild in GC
FBXO38	5q32	9.3	9.52	0.45	0.42	-0.22	4.35E-07	2.91E-05	(B) TP53 wild in GC
NCOA4	10q11.22	12.22	12.45	0.47	0.42	-0.23	5.09E-07	3.30E-05	(B) TP53 wild in GC
C21ORF91	21q21.1	7.95	8.28	0.67	0.64	-0.33	5.14E-07	3.32E-05	(B) TP53 wild in GC
RIOK2	5q15	8.45	8.69	0.51	0.44	-0.25	5.31E-07	3.38E-05	(B) TP53 wild in GC

ITGB7	12q13.13	7.4	7.98	1.1	1.18	-0.57	5.44E-07	3.40E-05	(B) TP53 wild in GC
CYLD	16q12.1	9.44	9.77	0.65	0.67	-0.33	5.94E-07	3.58E-05	(B) TP53 wild in GC
TTC19	17p12	9.61	9.91	0.6	0.58	-0.3	6.35E-07	3.77E-05	(B) TP53 wild in GC
RPL23AP53	8p23.3	6.97	7.34	0.73	0.74	-0.37	6.95E-07	4.02E-05	(B) TP53 wild in GC
ARHGEF3	3p14.3	9.19	9.49	0.53	0.65	-0.29	7.05E-07	4.04E-05	(B) TP53 wild in GC
ADPRM	17p13.1	6.73	7.02	0.58	0.55	-0.28	7.08E-07	4.04E-05	(B) TP53 wild in GC
USP22	17p11.2	11.71	11.96	0.53	0.46	-0.25	7.36E-07	4.14E-05	(B) TP53 wild in GC
TMED7	5q22.3	11.21	11.46	0.49	0.5	-0.25	7.43E-07	4.14E-05	(B) TP53 wild in GC
PAFAH1B1	17p13.3	11.05	11.26	0.4	0.43	-0.21	7.85E-07	4.27E-05	(B) TP53 wild in GC
FCHO2	5q13.2	9.62	9.94	0.66	0.64	-0.33	8.05E-07	4.35E-05	(B) TP53 wild in GC
MBNL1	3q25.1-q25	11.75	12.03	0.54	0.59	-0.28	8.59E-07	4.62E-05	(B) TP53 wild in GC
FUT10	8p12	7.31	7.65	0.71	0.7	-0.35	1.01E-06	5.21E-05	(B) TP53 wild in GC
TMTC2	12q21.31	8.47	8.89	0.86	0.87	-0.43	1.02E-06	5.22E-05	(B) TP53 wild in GC
PIK3R1	5q13.1	10.22	10.6	0.75	0.81	-0.38	1.03E-06	5.26E-05	(B) TP53 wild in GC
FAM13B	5q31.2	8.99	9.34	0.66	0.77	-0.35	1.04E-06	5.28E-05	(B) TP53 wild in GC
PGGT1B	5q22.3	8.09	8.34	0.52	0.49	-0.25	1.06E-06	5.32E-05	(B) TP53 wild in GC

RIC1	9p24.1	9.01	9.34	0.7	0.63	-0.33	1.12E-06	5.44E-05	(B) TP53 wild in GC
GPBP1	5q11.2	10.31	10.51	0.41	0.41	-0.2	1.18E-06	5.71E-05	(B) TP53 wild in GC
DYRK4	12p13.32	7.96	8.32	0.82	0.62	-0.36	1.33E-06	6.18E-05	(B) TP53 wild in GC
SLC25A46	5q22.1	8.92	9.26	0.77	0.59	-0.34	1.52E-06	6.92E-05	(B) TP53 wild in GC
RFX7	15q21.3	9.34	9.63	0.59	0.62	-0.29	1.61E-06	7.22E-05	(B) TP53 wild in GC
ZNF561	19p13.2	8.75	9.02	0.58	0.54	-0.27	1.63E-06	7.30E-05	(B) TP53 wild in GC
EIF4B	12q13.13	12.29	12.51	0.47	0.47	-0.23	1.71E-06	7.54E-05	(B) TP53 wild in GC
METTL25	12q21.31	5.87	6.15	0.61	0.51	-0.28	1.72E-06	7.54E-05	(B) TP53 wild in GC
TNFRSF10C	8p21.3	5.41	6.07	1.22	1.57	-0.66	1.72E-06	7.54E-05	(B) TP53 wild in GC
CRTAP	3p22.3	11.58	11.88	0.62	0.64	-0.3	1.73E-06	7.54E-05	(B) TP53 wild in GC
TMEM30A	6q14.1	11.51	11.74	0.46	0.49	-0.23	1.87E-06	7.95E-05	(B) TP53 wild in GC
EEF2	19p13.3	15.44	15.73	0.62	0.58	-0.29	2.00E-06	8.33E-05	(B) TP53 wild in GC
RBFOX2	22q12.3	10.59	10.85	0.49	0.62	-0.26	2.11E-06	8.55E-05	(B) TP53 wild in GC
DGLUCY	14q32.11	9.03	9.35	0.66	0.71	-0.32	2.22E-06	8.91E-05	(B) TP53 wild in GC
MAP2K4	17p12	9.24	9.5	0.61	0.49	-0.27	2.31E-06	9.17E-05	(B) TP53 wild in GC
SMG6	17p13.3	9.74	9.97	0.46	0.49	-0.22	2.46E-06	9.52E-05	(B) TP53 wild in GC

CEP120	5q23.2	8.77	9.03	0.57	0.52	-0.26	2.63E-06	1.00E-04	(B) TP53 wild in GC
CYB5D2	17p13.2	7.7	7.97	0.59	0.55	-0.27	2.84E-06	1.08E-04	(B) TP53 wild in GC
WDR82	3p21.2	10.96	11.13	0.39	0.34	-0.17	2.86E-06	1.08E-04	(B) TP53 wild in GC
KLRK1	12p13.2	4.96	5.77	1.7	1.78	-0.82	2.94E-06	1.10E-04	(B) TP53 wild in GC
ANKFY1	17p13.2	10.7	10.93	0.5	0.5	-0.23	3.43E-06	1.26E-04	(B) TP53 wild in GC
CELF2	10p14	8.71	9.34	1.27	1.48	-0.64	3.43E-06	1.26E-04	(B) TP53 wild in GC
SNRK	3p22.1	9.85	10.12	0.54	0.59	-0.26	3.48E-06	1.27E-04	(B) TP53 wild in GC
DIP2A	21q22.3	9.49	9.71	0.47	0.49	-0.22	3.75E-06	1.34E-04	(B) TP53 wild in GC
BTF3	5q13.2	12.26	12.52	0.56	0.56	-0.26	3.75E-06	1.34E-04	(B) TP53 wild in GC
SNX18	5q11.2	9.32	9.63	0.67	0.66	-0.31	4.03E-06	1.41E-04	(B) TP53 wild in GC
REEP5	5q22.2	11.27	11.5	0.49	0.47	-0.22	4.09E-06	1.42E-04	(B) TP53 wild in GC
MBLAC2	5q14.3	7.31	7.63	0.71	0.62	-0.31	4.28E-06	1.47E-04	(B) TP53 wild in GC
TMEM71	8q24.22	4.54	5.16	1.3	1.43	-0.62	4.57E-06	1.53E-04	(B) TP53 wild in GC
IPCEF1	6q25.2	5.56	6.16	1.28	1.31	-0.6	4.57E-06	1.53E-04	(B) TP53 wild in GC
ARPP19	15q21.2	11.83	12.04	0.47	0.44	-0.21	5.00E-06	1.65E-04	(B) TP53 wild in GC
NLRC3	16p13.3	6.94	7.48	1.13	1.23	-0.54	5.00E-06	1.65E-04	(B) TP53 wild in GC

UBASH3A	21q22.3	4.53	5.26	1.59	1.58	-0.73	5.26E-06	1.71E-04	(B) TP53 wild in GC
TXNDC15	5q31.1	9.42	9.66	0.53	0.52	-0.24	5.52E-06	1.78E-04	(B) TP53 wild in GC
IPO11	5q12.1	8.87	9.09	0.48	0.47	-0.22	5.55E-06	1.78E-04	(B) TP53 wild in GC
CSNK1A1	5q32	11.82	12	0.43	0.33	-0.18	6.13E-06	1.92E-04	(B) TP53 wild in GC
PJA2	5q21.3	10.92	11.22	0.62	0.72	-0.3	6.49E-06	2.00E-04	(B) TP53 wild in GC
MSH3	5q14.1	8.48	8.72	0.53	0.53	-0.24	6.64E-06	2.04E-04	(B) TP53 wild in GC
RBMS2	12q13.3	10.07	10.39	0.72	0.69	-0.32	6.86E-06	2.08E-04	(B) TP53 wild in GC
AKAP10	17p11.2	8.42	8.65	0.54	0.49	-0.24	7.10E-06	2.15E-04	(B) TP53 wild in GC
TET2	4q24	9.32	9.6	0.62	0.62	-0.28	7.32E-06	2.20E-04	(B) TP53 wild in GC
ANKRA2	5q13.2	7.83	8.04	0.48	0.44	-0.21	7.33E-06	2.20E-04	(B) TP53 wild in GC
CD96	3q13.13-q1	7.07	7.71	1.43	1.42	-0.64	7.57E-06	2.25E-04	(B) TP53 wild in GC
ARHGEF6	Xq26.3	8.17	8.67	1.08	1.15	-0.5	7.63E-06	2.26E-04	(B) TP53 wild in GC
CHD1	5q15-q21.1	9.76	10.01	0.6	0.5	-0.25	8.16E-06	2.38E-04	(B) TP53 wild in GC
CCDC50	3q28	10.44	10.72	0.6	0.66	-0.28	8.29E-06	2.42E-04	(B) TP53 wild in GC
FAM151B	5q14.1	3.77	4.14	0.81	0.82	-0.37	8.57E-06	2.48E-04	(B) TP53 wild in GC
REV3L	6q21	9.48	9.79	0.65	0.72	-0.31	8.81E-06	2.54E-04	(B) TP53 wild in GC

PHAX	5q23.2	8.37	8.54	0.39	0.39	-0.17	9.24E-06	2.64E-04	(B) TP53 wild in GC
GALNT10	5q33.2	10.24	10.58	0.75	0.78	-0.34	9.33E-06	2.66E-04	(B) TP53 wild in GC
FEM1C	5q22.3	9.68	9.92	0.57	0.53	-0.25	9.64E-06	2.73E-04	(B) TP53 wild in GC
PRKX	Xp22.33	9.12	9.5	0.91	0.76	-0.38	9.81E-06	2.77E-04	(B) TP53 wild in GC
TRIM5	11p15.4	8.61	8.9	0.7	0.56	-0.29	9.82E-06	2.77E-04	(B) TP53 wild in GC
ABHD5	3p21.33	8.85	9.08	0.49	0.53	-0.23	9.87E-06	2.77E-04	(B) TP53 wild in GC
THEMIS	6q22.33	5.1	5.84	1.64	1.69	-0.74	1.02E-05	2.85E-04	(B) TP53 wild in GC
ITK	5q33.3	6.3	6.99	1.56	1.56	-0.69	1.05E-05	2.87E-04	(B) TP53 wild in GC
TK2	16q21	8.68	8.94	0.6	0.6	-0.27	1.05E-05	2.87E-04	(B) TP53 wild in GC
THG1L	5q33.3	7.38	7.63	0.59	0.53	-0.25	1.08E-05	2.92E-04	(B) TP53 wild in GC
RANBP6	9p24.1	8.44	8.74	0.71	0.64	-0.3	1.18E-05	3.14E-04	(B) TP53 wild in GC
GVINP1	11p15.4	6.67	7.31	1.45	1.47	-0.64	1.21E-05	3.20E-04	(B) TP53 wild in GC
GIT2	12q24.11	10	10.23	0.51	0.55	-0.23	1.27E-05	3.32E-04	(B) TP53 wild in GC
NSA2	5q13.3	10.23	10.5	0.64	0.61	-0.28	1.29E-05	3.35E-04	(B) TP53 wild in GC
PRKCQ	10p15.1	5.23	5.97	1.76	1.59	-0.74	1.31E-05	3.38E-04	(B) TP53 wild in GC
CHD3	17p13.1	11.18	11.51	0.76	0.74	-0.33	1.37E-05	3.49E-04	(B) TP53 wild in GC

TMEM167	5q14.2	10.57	10.78	0.47	0.48	-0.21	1.40E-05	3.56E-04	(B) TP53 wild in GC
TBC1D12	10q23.33	8.44	8.67	0.53	0.52	-0.23	1.40E-05	3.56E-04	(B) TP53 wild in GC
AGAP2	12q14.1	6.92	7.46	1.19	1.3	-0.54	1.42E-05	3.59E-04	(B) TP53 wild in GC
SECISBP2L	15q21.1	9.82	10.08	0.55	0.62	-0.25	1.43E-05	3.62E-04	(B) TP53 wild in GC
UBE2G1	17p13.2	10.46	10.63	0.39	0.38	-0.17	1.46E-05	3.65E-04	(B) TP53 wild in GC
PHLDA3	1q32.1	8.85	9.41	1.29	1.28	-0.56	1.47E-05	3.68E-04	(B) TP53 wild in GC
TTC23	15q26.3	7.77	8.1	0.75	0.77	-0.33	1.50E-05	3.74E-04	(B) TP53 wild in GC
KIAA1143	3p21.31	9.48	9.68	0.47	0.45	-0.2	1.54E-05	3.81E-04	(B) TP53 wild in GC
SCAMP1	5q14.1	10.06	10.27	0.47	0.54	-0.22	1.58E-05	3.87E-04	(B) TP53 wild in GC
PDE5A	4q26	9.11	9.6	1.07	1.22	-0.49	1.62E-05	3.96E-04	(B) TP53 wild in GC
COL4A3BP	5q13.3	9.54	9.74	0.46	0.47	-0.2	1.63E-05	3.97E-04	(B) TP53 wild in GC
MAP4	3p21.31	12.28	12.5	0.49	0.54	-0.22	1.63E-05	3.97E-04	(B) TP53 wild in GC
FUT8	14q23.3	10	10.3	0.67	0.68	-0.29	1.68E-05	4.06E-04	(B) TP53 wild in GC
RBSN	3p25.1	9.22	9.45	0.48	0.55	-0.22	1.70E-05	4.07E-04	(B) TP53 wild in GC
NUDT6	4q28.1	5.25	5.68	1.04	0.93	-0.43	1.70E-05	4.07E-04	(B) TP53 wild in GC
CDC42SE2	5q31.1	10.41	10.68	0.62	0.61	-0.26	1.73E-05	4.13E-04	(B) TP53 wild in GC

ZNF132	19q13.43	5.17	5.58	0.92	0.96	-0.4	1.74E-05	4.13E-04	(B) TP53 wild in GC
CCNH	5q14.3	8.9	9.1	0.5	0.43	-0.2	1.80E-05	4.26E-04	(B) TP53 wild in GC
DMXL1	5q23.1	9.55	9.83	0.64	0.68	-0.28	1.81E-05	4.28E-04	(B) TP53 wild in GC
ZNF557	19p13.2	7.39	7.66	0.67	0.56	-0.27	1.82E-05	4.28E-04	(B) TP53 wild in GC
APC	5q22.2	9.63	9.93	0.73	0.66	-0.3	1.87E-05	4.37E-04	(B) TP53 wild in GC
PRMT2	21q22.3	9.82	10.04	0.52	0.48	-0.22	1.93E-05	4.50E-04	(B) TP53 wild in GC
SEC31B	10q24.31	5.62	6.17	1.26	1.3	-0.55	1.94E-05	4.51E-04	(B) TP53 wild in GC
GAB3	Xq28	6.19	6.64	1.01	1.14	-0.46	2.00E-05	4.60E-04	(B) TP53 wild in GC
TIFA	4q25	7.5	7.76	0.6	0.62	-0.26	2.11E-05	4.80E-04	(B) TP53 wild in GC
CAST	5q15	11.92	12.18	0.65	0.6	-0.27	2.13E-05	4.81E-04	(B) TP53 wild in GC
PTPN22	1p13.2	6.87	7.38	1.17	1.24	-0.51	2.21E-05	4.95E-04	(B) TP53 wild in GC
SP1	12q13.13	11.87	12.03	0.36	0.4	-0.16	2.24E-05	5.01E-04	(B) TP53 wild in GC
MITF	3p13	7.79	8.28	1.12	1.2	-0.49	2.27E-05	5.05E-04	(B) TP53 wild in GC
METTL14	4q26	7.73	7.92	0.44	0.43	-0.18	2.29E-05	5.06E-04	(B) TP53 wild in GC
LINS1	15q26.3	8.01	8.23	0.53	0.53	-0.23	2.31E-05	5.08E-04	(B) TP53 wild in GC
PCGF5	10q23.32	10.8	11.02	0.51	0.5	-0.21	2.34E-05	5.13E-04	(B) TP53 wild in GC

TLR3	4q35.1	7.3	7.86	1.37	1.25	-0.56	2.43E-05	5.31E-04	(B) TP53 wild in GC
DELE1	5q31.3	9.65	9.83	0.47	0.39	-0.18	2.45E-05	5.33E-04	(B) TP53 wild in GC
NDUFS4	5q11.2	8.85	9.1	0.61	0.56	-0.25	2.51E-05	5.42E-04	(B) TP53 wild in GC
WHAMM	15q25.2	8.3	8.49	0.45	0.47	-0.19	2.62E-05	5.62E-04	(B) TP53 wild in GC
HGSNAT	8p11.21-p1	10.87	11.12	0.6	0.58	-0.25	2.62E-05	5.62E-04	(B) TP53 wild in GC
ITGAE	17p13.2	7.95	8.26	0.72	0.74	-0.31	2.66E-05	5.64E-04	(B) TP53 wild in GC
SLC6A14	Xq23	7.67	8.85	2.89	2.64	-1.18	2.75E-05	5.77E-04	(B) TP53 wild in GC
UTRN	6q24.2	10.47	10.86	0.9	0.94	-0.39	2.75E-05	5.78E-04	(B) TP53 wild in GC
OGA	10q24.32	10.96	11.18	0.5	0.56	-0.22	2.81E-05	5.88E-04	(B) TP53 wild in GC
KLRG1	12p13.31	4	4.53	1.24	1.24	-0.52	2.86E-05	5.95E-04	(B) TP53 wild in GC
ARRDC3	5q14.3	10.2	10.5	0.73	0.73	-0.31	2.90E-05	6.04E-04	(B) TP53 wild in GC
SLF2	10q24.31	9.19	9.42	0.52	0.55	-0.22	3.00E-05	6.21E-04	(B) TP53 wild in GC
MECOM	3q26.2	10.7	11.22	1.39	1.01	-0.52	3.11E-05	6.40E-04	(B) TP53 wild in GC
IL16	15q25.1	8	8.51	1.21	1.27	-0.52	3.15E-05	6.46E-04	(B) TP53 wild in GC
FCHSD2	11q13.4	9.24	9.51	0.65	0.65	-0.27	3.19E-05	6.50E-04	(B) TP53 wild in GC
ETV6	12p13.2	10.6	10.8	0.51	0.44	-0.2	3.21E-05	6.52E-04	(B) TP53 wild in GC

CXCR6	3p21.31	6.59	7.16	1.34	1.38	-0.57	3.25E-05	6.58E-04	(B) TP53 wild in GC
FAM160B1	10q25.3	9.31	9.53	0.49	0.55	-0.21	3.27E-05	6.62E-04	(B) TP53 wild in GC
NOA1	4q12	9.69	9.87	0.43	0.44	-0.18	3.33E-05	6.69E-04	(B) TP53 wild in GC
GANC	15q15.1	8.32	8.56	0.55	0.58	-0.24	3.34E-05	6.69E-04	(B) TP53 wild in GC
BCL2	18q21.33	7.51	7.97	1.1	1.11	-0.46	3.40E-05	6.78E-04	(B) TP53 wild in GC
CCDC125	5q13.2	7.66	7.93	0.71	0.59	-0.28	3.41E-05	6.79E-04	(B) TP53 wild in GC
ACKR4	3q22.1	6.6	7.31	1.68	1.76	-0.71	3.51E-05	6.91E-04	(B) TP53 wild in GC
GPR34	Xp11.4	6.69	7.21	1.16	1.37	-0.52	3.51E-05	6.91E-04	(B) TP53 wild in GC
CD8A	2p11.2	7.29	7.99	1.66	1.71	-0.7	3.58E-05	7.03E-04	(B) TP53 wild in GC
C4ORF3	4q26	10.47	10.71	0.56	0.6	-0.24	3.61E-05	7.07E-04	(B) TP53 wild in GC
PITPNM3	17p13.2-p1	7.51	8.2	1.63	1.67	-0.68	3.65E-05	7.09E-04	(B) TP53 wild in GC
CD2	1p13.1	7.78	8.35	1.39	1.39	-0.58	3.68E-05	7.15E-04	(B) TP53 wild in GC
ZNF33B	10q11.21	9.3	9.55	0.63	0.62	-0.26	3.71E-05	7.18E-04	(B) TP53 wild in GC
SMARCA2	9p24.3	10.24	10.6	0.87	0.83	-0.35	3.80E-05	7.28E-04	(B) TP53 wild in GC
CD3E	11q23.3	8.09	8.67	1.41	1.42	-0.58	3.80E-05	7.28E-04	(B) TP53 wild in GC
GABRP	5q35.1	7.5	8.91	3.41	3.39	-1.41	3.84E-05	7.31E-04	(B) TP53 wild in GC

ARHGAP26	5q31.3	10.06	10.39	0.79	0.86	-0.34	3.85E-05	7.32E-04	(B) TP53 wild in GC
SRR	17p13.3	6.94	7.23	0.67	0.72	-0.28	3.87E-05	7.35E-04	(B) TP53 wild in GC
STX17	9q31.1	9.69	9.87	0.43	0.41	-0.17	3.93E-05	7.43E-04	(B) TP53 wild in GC
CYFIP2	5q33.3	8.91	9.41	1.25	1.15	-0.5	3.96E-05	7.48E-04	(B) TP53 wild in GC
SLMAP	3p14.3	10.56	10.89	0.72	0.89	-0.33	3.99E-05	7.52E-04	(B) TP53 wild in GC
METTL16	17p13.3	9.34	9.51	0.43	0.43	-0.18	4.08E-05	7.62E-04	(B) TP53 wild in GC
ITGA4	2q31.3	8.62	9.09	1.14	1.1	-0.46	4.11E-05	7.67E-04	(B) TP53 wild in GC
RPL26	17p13.1	12.42	12.75	0.85	0.74	-0.33	4.16E-05	7.73E-04	(B) TP53 wild in GC
GZMA	5q11.2	6.37	7.03	1.67	1.51	-0.66	4.24E-05	7.81E-04	(B) TP53 wild in GC
MTMR10	15q13.3	9.61	9.87	0.63	0.61	-0.26	4.27E-05	7.86E-04	(B) TP53 wild in GC
TMEM161	5q14.3	7.62	7.87	0.65	0.52	-0.25	4.33E-05	7.96E-04	(B) TP53 wild in GC
FBXL17	5q21.3	9.04	9.27	0.57	0.55	-0.23	4.34E-05	7.96E-04	(B) TP53 wild in GC
BBS12	4q27	6.25	6.52	0.64	0.66	-0.27	4.36E-05	7.96E-04	(B) TP53 wild in GC
THUMPD1	16p12.3	9.95	10.12	0.41	0.42	-0.17	4.43E-05	8.07E-04	(B) TP53 wild in GC
SLK	10q24.33-q	10.95	11.22	0.66	0.68	-0.27	4.45E-05	8.10E-04	(B) TP53 wild in GC
CD101	1p13.1	5.42	5.83	0.98	1.02	-0.41	4.46E-05	8.11E-04	(B) TP53 wild in GC

ZNF197	3p21.31	8.22	8.41	0.49	0.45	-0.19	4.51E-05	8.17E-04	(B) TP53 wild in GC
PLK2	5q11.2	9.11	9.54	1.01	1.11	-0.43	4.67E-05	8.38E-04	(B) TP53 wild in GC
EOGT	3p14.1	9.02	9.3	0.66	0.74	-0.29	4.67E-05	8.38E-04	(B) TP53 wild in GC
UBE2G2	21q22.3	9.78	9.97	0.49	0.43	-0.19	4.70E-05	8.41E-04	(B) TP53 wild in GC
GIMAP6	7q36.1	8.17	8.6	0.97	1.15	-0.43	4.71E-05	8.41E-04	(B) TP53 wild in GC
TUBGCP2	10q26.3	10.4	10.56	0.4	0.37	-0.16	4.72E-05	8.41E-04	(B) TP53 wild in GC
CDC14B	9q22.32-q2	8.91	9.16	0.65	0.6	-0.26	4.74E-05	8.43E-04	(B) TP53 wild in GC
TNFRSF10B	8p21.3	10.41	10.71	0.78	0.69	-0.3	4.76E-05	8.45E-04	(B) TP53 wild in GC
MTMR6	13q12.13	9.64	9.86	0.56	0.48	-0.22	5.09E-05	8.91E-04	(B) TP53 wild in GC
EDEM1	3p26.1	10.14	10.39	0.62	0.6	-0.25	5.17E-05	9.00E-04	(B) TP53 wild in GC
GNG2	14q22.1	8.28	8.67	0.97	1	-0.4	5.27E-05	9.14E-04	(B) TP53 wild in GC
ZNF654	3p11.1	8.19	8.4	0.52	0.5	-0.21	5.28E-05	9.14E-04	(B) TP53 wild in GC
KIAA0753	17p13.1	8.35	8.56	0.5	0.54	-0.21	5.30E-05	9.15E-04	(B) TP53 wild in GC
C3ORF38	3p11.1	8.73	8.89	0.42	0.38	-0.16	5.54E-05	9.48E-04	(B) TP53 wild in GC
SREK1IP1	5q12.3	9.71	9.98	0.73	0.57	-0.27	5.61E-05	9.57E-04	(B) TP53 wild in GC
LCP2	5q35.1	8.73	9.15	0.99	1.1	-0.42	5.65E-05	9.62E-04	(B) TP53 wild in GC

ZNF204P	6p22.1	6.14	6.75	1.69	1.21	-0.61	5.68E-05	9.66E-04	(B) TP53 wild in GC
NFKB1	4q24	10.43	10.61	0.47	0.45	-0.19	5.70E-05	9.68E-04	(B) TP53 wild in GC
DCP1B	12p13.33	8.55	8.81	0.64	0.64	-0.26	5.92E-05	9.99E-04	(B) TP53 wild in GC
IRF2	4q35.1	9.89	10.08	0.45	0.46	-0.18	5.94E-05	9.99E-04	(B) TP53 wild in GC
COPS2	15q21.1	10.25	10.42	0.4	0.44	-0.17	5.94E-05	9.99E-04	(B) TP53 wild in GC
STN1	10q24.33	8.9	9.13	0.58	0.57	-0.23	5.94E-05	9.99E-04	(B) TP53 wild in GC
MIAT	22q12.1	6.79	7.3	1.21	1.3	-0.5	6.01E-05	1.01E-03	(B) TP53 wild in GC
ALPK1	4q25	8.37	8.7	0.87	0.76	-0.33	6.06E-05	1.01E-03	(B) TP53 wild in GC
SYNJ2BP	14q24.2	10.2	10.4	0.49	0.51	-0.2	6.16E-05	1.03E-03	(B) TP53 wild in GC
SH2D1A	Xq25	5.1	5.74	1.58	1.59	-0.64	6.16E-05	1.03E-03	(B) TP53 wild in GC
WDR36	5q22.1	9.82	10.03	0.55	0.52	-0.22	6.16E-05	1.03E-03	(B) TP53 wild in GC
WDR48	3p22.2	9.45	9.66	0.54	0.46	-0.2	6.19E-05	1.03E-03	(B) TP53 wild in GC
STAT4	2q32.2-q32	5.57	6.07	1.23	1.28	-0.5	6.33E-05	1.05E-03	(B) TP53 wild in GC
FOXJ2	12p13.31	9.5	9.72	0.57	0.5	-0.22	6.34E-05	1.05E-03	(B) TP53 wild in GC
GRAP2	22q13.1	5.09	5.69	1.51	1.48	-0.6	6.35E-05	1.05E-03	(B) TP53 wild in GC
UHRF2	9p24.1	9.12	9.39	0.71	0.6	-0.27	6.37E-05	1.05E-03	(B) TP53 wild in GC

ZAP70	2q11.2	6.27	6.88	1.55	1.5	-0.61	6.56E-05	1.08E-03	(B) TP53 wild in GC
DTWD2	5q23.1	7.84	8.09	0.64	0.61	-0.25	6.72E-05	1.09E-03	(B) TP53 wild in GC
PLEKHA2	8p11.22	9.76	10.05	0.7	0.72	-0.28	6.82E-05	1.11E-03	(B) TP53 wild in GC
SLAMF6	1q23.2-q23	6.51	7.13	1.51	1.59	-0.62	6.84E-05	1.11E-03	(B) TP53 wild in GC
MAT2B	5q34	10.32	10.51	0.5	0.49	-0.2	6.97E-05	1.12E-03	(B) TP53 wild in GC
ZNF655	7q22.1	9.12	9.61	1.25	1.19	-0.49	7.13E-05	1.15E-03	(B) TP53 wild in GC
MS4A7	11q12.2	8.15	8.66	1.19	1.35	-0.5	7.15E-05	1.15E-03	(B) TP53 wild in GC
CRBN	3p26.2	8.8	8.99	0.48	0.51	-0.2	7.25E-05	1.16E-03	(B) TP53 wild in GC
SH3BGRL	Xq21.1	10.99	11.38	0.95	1.02	-0.39	7.26E-05	1.16E-03	(B) TP53 wild in GC
TCAIM	3p21.31	9.09	9.27	0.47	0.45	-0.18	7.28E-05	1.16E-03	(B) TP53 wild in GC
ARHGAP15	2q22.2-q22	6.22	6.74	1.29	1.35	-0.52	7.60E-05	1.20E-03	(B) TP53 wild in GC
SHOC2	10q25.2	10.02	10.18	0.42	0.4	-0.16	7.62E-05	1.20E-03	(B) TP53 wild in GC
NIN	14q22.1	9.24	9.6	0.88	0.96	-0.36	7.63E-05	1.20E-03	(B) TP53 wild in GC
SERPINB8	18q22.1	8.44	8.78	0.91	0.81	-0.34	7.67E-05	1.21E-03	(B) TP53 wild in GC
ZNF25	10p11.21	7.32	7.64	0.75	0.88	-0.32	7.75E-05	1.22E-03	(B) TP53 wild in GC
COX18	4q13.3	8.13	8.34	0.53	0.5	-0.21	7.93E-05	1.24E-03	(B) TP53 wild in GC

SLA2	20q11.23	5.64	6.18	1.37	1.38	-0.55	7.96E-05	1.24E-03	(B) TP53 wild in GC
DCP2	5q22.2	9.32	9.54	0.57	0.55	-0.22	8.27E-05	1.27E-03	(B) TP53 wild in GC
PTPRC	1q31.3-q32	9.6	10.18	1.44	1.48	-0.57	8.40E-05	1.29E-03	(B) TP53 wild in GC
TXNIP	1q21.1	12.69	13.13	1.08	1.16	-0.44	8.41E-05	1.29E-03	(B) TP53 wild in GC
CCL5	17q12	9.07	9.67	1.49	1.58	-0.6	8.55E-05	1.31E-03	(B) TP53 wild in GC
ERAP1	5q15	10.9	11.2	0.8	0.7	-0.3	8.88E-05	1.35E-03	(B) TP53 wild in GC
ZBTB1	14q23.3	9.55	9.72	0.44	0.42	-0.17	9.05E-05	1.37E-03	(B) TP53 wild in GC
CD6	11q12.2	7.22	7.73	1.27	1.33	-0.51	9.16E-05	1.38E-03	(B) TP53 wild in GC
MIR155HG	-	4.46	4.98	1.3	1.34	-0.52	9.30E-05	1.40E-03	(B) TP53 wild in GC
S1PR4	19p13.3	5.51	6.02	1.25	1.4	-0.51	9.48E-05	1.42E-03	(B) TP53 wild in GC
DOCK10	2q36.2	7.75	8.26	1.28	1.33	-0.51	9.48E-05	1.42E-03	(B) TP53 wild in GC
ZDHHC3	3p21.31	10.91	11.1	0.47	0.49	-0.19	9.60E-05	1.44E-03	(B) TP53 wild in GC
CDC37L1	9p24.1	7.51	7.74	0.6	0.56	-0.23	9.64E-05	1.44E-03	(B) TP53 wild in GC
CYTIP	2q24.1	7.71	8.21	1.26	1.28	-0.5	9.72E-05	1.45E-03	(B) TP53 wild in GC
CD226	18q22.2	4.23	4.81	1.53	1.42	-0.58	9.75E-05	1.45E-03	(B) TP53 wild in GC
IL10RA	11q23.3	8.5	9.01	1.31	1.31	-0.51	9.84E-05	1.46E-03	(B) TP53 wild in GC

FAM169A	5q13.3	6.74	7.28	1.43	1.28	-0.53	9.93E-05	1.47E-03	(B) TP53 wild in GC
PAXIP1-AS	7q36.2	6.24	6.56	0.83	0.75	-0.31	9.99E-05	1.47E-03	(B) TP53 wild in GC
SGCB	4q12	9.78	10.05	0.68	0.72	-0.27	1.00E-04	1.47E-03	(B) TP53 wild in GC
CD3D	11q23.3	6.51	7.08	1.47	1.45	-0.57	1.01E-04	1.49E-03	(B) TP53 wild in GC
GIMAP5	7q36.1	7.48	7.91	1.05	1.19	-0.43	1.02E-04	1.49E-03	(B) TP53 wild in GC
C1RL	12p13.31	9.48	9.76	0.74	0.69	-0.28	1.04E-04	1.52E-03	(B) TP53 wild in GC
MAP4K1	19q13.2	6.44	7.07	1.7	1.53	-0.63	1.04E-04	1.52E-03	(B) TP53 wild in GC
RGS10	10q26.11	8.46	8.78	0.83	0.78	-0.31	1.05E-04	1.53E-03	(B) TP53 wild in GC
CCR5	3p21.31	6.66	7.19	1.34	1.44	-0.54	1.05E-04	1.53E-03	(B) TP53 wild in GC
IKZF1	7p12.2	8.18	8.73	1.37	1.49	-0.55	1.06E-04	1.54E-03	(B) TP53 wild in GC
PPP1R16B	20q11.23	8	8.51	1.26	1.42	-0.52	1.09E-04	1.58E-03	(B) TP53 wild in GC
ST8SIA1	12p12.1	5.16	5.69	1.24	1.49	-0.52	1.09E-04	1.58E-03	(B) TP53 wild in GC
CRK	17p13.3	10.09	10.24	0.39	0.35	-0.15	1.09E-04	1.58E-03	(B) TP53 wild in GC
UIMC1	5q35.2	8.26	8.41	0.42	0.38	-0.16	1.12E-04	1.61E-03	(B) TP53 wild in GC
IFI16	1q23.1	10.51	10.92	1.03	1.07	-0.41	1.12E-04	1.61E-03	(B) TP53 wild in GC
EVL	14q32.2	9.17	9.56	0.97	1.04	-0.39	1.15E-04	1.64E-03	(B) TP53 wild in GC

RABGAP1L	1q25.1	8.69	8.93	0.6	0.63	-0.24	1.16E-04	1.65E-03	(B) TP53 wild in GC
C5ORF24	5q31.1	10.3	10.48	0.46	0.48	-0.18	1.16E-04	1.66E-03	(B) TP53 wild in GC
YBEY	21q22.3	6.69	7	0.84	0.81	-0.32	1.22E-04	1.72E-03	(B) TP53 wild in GC
KIAA1109	4q27	10.34	10.59	0.63	0.67	-0.25	1.22E-04	1.72E-03	(B) TP53 wild in GC
CXCR4	2q22.1	10.09	10.55	1.21	1.21	-0.47	1.22E-04	1.73E-03	(B) TP53 wild in GC
TRAK1	3p22.1	11.28	11.55	0.72	0.71	-0.28	1.24E-04	1.74E-03	(B) TP53 wild in GC
CLK4	5q35.3	8.13	8.37	0.67	0.6	-0.25	1.24E-04	1.74E-03	(B) TP53 wild in GC
CCSER2	10q23.1	10.03	10.27	0.6	0.71	-0.25	1.25E-04	1.75E-03	(B) TP53 wild in GC
DHX29	5q11.2	9.46	9.63	0.46	0.44	-0.17	1.25E-04	1.75E-03	(B) TP53 wild in GC
SLC35E3	12q15	7.49	7.75	0.59	0.78	-0.26	1.25E-04	1.75E-03	(B) TP53 wild in GC
CD247	1q24.2	5.75	6.25	1.31	1.32	-0.51	1.26E-04	1.76E-03	(B) TP53 wild in GC
ST8SIA4	5q21.1	7.68	8.05	0.94	1.01	-0.37	1.30E-04	1.81E-03	(B) TP53 wild in GC
SLFN5	17q12	9.2	9.58	1.01	0.99	-0.38	1.31E-04	1.83E-03	(B) TP53 wild in GC
VPS53	17p13.3	10.13	10.31	0.46	0.48	-0.18	1.32E-04	1.83E-03	(B) TP53 wild in GC
GIMAP4	7q36.1	8.68	9.07	0.97	1.1	-0.39	1.32E-04	1.83E-03	(B) TP53 wild in GC
TLE4	9q21.31	8.47	8.85	1.01	0.94	-0.38	1.33E-04	1.84E-03	(B) TP53 wild in GC

ITM2A	Xq21.1	7.64	8.18	1.39	1.45	-0.54	1.36E-04	1.88E-03	(B) TP53 wild in GC
FAM13A	4q22.1	8.67	9.08	1.11	1.06	-0.42	1.42E-04	1.95E-03	(B) TP53 wild in GC
TSPYL1	6q22.1	10.57	10.76	0.53	0.46	-0.19	1.42E-04	1.95E-03	(B) TP53 wild in GC
STAT2	12q13.3	10.56	10.79	0.6	0.57	-0.22	1.43E-04	1.96E-03	(B) TP53 wild in GC
LYSMD3	5q14.3	9.06	9.25	0.51	0.46	-0.19	1.46E-04	1.99E-03	(B) TP53 wild in GC
CYTH1	17q25.3	9.74	9.93	0.51	0.51	-0.19	1.47E-04	2.00E-03	(B) TP53 wild in GC
CCDC171	9p22.3	5.07	5.45	1.02	0.98	-0.38	1.47E-04	2.00E-03	(B) TP53 wild in GC
NDEL1	17p13.1	9.53	9.69	0.44	0.42	-0.16	1.48E-04	2.01E-03	(B) TP53 wild in GC
EVI2B	17q11.2	8.96	9.48	1.27	1.44	-0.51	1.49E-04	2.01E-03	(B) TP53 wild in GC
GIMAP7	7q36.1	7.32	7.76	1.06	1.28	-0.44	1.52E-04	2.04E-03	(B) TP53 wild in GC
G3BP2	4q21.1	11.46	11.63	0.46	0.45	-0.17	1.53E-04	2.06E-03	(B) TP53 wild in GC
EIF4A2	3q27.3	12.4	12.62	0.63	0.52	-0.22	1.55E-04	2.07E-03	(B) TP53 wild in GC
TRAPPC6B	14q21.1	8.81	8.97	0.45	0.39	-0.16	1.55E-04	2.07E-03	(B) TP53 wild in GC
PPWD1	5q12.3	8.23	8.4	0.48	0.43	-0.17	1.55E-04	2.07E-03	(B) TP53 wild in GC
PARP11	12p13.32	7.36	7.67	0.85	0.74	-0.31	1.57E-04	2.10E-03	(B) TP53 wild in GC
MYO1F	19p13.2	8.24	8.65	1.04	1.13	-0.41	1.58E-04	2.10E-03	(B) TP53 wild in GC

EVI2A	17q11.2	7.09	7.55	1.17	1.26	-0.46	1.62E-04	2.15E-03	(B) TP53 wild in GC
ZFP14	19q13.12	6.96	7.27	0.86	0.73	-0.31	1.64E-04	2.16E-03	(B) TP53 wild in GC
FYCO1	3p21.31	10.25	10.56	0.75	0.88	-0.3	1.64E-04	2.16E-03	(B) TP53 wild in GC
ARL14EP	11p14.1	8.33	8.52	0.48	0.48	-0.18	1.65E-04	2.17E-03	(B) TP53 wild in GC
TRMO	9q22.33	7.45	7.62	0.47	0.42	-0.17	1.65E-04	2.17E-03	(B) TP53 wild in GC
RPL15	3p24.2	13.65	13.86	0.53	0.53	-0.2	1.65E-04	2.17E-03	(B) TP53 wild in GC
IL6ST	5q11.2	9.74	10.17	1.06	1.24	-0.43	1.69E-04	2.20E-03	(B) TP53 wild in GC
SMARCAD 1	4q22.3	9.67	9.87	0.55	0.54	-0.21	1.71E-04	2.22E-03	(B) TP53 wild in GC
CHURC1 1	4q23.3	10.15	10.33	0.49	0.51	-0.19	1.71E-04	2.22E-03	(B) TP53 wild in GC
DIMT1	5q12.1	8.84	9.01	0.44	0.46	-0.17	1.72E-04	2.22E-03	(B) TP53 wild in GC
SAP30L	5q33.2	9.39	9.58	0.53	0.47	-0.19	1.73E-04	2.23E-03	(B) TP53 wild in GC
GIMAP8	7q36.1	7.4	7.79	0.95	1.13	-0.39	1.76E-04	2.25E-03	(B) TP53 wild in GC
PML 1	5q24.1	11.38	11.63	0.66	0.67	-0.25	1.78E-04	2.27E-03	(B) TP53 wild in GC
SLC30A9	4p13	10.14	10.31	0.46	0.42	-0.17	1.82E-04	2.31E-03	(B) TP53 wild in GC
VCL	10q22.2	12.21	12.51	0.77	0.86	-0.3	1.83E-04	2.31E-03	(B) TP53 wild in GC
TESPA1	12q13.2	4.27	4.91	1.67	1.71	-0.63	1.83E-04	2.31E-03	(B) TP53 wild in GC

CTSO	4q32.1	9.11	9.47	0.98	0.9	-0.36	1.84E-04	2.32E-03	(B) TP53 wild in GC
TNFSF9	19p13.3	5.84	6.57	1.82	2.07	-0.72	1.85E-04	2.34E-03	(B) TP53 wild in GC
KLRA1P	12p13.2	4.19	4.63	1.17	1.16	-0.44	1.86E-04	2.35E-03	(B) TP53 wild in GC
RNF125	18q12.1	8.02	8.41	1.08	0.98	-0.39	1.88E-04	2.37E-03	(B) TP53 wild in GC
KDM3B	5q31.2	11.08	11.24	0.43	0.43	-0.16	1.90E-04	2.39E-03	(B) TP53 wild in GC
BDP1	5q13.2	10	10.22	0.6	0.55	-0.22	1.94E-04	2.42E-03	(B) TP53 wild in GC
RUNX1	21q22.12	11.21	11.45	0.61	0.73	-0.25	1.99E-04	2.47E-03	(B) TP53 wild in GC
BIN2	12q13.13	7.35	7.76	1.04	1.17	-0.41	2.00E-04	2.48E-03	(B) TP53 wild in GC
CHRFAM7A	15q13.2	5.15	5.81	1.8	1.72	-0.66	2.01E-04	2.49E-03	(B) TP53 wild in GC
HIF1AN	10q24.31	9.77	9.95	0.49	0.47	-0.18	2.01E-04	2.49E-03	(B) TP53 wild in GC
PVRIG	7q22.1	6.28	6.7	1.08	1.15	-0.41	2.02E-04	2.49E-03	(B) TP53 wild in GC
ARHGAP9	12q13.3	7.24	7.7	1.2	1.27	-0.46	2.03E-04	2.50E-03	(B) TP53 wild in GC
GPR18	13q32.3	3.88	4.38	1.23	1.5	-0.5	2.07E-04	2.54E-03	(B) TP53 wild in GC
ENTPD1	10q24.1	10.39	10.65	0.65	0.77	-0.26	2.07E-04	2.54E-03	(B) TP53 wild in GC
NR2F2	15q26.2	10.67	11	0.86	0.91	-0.33	2.14E-04	2.60E-03	(B) TP53 wild in GC
PPM1A	14q23.1	10.16	10.32	0.43	0.45	-0.16	2.14E-04	2.60E-03	(B) TP53 wild in GC

MAST4	5q12.3	8.93	9.28	0.92	1	-0.35	2.15E-04	2.60E-03	(B) TP53 wild in GC
FNBP1	9q34.11	9.81	10.26	1.14	1.34	-0.45	2.15E-04	2.61E-03	(B) TP53 wild in GC
MIER1	1p31.3	10.3	10.46	0.41	0.41	-0.15	2.22E-04	2.67E-03	(B) TP53 wild in GC
DAB2IP	9q33.2	11.05	11.32	0.76	0.69	-0.27	2.23E-04	2.67E-03	(B) TP53 wild in GC
PAM	5q21.1	10.85	11.16	0.81	0.89	-0.31	2.27E-04	2.72E-03	(B) TP53 wild in GC
C14ORF28	14q21.2	6.62	6.87	0.67	0.68	-0.25	2.28E-04	2.72E-03	(B) TP53 wild in GC
ARAP2	4p14	8.77	9.11	0.96	0.83	-0.34	2.34E-04	2.79E-03	(B) TP53 wild in GC
SFMBT2	10p14	5.53	6.04	1.33	1.46	-0.51	2.40E-04	2.84E-03	(B) TP53 wild in GC
RPP14	3p14.3	8.97	9.11	0.39	0.38	-0.14	2.41E-04	2.84E-03	(B) TP53 wild in GC
MAN2A1	5q21.3	9.66	9.94	0.76	0.75	-0.28	2.41E-04	2.84E-03	(B) TP53 wild in GC
ZNF624 1	7p11.2	6.44	6.66	0.62	0.58	-0.22	2.41E-04	2.84E-03	(B) TP53 wild in GC
RASGRP1	15q14	6.99	7.49	1.42	1.22	-0.49	2.41E-04	2.85E-03	(B) TP53 wild in GC
ARHGAP1	11p11.2	11.8	11.98	0.47	0.5	-0.18	2.44E-04	2.86E-03	(B) TP53 wild in GC
ATG12	5q22.3	8.85	9.01	0.46	0.43	-0.16	2.46E-04	2.89E-03	(B) TP53 wild in GC
ITGAL	16p11.2	8.42	8.95	1.43	1.49	-0.54	2.47E-04	2.90E-03	(B) TP53 wild in GC
LSM11	5q33.3	7.47	7.68	0.55	0.59	-0.21	2.48E-04	2.91E-03	(B) TP53 wild in GC

GM2A	5q33.1	10.63	10.9	0.76	0.69	-0.27	2.50E-04	2.93E-03	(B) TP53 wild in GC
JKAMP	14q23.1	9.57	9.73	0.46	0.44	-0.16	2.54E-04	2.97E-03	(B) TP53 wild in GC
FAM78A	9q34.13	7.3	7.68	1.02	1.03	-0.38	2.55E-04	2.97E-03	(B) TP53 wild in GC
NCKAP1L	12q13.13-q	8.22	8.74	1.48	1.38	-0.53	2.57E-04	2.99E-03	(B) TP53 wild in GC
MIER3	5q11.2	9.11	9.31	0.56	0.54	-0.2	2.57E-04	2.99E-03	(B) TP53 wild in GC
TVP23B	17p11.2	9.09	9.31	0.63	0.56	-0.22	2.60E-04	3.02E-03	(B) TP53 wild in GC
SLF1	5q15	6.96	7.18	0.62	0.58	-0.22	2.62E-04	3.04E-03	(B) TP53 wild in GC
RNF144B	6p22.3	8	8.39	1.04	1.11	-0.39	2.63E-04	3.05E-03	(B) TP53 wild in GC
TMX3	18q22.1	9.51	9.73	0.62	0.56	-0.22	2.72E-04	3.13E-03	(B) TP53 wild in GC
GMFG	19q13.2	7.2	7.55	0.93	1.01	-0.35	2.77E-04	3.17E-03	(B) TP53 wild in GC
TRIAP1	12q24.31	8.63	8.81	0.47	0.54	-0.18	2.78E-04	3.18E-03	(B) TP53 wild in GC
FOXO1	13q14.11	9.66	9.98	0.87	0.84	-0.31	2.80E-04	3.20E-03	(B) TP53 wild in GC
PIK3CG	7q22.3	6.7	7.27	1.52	1.59	-0.56	2.80E-04	3.20E-03	(B) TP53 wild in GC
TNFSF13B	13q33.3	6.38	6.84	1.29	1.25	-0.46	2.81E-04	3.20E-03	(B) TP53 wild in GC
TAGAP	6q25.3	7.08	7.59	1.4	1.46	-0.52	2.87E-04	3.26E-03	(B) TP53 wild in GC
RPL13A	19q13.33	12.05	12.3	0.72	0.65	-0.25	2.93E-04	3.31E-03	(B) TP53 wild in GC

CFAP36	2p16.1	8.84	9.04	0.57	0.52	-0.2	2.96E-04	3.34E-03	(B) TP53 wild in GC
CDK17	12q23.1	9.15	9.34	0.48	0.55	-0.19	2.98E-04	3.36E-03	(B) TP53 wild in GC
NRIP1	21q11.2-q2	10.1	10.33	0.68	0.58	-0.23	2.99E-04	3.36E-03	(B) TP53 wild in GC
DUT	15q21.1	9.5	9.7	0.56	0.53	-0.2	2.99E-04	3.36E-03	(B) TP53 wild in GC
RPS3A	4q31.3	11.31	11.56	0.73	0.69	-0.26	3.00E-04	3.36E-03	(B) TP53 wild in GC
C1ORF54	1q21.2	6.19	6.52	0.88	0.94	-0.33	3.01E-04	3.36E-03	(B) TP53 wild in GC
BACH1	21q21.3	9.87	10.08	0.57	0.61	-0.21	3.01E-04	3.36E-03	(B) TP53 wild in GC
ZNF658	9q21.11	4.97	5.34	1.02	0.99	-0.37	3.01E-04	3.36E-03	(B) TP53 wild in GC
CEP85L	6q22.31	5.25	5.69	1.19	1.2	-0.43	3.02E-04	3.36E-03	(B) TP53 wild in GC
IL7R	5p13.2	6.88	7.43	1.47	1.57	-0.55	3.02E-04	3.36E-03	(B) TP53 wild in GC
MS4A6A	11q12.2	8.59	9.05	1.23	1.32	-0.46	3.03E-04	3.37E-03	(B) TP53 wild in GC
CYTH4	22q13.1	7.58	8	1.13	1.2	-0.42	3.06E-04	3.39E-03	(B) TP53 wild in GC
RASSF2	20p13	8.71	9.15	1.16	1.3	-0.44	3.06E-04	3.39E-03	(B) TP53 wild in GC
ZNF35	3p21.31	7.47	7.63	0.46	0.41	-0.16	3.07E-04	3.40E-03	(B) TP53 wild in GC
GFM2	5q13.3	8.94	9.12	0.51	0.53	-0.19	3.15E-04	3.47E-03	(B) TP53 wild in GC
PCNX1	14q24.2	10.29	10.51	0.56	0.66	-0.22	3.16E-04	3.48E-03	(B) TP53 wild in GC

SRSF5	14q24.1	11.01	11.17	0.44	0.45	-0.16	3.18E-04	3.49E-03	(B) TP53 wild in GC
RPL36AL	14q21.3	11.27	11.49	0.64	0.58	-0.22	3.18E-04	3.49E-03	(B) TP53 wild in GC
TNPO1	5q13.2	11.24	11.4	0.46	0.44	-0.16	3.23E-04	3.52E-03	(B) TP53 wild in GC
TANC1	2q24.2	10.01	10.22	0.57	0.6	-0.21	3.23E-04	3.52E-03	(B) TP53 wild in GC
DOCK2	5q35.1	8.07	8.6	1.51	1.43	-0.53	3.25E-04	3.53E-03	(B) TP53 wild in GC
WIPF1	2q31.1	10.2	10.57	0.98	1.09	-0.37	3.26E-04	3.53E-03	(B) TP53 wild in GC
ARL15	5q11.2	7.68	7.9	0.6	0.63	-0.22	3.28E-04	3.54E-03	(B) TP53 wild in GC
CYFIP1	15q11.2	11.46	11.62	0.44	0.4	-0.15	3.37E-04	3.63E-03	(B) TP53 wild in GC
ZNF596	8p23.3	5.16	5.5	0.95	0.92	-0.34	3.39E-04	3.64E-03	(B) TP53 wild in GC
FBXW2	9q33.2	10.47	10.62	0.43	0.41	-0.15	3.42E-04	3.68E-03	(B) TP53 wild in GC
LNPEP	5q15	8.31	8.63	0.87	0.91	-0.32	3.44E-04	3.69E-03	(B) TP53 wild in GC
ANKRD44	2q33.1	6.38	6.89	1.42	1.4	-0.51	3.44E-04	3.69E-03	(B) TP53 wild in GC
PRF1	10q22.1	7	7.49	1.39	1.32	-0.49	3.46E-04	3.70E-03	(B) TP53 wild in GC
YIPF5	5q31.3	9.77	9.93	0.45	0.47	-0.16	3.48E-04	3.71E-03	(B) TP53 wild in GC
LPXN	11q12.1	8.11	8.45	0.96	0.94	-0.34	3.49E-04	3.72E-03	(B) TP53 wild in GC
BLOC1S6	15q21.1	9.91	10.06	0.39	0.43	-0.15	3.51E-04	3.73E-03	(B) TP53 wild in GC

RHOA	3p21.31	13.3	13.44	0.37	0.4	-0.14	3.52E-04	3.74E-03	(B) TP53 wild in GC
SREBF1	17p11.2	11.11	11.4	0.83	0.83	-0.3	3.53E-04	3.74E-03	(B) TP53 wild in GC
SETX	9q34.13	11.03	11.24	0.56	0.58	-0.2	3.55E-04	3.76E-03	(B) TP53 wild in GC
1-Mar	4q32.2-q32	5.83	6.29	1.26	1.3	-0.46	3.64E-04	3.84E-03	(B) TP53 wild in GC
PDE4B	1p31.3	8.33	8.77	1.19	1.25	-0.43	3.71E-04	3.90E-03	(B) TP53 wild in GC
MARF1	16p13.11	10.76	10.97	0.54	0.61	-0.2	3.73E-04	3.92E-03	(B) TP53 wild in GC
S1PR2	19p13.2	7.34	7.64	0.91	0.79	-0.31	3.75E-04	3.93E-03	(B) TP53 wild in GC
HAPLN3	15q26.1	7.91	8.32	1.13	1.17	-0.41	3.75E-04	3.93E-03	(B) TP53 wild in GC
CBR4	4q32.3	8.3	8.51	0.61	0.59	-0.21	3.76E-04	3.93E-03	(B) TP53 wild in GC
GGTA1P	9q33.2	5.78	6.26	1.29	1.44	-0.48	3.79E-04	3.96E-03	(B) TP53 wild in GC
LIMD1	3p21.31	9.81	9.99	0.51	0.52	-0.18	3.82E-04	3.98E-03	(B) TP53 wild in GC
PHF21A	11p11.2	9.26	9.48	0.64	0.58	-0.22	3.82E-04	3.98E-03	(B) TP53 wild in GC
ACAP1	17p13.1	6.4	6.91	1.41	1.48	-0.51	3.83E-04	3.98E-03	(B) TP53 wild in GC
JAML	11q23.3	6.86	7.31	1.17	1.36	-0.45	3.83E-04	3.98E-03	(B) TP53 wild in GC
TRAF3IP3	1q32.2	6.06	6.5	1.17	1.34	-0.44	3.85E-04	3.99E-03	(B) TP53 wild in GC
MS4A14	11q12.2	4.75	5.19	1.18	1.33	-0.44	3.88E-04	4.01E-03	(B) TP53 wild in GC

VPS13C	15q22.2	10.7	10.94	0.66	0.71	-0.24	3.88E-04	4.01E-03	(B) TP53 wild in GC
TMEM64	8q21.3	8.85	9.18	0.92	0.93	-0.33	3.91E-04	4.03E-03	(B) TP53 wild in GC
CHSY1	15q26.3	9.76	9.97	0.56	0.61	-0.21	3.97E-04	4.07E-03	(B) TP53 wild in GC
CD47	3q13.12	11.08	11.3	0.6	0.66	-0.22	3.98E-04	4.07E-03	(B) TP53 wild in GC
TNFSF14	19p13.3	3.16	3.67	1.39	1.48	-0.51	3.98E-04	4.07E-03	(B) TP53 wild in GC
HCLS1	3q13.33	8.62	9.02	1.09	1.18	-0.4	4.01E-04	4.09E-03	(B) TP53 wild in GC
NPM1	5q35.1	11.79	12.03	0.66	0.66	-0.23	4.02E-04	4.09E-03	(B) TP53 wild in GC
RBM5	3p21.31	10.2	10.38	0.48	0.53	-0.18	4.12E-04	4.16E-03	(B) TP53 wild in GC
CARD8	19q13.33	8.63	8.85	0.67	0.61	-0.23	4.12E-04	4.16E-03	(B) TP53 wild in GC
FBXW11	5q35.1	9.76	9.94	0.5	0.53	-0.18	4.17E-04	4.19E-03	(B) TP53 wild in GC
CTSW	11q13.1	5.75	6.41	1.93	1.81	-0.66	4.17E-04	4.19E-03	(B) TP53 wild in GC
SEPTIN6	Xq24	9.45	9.81	1.07	1	-0.37	4.19E-04	4.20E-03	(B) TP53 wild in GC
USP25	21q21.1	9.45	9.64	0.55	0.5	-0.19	4.19E-04	4.20E-03	(B) TP53 wild in GC
MED7	5q33.3	7.4	7.56	0.48	0.42	-0.16	4.23E-04	4.22E-03	(B) TP53 wild in GC
TMF1	3p14.1	10.24	10.43	0.53	0.53	-0.19	4.23E-04	4.22E-03	(B) TP53 wild in GC
ATG10	5q14.1-q14	6.16	6.36	0.58	0.54	-0.2	4.26E-04	4.24E-03	(B) TP53 wild in GC

CD209	19p13.2	7.24	7.76	1.4	1.52	-0.51	4.29E-04	4.26E-03	(B) TP53 wild in GC
RANBP3	19p13.3	10.16	10.3	0.42	0.38	-0.14	4.33E-04	4.29E-03	(B) TP53 wild in GC
GPX8	5q11.2	8.5	8.87	0.99	1.17	-0.38	4.38E-04	4.34E-03	(B) TP53 wild in GC
PALLD	4q32.3	11.65	12.07	1.09	1.3	-0.42	4.53E-04	4.46E-03	(B) TP53 wild in GC
DOCK8	9p24.3	8.99	9.41	1.24	1.15	-0.42	4.56E-04	4.48E-03	(B) TP53 wild in GC
ARRDC4	15q26.2	9.02	9.37	0.97	1.04	-0.35	4.61E-04	4.51E-03	(B) TP53 wild in GC
GBP4	1p22.2	10.16	10.69	1.43	1.58	-0.52	4.62E-04	4.52E-03	(B) TP53 wild in GC
RCSD1	1q24.2	7.66	8.06	1.1	1.22	-0.4	4.62E-04	4.52E-03	(B) TP53 wild in GC
DCUN1D4	4q12	8.91	9.09	0.52	0.53	-0.18	4.63E-04	4.52E-03	(B) TP53 wild in GC
SCML4	6q21	5.19	5.77	1.65	1.67	-0.58	4.64E-04	4.52E-03	(B) TP53 wild in GC
GPR65	14q31.3	5.73	6.16	1.15	1.31	-0.43	4.64E-04	4.52E-03	(B) TP53 wild in GC
PIK3R3	1p34.1	9.12	9.37	0.68	0.82	-0.26	4.66E-04	4.53E-03	(B) TP53 wild in GC
LILRB1	19q13.42	6.4	6.86	1.28	1.37	-0.46	4.67E-04	4.55E-03	(B) TP53 wild in GC
RCL1	9p24.1	8.21	8.45	0.6	0.79	-0.24	4.68E-04	4.55E-03	(B) TP53 wild in GC
AFF1	4q21.3-q22	10.75	10.96	0.58	0.61	-0.21	4.70E-04	4.56E-03	(B) TP53 wild in GC
LURAP1L	9p23	8.48	8.83	1.08	0.94	-0.36	4.71E-04	4.56E-03	(B) TP53 wild in GC

SELPLG	12q24.11	8.56	8.94	1.1	1.1	-0.39	4.72E-04	4.56E-03	(B) TP53 wild in GC
CENPC	4q13.2	7.78	7.98	0.58	0.57	-0.2	4.74E-04	4.58E-03	(B) TP53 wild in GC
CYB5D1	17p13.1	8.36	8.55	0.54	0.56	-0.19	4.75E-04	4.58E-03	(B) TP53 wild in GC
IGIP	5q31.3	7.69	7.93	0.65	0.76	-0.24	4.75E-04	4.58E-03	(B) TP53 wild in GC
LMF1	16p13.3	7.43	7.76	0.98	0.87	-0.33	4.77E-04	4.60E-03	(B) TP53 wild in GC
CD48	1q23.3	7.69	8.19	1.36	1.54	-0.5	4.80E-04	4.62E-03	(B) TP53 wild in GC
ABRAXAS1	4q21.23	7.35	7.59	0.7	0.67	-0.24	4.86E-04	4.66E-03	(B) TP53 wild in GC
PTGER2	14q22.1	7.19	7.7	1.46	1.5	-0.52	4.91E-04	4.70E-03	(B) TP53 wild in GC
ZNF831	20q13.32	4.88	5.51	1.81	1.76	-0.62	5.00E-04	4.77E-03	(B) TP53 wild in GC
ANK1	8p11.21	5.9	6.55	1.7	2.06	-0.65	5.01E-04	4.77E-03	(B) TP53 wild in GC
TRAPPC10	21q22.3	10.34	10.5	0.48	0.43	-0.16	5.08E-04	4.82E-03	(B) TP53 wild in GC
PDCD1LG2	9p24.1	5.53	6.01	1.31	1.44	-0.48	5.10E-04	4.84E-03	(B) TP53 wild in GC
PRKCB	16p12.2-p1	7.89	8.46	1.52	1.78	-0.57	5.14E-04	4.87E-03	(B) TP53 wild in GC
MTREX	5q11.2	10.14	10.3	0.45	0.46	-0.16	5.15E-04	4.87E-03	(B) TP53 wild in GC
NKG7	19q13.41	7.03	7.59	1.59	1.65	-0.56	5.19E-04	4.91E-03	(B) TP53 wild in GC
CFH	1q31.3	9.92	10.36	1.17	1.4	-0.44	5.21E-04	4.92E-03	(B) TP53 wild in GC

PITPNC1	17q24.2	7.93	8.22	0.85	0.8	-0.29	5.23E-04	4.92E-03	(B) TP53 wild in GC
RORA	15q22.2	7.89	8.26	1.03	1.12	-0.37	5.26E-04	4.95E-03	(B) TP53 wild in GC
SACM1L	3p21.31	9.7	9.85	0.42	0.42	-0.15	5.38E-04	5.05E-03	(B) TP53 wild in GC
GNPNAT1	14q22.1	10.35	10.59	0.67	0.69	-0.23	5.39E-04	5.05E-03	(B) TP53 wild in GC
ATP11C	Xq27.1	8.65	8.91	0.76	0.7	-0.25	5.41E-04	5.06E-03	(B) TP53 wild in GC
TP53INP1	8q22.1	9.17	9.47	0.85	0.88	-0.3	5.42E-04	5.06E-03	(B) TP53 wild in GC
RPS6	9p22.1	14.02	14.26	0.72	0.68	-0.24	5.43E-04	5.06E-03	(B) TP53 wild in GC
RGS18	1q31.2	4.84	5.29	1.2	1.46	-0.45	5.53E-04	5.14E-03	(B) TP53 wild in GC
EPB41L4A-	5q22.1	7.42	7.7	0.83	0.78	-0.28	5.61E-04	5.19E-03	(B) TP53 wild in GC
PRKAR2B	7q22.3	7.3	7.8	1.3	1.62	-0.5	5.63E-04	5.21E-03	(B) TP53 wild in GC
IL2RB	22q12.3	8.8	9.23	1.25	1.26	-0.43	5.64E-04	5.21E-03	(B) TP53 wild in GC
LOC15476	17q35	4.57	4.96	1.19	1.07	-0.39	5.65E-04	5.22E-03	(B) TP53 wild in GC
GIMAP2	7q36.1	7.11	7.45	1	0.95	-0.34	5.73E-04	5.27E-03	(B) TP53 wild in GC
RIPOR2	6p22.3	6.48	7.01	1.46	1.66	-0.53	5.76E-04	5.29E-03	(B) TP53 wild in GC
MKNK2	19p13.3	11.46	11.67	0.62	0.58	-0.21	5.85E-04	5.35E-03	(B) TP53 wild in GC
TMC8	17q25.3	8.28	8.66	1.07	1.18	-0.38	5.87E-04	5.36E-03	(B) TP53 wild in GC

RNF14	5q31.3	9.32	9.46	0.39	0.43	-0.14	5.89E-04	5.37E-03	(B) TP53 wild in GC
CYBB Xp	21.1-p11	9.68	10.13	1.3	1.33	-0.45	5.92E-04	5.38E-03	(B) TP53 wild in GC
ATP8B4	15q21.2	5.43	5.84	1.14	1.24	-0.41	5.93E-04	5.39E-03	(B) TP53 wild in GC
ZBTB38	3q23	10.96	11.17	0.58	0.69	-0.22	6.01E-04	5.45E-03	(B) TP53 wild in GC
PXK 3p	14.3	7.86	8.1	0.71	0.65	-0.24	6.10E-04	5.51E-03	(B) TP53 wild in GC
ACTR8	3p21. 1	8.4	8.54	0.45	0.39	-0.15	6.14E-04	5.54E-03	(B) TP53 wild in GC
5-Mar	10q23.32-q	9.48	9.63	0.42	0.45	-0.15	6.15E-04	5.54E-03	(B) TP53 wild in GC
FYB1	5p13.1	9.2	9.62	1.16	1.29	-0.42	6.16E-04	5.54E-03	(B) TP53 wild in GC
GABPA	21q21.3	9.37	9.53	0.44	0.46	-0.15	6.19E-04	5.56E-03	(B) TP53 wild in GC
MAPK1IP1	14q22.3	11.44	11.57	0.4	0.34	-0.13	6.19E-04	5.56E-03	(B) TP53 wild in GC
UTP15	5q13.2	8.03	8.2	0.47	0.47	-0.16	6.20E-04	5.56E-03	(B) TP53 wild in GC
TLR7	Xp22.2	5.82	6.37	1.57	1.66	-0.55	6.22E-04	5.57E-03	(B) TP53 wild in GC
BBC3	19q13.32	7.93	8.26	0.92	0.99	-0.33	6.25E-04	5.59E-03	(B) TP53 wild in GC
RASAL3	19p13.12	7.45	7.86	1.17	1.26	-0.41	6.25E-04	5.59E-03	(B) TP53 wild in GC
CD4	12p13.31	9.73	10.12	1.14	1.16	-0.39	6.26E-04	5.59E-03	(B) TP53 wild in GC
MNT	17p13.3	9.71	9.87	0.46	0.49	-0.16	6.28E-04	5.60E-03	(B) TP53 wild in GC

HMG20A	15q24.3	9.38	9.51	0.39	0.38	-0.13	6.33E-04	5.63E-03	(B) TP53 wild in GC
COX10	17p12	8.56	8.72	0.49	0.47	-0.16	6.33E-04	5.63E-03	(B) TP53 wild in GC
MEIS1	2p14	8.02	8.46	1.21	1.4	-0.44	6.34E-04	5.63E-03	(B) TP53 wild in GC
PDE12	3p14.3	9.73	9.9	0.53	0.44	-0.17	6.37E-04	5.65E-03	(B) TP53 wild in GC
RNASE6	14q11.2	7.75	8.17	1.15	1.36	-0.42	6.44E-04	5.70E-03	(B) TP53 wild in GC
RPL10A	6p21.31	12.72	12.92	0.62	0.58	-0.21	6.45E-04	5.70E-03	(B) TP53 wild in GC
COX15	10q24.2	10.04	10.19	0.43	0.46	-0.15	6.47E-04	5.71E-03	(B) TP53 wild in GC
SMAD2	18q21.1	10.38	10.55	0.49	0.46	-0.16	6.47E-04	5.71E-03	(B) TP53 wild in GC
ACAD11	3q22.1	7.46	7.71	0.69	0.77	-0.25	6.48E-04	5.71E-03	(B) TP53 wild in GC
GLIPR2	9p13.3	8.04	8.4	0.98	1.13	-0.36	6.50E-04	5.72E-03	(B) TP53 wild in GC
TBC1D1	4p14	10.35	10.57	0.6	0.7	-0.22	6.53E-04	5.74E-03	(B) TP53 wild in GC
GLIPR1	12q21.2	9.03	9.42	0.98	1.32	-0.39	6.54E-04	5.74E-03	(B) TP53 wild in GC
ESR1	6q25.1-q25	4.25	4.75	1.38	1.58	-0.5	6.56E-04	5.76E-03	(B) TP53 wild in GC
DPYSL2	8p21.2	10.89	11.18	0.84	0.85	-0.29	6.58E-04	5.77E-03	(B) TP53 wild in GC
MCUB	4q25	7.77	8.07	0.92	0.8	-0.3	6.59E-04	5.78E-03	(B) TP53 wild in GC
THAP6	4q21.1	8.07	8.21	0.4	0.43	-0.14	6.60E-04	5.78E-03	(B) TP53 wild in GC

HERC1	15q22.31	10.17	10.39	0.61	0.65	-0.21	6.63E-04	5.81E-03	(B) TP53 wild in GC
PIK3R6	17p13.1	4.99	5.39	1.16	1.19	-0.4	6.66E-04	5.83E-03	(B) TP53 wild in GC
MRPL54	19p13.3	8.27	8.49	0.63	0.62	-0.21	6.71E-04	5.86E-03	(B) TP53 wild in GC
PARVA	11p15.3	10.57	10.83	0.68	0.84	-0.26	6.73E-04	5.87E-03	(B) TP53 wild in GC
UBE2D3	4q24	11.73	11.86	0.42	0.38	-0.14	6.76E-04	5.89E-03	(B) TP53 wild in GC
SIRPG	20p13	5.51	6.06	1.63	1.62	-0.55	6.77E-04	5.89E-03	(B) TP53 wild in GC
MEF2A	15q26.3	10.37	10.59	0.58	0.71	-0.22	6.81E-04	5.92E-03	(B) TP53 wild in GC
FAM114A2	5q33.2	8.27	8.42	0.46	0.46	-0.16	6.86E-04	5.93E-03	(B) TP53 wild in GC
MFAP3	5q33.2	9.56	9.72	0.44	0.46	-0.15	6.87E-04	5.94E-03	(B) TP53 wild in GC
CMKLR1	12q23.3	7.72	8.13	1.14	1.31	-0.41	6.97E-04	6.00E-03	(B) TP53 wild in GC
PPIP5K2	5q21.1	9.63	9.84	0.61	0.61	-0.21	7.01E-04	6.03E-03	(B) TP53 wild in GC
TP53	17p13.1	10.39	10.76	1.13	1.01	-0.37	7.04E-04	6.05E-03	(B) TP53 wild in GC
CREBL2	12p13.1	9.95	10.14	0.54	0.59	-0.19	7.05E-04	6.05E-03	(B) TP53 wild in GC
IL15	4q31.21	6.04	6.4	1.13	1.01	-0.37	7.13E-04	6.11E-03	(B) TP53 wild in GC
CCND2	12p13.32	10.63	11.15	1.53	1.54	-0.52	7.18E-04	6.13E-03	(B) TP53 wild in GC
CDKN1A	6p21.2	10.92	11.22	0.91	0.91	-0.31	7.23E-04	6.17E-03	(B) TP53 wild in GC

ARIH2	3p21.31	10.04	10.17	0.39	0.36	-0.13	7.25E-04	6.17E-03	(B) TP53 wild in GC
RAD17	5q13.2	8.77	8.92	0.44	0.4	-0.14	7.25E-04	6.17E-03	(B) TP53 wild in GC
HLA-DPB1	6p21.32	11.01	11.48	1.36	1.44	-0.47	7.30E-04	6.21E-03	(B) TP53 wild in GC
ZNF836	19q13.41	6.54	6.78	0.73	0.66	-0.24	7.33E-04	6.22E-03	(B) TP53 wild in GC
TRAPPC11	4q35.1	9.53	9.69	0.46	0.47	-0.16	7.39E-04	6.26E-03	(B) TP53 wild in GC
TLR4	9q33.1	8.04	8.43	1.16	1.14	-0.39	7.46E-04	6.31E-03	(B) TP53 wild in GC
ATXN7	3p14.1	9.43	9.59	0.49	0.47	-0.16	7.51E-04	6.34E-03	(B) TP53 wild in GC
STAB1	3p21.1	9.78	10.12	0.95	1.07	-0.34	7.53E-04	6.34E-03	(B) TP53 wild in GC
RSL24D1	15q21.3	10.31	10.49	0.55	0.5	-0.18	7.53E-04	6.35E-03	(B) TP53 wild in GC
ZZEF1	17p13.2	10.66	10.86	0.6	0.6	-0.2	7.59E-04	6.39E-03	(B) TP53 wild in GC
RAP1B	12q15	10.98	11.18	0.55	0.66	-0.2	7.63E-04	6.42E-03	(B) TP53 wild in GC
ZNF616	19q13.41	7	7.21	0.65	0.62	-0.21	7.68E-04	6.45E-03	(B) TP53 wild in GC
FNIP1	5q31.1	9.41	9.58	0.52	0.52	-0.17	7.71E-04	6.47E-03	(B) TP53 wild in GC
C2CD2	21q22.3	9.24	9.44	0.57	0.61	-0.2	7.77E-04	6.51E-03	(B) TP53 wild in GC
N6AMT1	21q21.3	7.17	7.35	0.54	0.53	-0.18	7.90E-04	6.60E-03	(B) TP53 wild in GC
PIGY	4q22.1	10.11	10.25	0.44	0.39	-0.14	7.95E-04	6.62E-03	(B) TP53 wild in GC

LPAR6	13q14.2	8.24	8.56	0.93	0.98	-0.32	8.04E-04	6.68E-03	(B) TP53 wild in GC
C5ORF56	5q31.1	6.46	6.75	0.83	0.87	-0.28	8.05E-04	6.68E-03	(B) TP53 wild in GC
CSNK1G3	5q23.2	9.33	9.49	0.49	0.44	-0.16	8.09E-04	6.71E-03	(B) TP53 wild in GC
GBP1	1p22.2	9.95	10.36	1.21	1.27	-0.41	8.15E-04	6.75E-03	(B) TP53 wild in GC
KIF27	9q21.32	6.19	6.46	0.83	0.75	-0.27	8.17E-04	6.76E-03	(B) TP53 wild in GC
MYOF	10q23.33	11.48	11.82	0.96	1.1	-0.34	8.36E-04	6.88E-03	(B) TP53 wild in GC
CD53	1p13.3	9.16	9.59	1.25	1.34	-0.43	8.37E-04	6.89E-03	(B) TP53 wild in GC
RB1	13q14.2	10.83	11.05	0.74	0.54	-0.22	8.48E-04	6.96E-03	(B) TP53 wild in GC
TMEM106	17q21.31	7.1	7.4	0.98	0.81	-0.3	8.48E-04	6.96E-03	(B) TP53 wild in GC
FKBP15	9q32	9.77	9.92	0.46	0.41	-0.15	8.52E-04	6.98E-03	(B) TP53 wild in GC
GNAQ	9q21.2	10.84	11.04	0.63	0.54	-0.2	8.53E-04	6.98E-03	(B) TP53 wild in GC
RAPGEF6	5q31.1	8.38	8.63	0.73	0.77	-0.25	8.54E-04	6.98E-03	(B) TP53 wild in GC
SAMSN1	21q11.2	7.46	7.88	1.22	1.27	-0.41	8.62E-04	7.04E-03	(B) TP53 wild in GC
ITPR1	3p26.1	8.54	8.93	1.12	1.27	-0.39	8.63E-04	7.05E-03	(B) TP53 wild in GC
WARS	14q32.2	12.02	12.39	0.96	1.29	-0.37	8.69E-04	7.07E-03	(B) TP53 wild in GC
DCAF16	4p15.31	9.49	9.66	0.52	0.49	-0.17	8.79E-04	7.14E-03	(B) TP53 wild in GC

PRMT9	4q31.23	7.12	7.29	0.54	0.47	-0.17	8.84E-04	7.17E-03	(B) TP53 wild in GC
RAB27A	15q21.3	9.28	9.54	0.75	0.79	-0.25	8.86E-04	7.18E-03	(B) TP53 wild in GC
CD28	2q33.2	5.65	6.11	1.37	1.46	-0.47	8.89E-04	7.20E-03	(B) TP53 wild in GC
RPL29	3p21.2	13.18	13.42	0.77	0.72	-0.25	9.01E-04	7.29E-03	(B) TP53 wild in GC
AZI2	3p24.1	9.44	9.57	0.38	0.41	-0.13	9.01E-04	7.29E-03	(B) TP53 wild in GC
MANBA	4q24	9.13	9.33	0.59	0.57	-0.19	9.17E-04	7.39E-03	(B) TP53 wild in GC
FAT1	4q35.2	12.59	12.89	0.89	0.88	-0.29	9.25E-04	7.44E-03	(B) TP53 wild in GC
TSPAN12	7q31.31	8.25	8.62	1.16	1.08	-0.37	9.31E-04	7.47E-03	(B) TP53 wild in GC
AKNA	9q32	10.14	10.42	0.81	0.9	-0.28	9.32E-04	7.48E-03	(B) TP53 wild in GC
SLC9A9	3q24	6.19	6.64	1.26	1.48	-0.45	9.35E-04	7.50E-03	(B) TP53 wild in GC
SEC24B	4q25	10.19	10.33	0.43	0.41	-0.14	9.47E-04	7.57E-03	(B) TP53 wild in GC
CSF1R	5q32	9.44	9.87	1.29	1.33	-0.43	9.50E-04	7.58E-03	(B) TP53 wild in GC
GRIN3A	9q31.1	4.24	4.6	1.14	1.01	-0.36	9.50E-04	7.58E-03	(B) TP53 wild in GC
SLC1A3	5p13.2	7.06	7.56	1.5	1.51	-0.5	9.59E-04	7.64E-03	(B) TP53 wild in GC
PIK3CD	1p36.22	8.27	8.63	1.04	1.16	-0.36	9.63E-04	7.65E-03	(B) TP53 wild in GC
MTR	1q43	9.79	9.99	0.57	0.61	-0.19	9.63E-04	7.65E-03	(B) TP53 wild in GC

BNIP3L	8p21.2	10.49	10.7	0.66	0.59	-0.21	9.69E-04	7.69E-03	(B) TP53 wild in GC
ARHGAP30	1q23.3	8.62	8.97	1.02	1.11	-0.35	9.72E-04	7.69E-03	(B) TP53 wild in GC
EIF4E	4q23	8.44	8.62	0.55	0.56	-0.18	9.75E-04	7.71E-03	(B) TP53 wild in GC
WDR7	18q21.31	8.77	8.99	0.68	0.62	-0.22	9.79E-04	7.73E-03	(B) TP53 wild in GC
RNF122	8p12	7.04	7.32	0.83	0.89	-0.28	9.87E-04	7.76E-03	(B) TP53 wild in GC
ZBTB7C	18q21.1	7.43	8.16	2.21	2.23	-0.73	9.87E-04	7.76E-03	(B) TP53 wild in GC
SLC12A2	5q23.3	11.6	12.02	1.26	1.29	-0.42	1.00E-03	7.86E-03	(B) TP53 wild in GC
TOM1L2	17p11.2	9.98	10.23	0.77	0.71	-0.25	1.00E-03	7.86E-03	(B) TP53 wild in GC
LOC	1001299q33.3	10.84	11.07	0.74	0.65	-0.23	1.01E-03	7.88E-03	(B) TP53 wild in GC
TENT5A	6q14.1	10.16	10.47	0.98	0.87	-0.31	1.01E-03	7.89E-03	(B) TP53 wild in GC
SAV1	14q22.1	9.33	9.52	0.57	0.6	-0.19	1.02E-03	7.93E-03	(B) TP53 wild in GC
POLH	6p21.1	9.09	9.3	0.65	0.6	-0.21	1.02E-03	7.95E-03	(B) TP53 wild in GC
ABR	17p13.3	11.47	11.64	0.52	0.53	-0.17	1.04E-03	8.05E-03	(B) TP53 wild in GC
C16ORF54	16p11.2	5.56	6.08	1.58	1.59	-0.52	1.04E-03	8.08E-03	(B) TP53 wild in GC
CD5	11q12.2	7.02	7.52	1.59	1.4	-0.5	1.05E-03	8.08E-03	(B) TP53 wild in GC
GCSAM	3q13.2	5.09	5.48	1.14	1.22	-0.39	1.05E-03	8.08E-03	(B) TP53 wild in GC

WBP1L	10q24.32	10.07	10.25	0.54	0.59	-0.18	1.05E-03	8.11E-03	(B) TP53 wild in GC
FLI1	11q24.3	7.95	8.28	0.94	1.11	-0.33	1.06E-03	8.14E-03	(B) TP53 wild in GC
PPM1L	3q25.33-q2	7.06	7.41	1.01	1.15	-0.35	1.07E-03	8.20E-03	(B) TP53 wild in GC
ZNF589	3p21.31	7.64	7.85	0.65	0.66	-0.21	1.07E-03	8.20E-03	(B) TP53 wild in GC
CLEC4A	12p13.31	5.14	5.52	1.14	1.22	-0.38	1.07E-03	8.20E-03	(B) TP53 wild in GC
JADE1	4q28.2	9.65	9.88	0.75	0.67	-0.24	1.07E-03	8.22E-03	(B) TP53 wild in GC
NISCH	3p21.1	10.35	10.53	0.55	0.6	-0.19	1.07E-03	8.24E-03	(B) TP53 wild in GC
RAB8B	15q22.2	9.21	9.42	0.62	0.69	-0.21	1.08E-03	8.26E-03	(B) TP53 wild in GC
PIGB	15q21.3	7.81	7.97	0.54	0.44	-0.16	1.08E-03	8.26E-03	(B) TP53 wild in GC
LRMP	12p12.1	5.8	6.37	1.77	1.73	-0.57	1.08E-03	8.29E-03	(B) TP53 wild in GC
ATP2B4	1q32.1	11.39	11.75	0.99	1.21	-0.36	1.09E-03	8.32E-03	(B) TP53 wild in GC
TRIM23	5q12.3	7.1	7.29	0.57	0.61	-0.19	1.11E-03	8.43E-03	(B) TP53 wild in GC
CD33	19q13.41	4.91	5.33	1.3	1.29	-0.42	1.11E-03	8.43E-03	(B) TP53 wild in GC
MOB3A	19p13.3	10.32	10.52	0.64	0.62	-0.21	1.11E-03	8.44E-03	(B) TP53 wild in GC
IL18BP	11q13.4	8.72	9.03	0.94	0.99	-0.31	1.13E-03	8.53E-03	(B) TP53 wild in GC
PSIP1	9p22.3	9.99	10.21	0.71	0.62	-0.22	1.13E-03	8.56E-03	(B) TP53 wild in GC

WAS	Xp11.23	7.58	7.98	1.27	1.21	-0.4	1.14E-03	8.57E-03	(B) TP53 wild in GC
RAP1GDS1	4q23	9.16	9.31	0.5	0.44	-0.15	1.14E-03	8.57E-03	(B) TP53 wild in GC
TBC1D2B	15q24.3-q2	10.17	10.37	0.6	0.63	-0.2	1.16E-03	8.67E-03	(B) TP53 wild in GC
CD74	5q33.1	15.21	15.64	1.32	1.3	-0.43	1.16E-03	8.67E-03	(B) TP53 wild in GC
KAT2B	3p24.3	8.72	8.99	0.81	0.9	-0.28	1.16E-03	8.67E-03	(B) TP53 wild in GC
LANCL1	2q34	10.29	10.53	0.72	0.78	-0.24	1.17E-03	8.73E-03	(B) TP53 wild in GC
DRAM1	12q23.2	9.28	9.55	0.86	0.86	-0.28	1.17E-03	8.73E-03	(B) TP53 wild in GC
GIMAP1	7q36.1	5.92	6.3	1.08	1.26	-0.38	1.17E-03	8.73E-03	(B) TP53 wild in GC
GNA13	17q24.1	11.29	11.45	0.5	0.43	-0.15	1.18E-03	8.81E-03	(B) TP53 wild in GC
CCNI	4q21.1	11.72	11.89	0.51	0.5	-0.16	1.18E-03	8.83E-03	(B) TP53 wild in GC
CCDC68	18q21.2	8.34	8.75	1.33	1.14	-0.41	1.19E-03	8.85E-03	(B) TP53 wild in GC
TRIB2	2p24.3	9.59	9.92	1.05	0.99	-0.33	1.20E-03	8.91E-03	(B) TP53 wild in GC
APOBEC3G	22q13.1	7.6	8	1.24	1.27	-0.41	1.25E-03	9.21E-03	(B) TP53 wild in GC
DGKA	12q13.2	8.96	9.27	0.97	0.96	-0.31	1.25E-03	9.21E-03	(B) TP53 wild in GC
ZNF18	17p12	7	7.17	0.6	0.48	-0.18	1.25E-03	9.23E-03	(B) TP53 wild in GC
DIPK2A	3q24	9.14	9.35	0.69	0.57	-0.21	1.25E-03	9.25E-03	(B) TP53 wild in GC

SLA	8q24.22	7.98	8.38	1.22	1.3	-0.4	1.26E-03	9.27E-03	(B) TP53 wild in GC
CAMTA2	17p13.2	10.14	10.3	0.47	0.48	-0.15	1.26E-03	9.28E-03	(B) TP53 wild in GC
ATG7	3p25.3	9.17	9.29	0.4	0.35	-0.12	1.27E-03	9.31E-03	(B) TP53 wild in GC
SARAF	8p12	11.95	12.12	0.55	0.49	-0.17	1.27E-03	9.32E-03	(B) TP53 wild in GC
CHD9	16q12.2	10.04	10.24	0.6	0.64	-0.2	1.29E-03	9.43E-03	(B) TP53 wild in GC
SLC4A4	4q13.3	8.38	9.12	2.33	2.23	-0.74	1.29E-03	9.46E-03	(B) TP53 wild in GC
TLR1	4p14	6.32	6.76	1.31	1.46	-0.44	1.29E-03	9.46E-03	(B) TP53 wild in GC
CASP7	10q25.3	9.84	10.06	0.69	0.72	-0.23	1.30E-03	9.50E-03	(B) TP53 wild in GC
TBC1D10C	11q13.2	6.12	6.6	1.45	1.52	-0.48	1.31E-03	9.54E-03	(B) TP53 wild in GC
NEK1	4q33	7.81	8	0.58	0.62	-0.19	1.31E-03	9.54E-03	(B) TP53 wild in GC
TNKS2	10q23.32	10.44	10.59	0.46	0.49	-0.15	1.32E-03	9.62E-03	(B) TP53 wild in GC
ZNF491	19p13.2	3.63	3.97	1.03	1.08	-0.34	1.33E-03	9.65E-03	(B) TP53 wild in GC
SASH3	Xq26.1	8.01	8.43	1.29	1.37	-0.43	1.33E-03	9.65E-03	(B) TP53 wild in GC
CD37	19q13.33	8.68	9.15	1.43	1.51	-0.47	1.34E-03	9.69E-03	(B) TP53 wild in GC
TAMM41	3p25.2	7.02	7.18	0.51	0.48	-0.16	1.35E-03	9.75E-03	(B) TP53 wild in GC
TMEM268	9q32	8.48	8.65	0.54	0.54	-0.17	1.35E-03	9.75E-03	(B) TP53 wild in GC

SCO1	17p13.1	8.66	8.84	0.55	0.53	-0.17	1.36E-03	9.82E-03	(B) TP53 wild in GC
CRYZL1	21q22.11	7.69	7.85	0.51	0.54	-0.17	1.36E-03	9.85E-03	(B) TP53 wild in GC
MYO1G	7p13	7.4	7.79	1.18	1.24	-0.39	1.37E-03	9.91E-03	(B) TP53 wild in GC
PLCB2	15q15.1	7.74	8.09	1.12	1.06	-0.35	1.38E-03	9.91E-03	(B) TP53 wild in GC
ASB8	12q13.11	9.05	9.17	0.36	0.39	-0.12	1.38E-03	9.91E-03	(B) TP53 wild in GC
CPED1	7q31.31	7.97	8.52	1.65	1.84	-0.55	1.38E-03	9.91E-03	(B) TP53 wild in GC
PIP5K1C	19p13.3	10.75	10.95	0.57	0.67	-0.2	1.38E-03	9.91E-03	(B) TP53 wild in GC
NLGN3	Xq13.1	5.09	5.51	1.25	1.37	-0.42	1.39E-03	9.96E-03	(B) TP53 wild in GC
BTN3A3	6p22.2	8.7	9.01	0.95	0.98	-0.31	1.39E-03	9.97E-03	(B) TP53 wild in GC
SLU7	5q33.3	9.76	9.92	0.54	0.42	-0.16	1.39E-03	9.97E-03	(B) TP53 wild in GC
NCBP3	17p13.2	9.1	9.25	0.46	0.45	-0.15	1.40E-03	0.01	(B) TP53 wild in GC
PIK3IP1	22q12.2	9.01	9.3	0.9	0.89	-0.29	1.40E-03	0.01	(B) TP53 wild in GC
EXOG	3p22.2	7.01	7.18	0.54	0.55	-0.17	1.40E-03	0.0101	(B) TP53 wild in GC
TRNT1	3p26.2	7.95	8.11	0.52	0.47	-0.16	1.40E-03	0.0101	(B) TP53 wild in GC
SP140	2q37.1	6.21	6.68	1.44	1.51	-0.47	1.41E-03	0.0101	(B) TP53 wild in GC
NSD1	5q35.3	11.03	11.19	0.51	0.49	-0.16	1.41E-03	0.0101	(B) TP53 wild in GC

ASPHD2	22q12.1	7.44	7.8	1.16	1.12	-0.36	1.44E-03	0.0103	(B) TP53 wild in GC
TLN1	9p13.3	13.25	13.5	0.72	0.86	-0.25	1.44E-03	0.0103	(B) TP53 wild in GC
ERCC8	5q12.1	7.02	7.17	0.49	0.46	-0.15	1.45E-03	0.0103	(B) TP53 wild in GC
MYO5C	15q21.2	10.06	10.35	0.95	0.87	-0.29	1.45E-03	0.0103	(B) TP53 wild in GC
IK	5q31.3	10.08	10.2	0.4	0.35	-0.12	1.46E-03	0.0103	(B) TP53 wild in GC
CGGBP1	3p11.1	10.84	10.97	0.43	0.4	-0.13	1.47E-03	0.0104	(B) TP53 wild in GC
PWWP3A	19p13.3	9.17	9.33	0.51	0.46	-0.16	1.47E-03	0.0105	(B) TP53 wild in GC
SOS2	14q21.3	9.74	9.9	0.51	0.54	-0.17	1.48E-03	0.0105	(B) TP53 wild in GC
ADA2	22q11.1	9.89	10.31	1.31	1.31	-0.42	1.49E-03	0.0105	(B) TP53 wild in GC
TUT7	9q21.33	9.61	9.79	0.59	0.52	-0.18	1.50E-03	0.0106	(B) TP53 wild in GC
MOCS2	5q11.2	9.04	9.21	0.52	0.56	-0.17	1.50E-03	0.0106	(B) TP53 wild in GC
ARFIP1	4q31.3	9.73	9.88	0.5	0.38	-0.14	1.51E-03	0.0106	(B) TP53 wild in GC
ZEB2	2q22.3	9.25	9.6	0.98	1.22	-0.35	1.51E-03	0.0106	(B) TP53 wild in GC
FOXP1	3p13	10.88	11.1	0.71	0.62	-0.21	1.51E-03	0.0106	(B) TP53 wild in GC
ADM5	19q13.33	4.8	5.14	1.07	1.06	-0.34	1.51E-03	0.0106	(B) TP53 wild in GC
IKZF3	17q12-q21	6.37	6.95	1.77	1.92	-0.58	1.52E-03	0.0107	(B) TP53 wild in GC

HADH	4q25	10.01	10.21	0.62	0.64	-0.2	1.53E-03	0.0107	(B) TP53 wild in GC
PDE10A	6q27	6.58	7.01	1.24	1.5	-0.43	1.55E-03	0.0108	(B) TP53 wild in GC
LAP3	4p15.32	10.98	11.21	0.68	0.78	-0.23	1.55E-03	0.0108	(B) TP53 wild in GC
AK3	9p24.1	10.77	10.97	0.65	0.58	-0.2	1.56E-03	0.0109	(B) TP53 wild in GC
SYNE1	6q25.2	9.26	9.71	1.31	1.55	-0.45	1.57E-03	0.0109	(B) TP53 wild in GC
FAM172A	5q15	8.62	8.82	0.65	0.67	-0.21	1.57E-03	0.0109	(B) TP53 wild in GC
SLFN12	17q12	6.02	6.37	1.11	1.09	-0.35	1.58E-03	0.011	(B) TP53 wild in GC
TNFSF13	17p13.1	9.44	9.78	1.09	1.06	-0.34	1.58E-03	0.011	(B) TP53 wild in GC
MATR3	5q31.2	12.14	12.26	0.37	0.34	-0.11	1.59E-03	0.011	(B) TP53 wild in GC
LMBR1L	12q13.12	8.17	8.32	0.49	0.45	-0.15	1.59E-03	0.011	(B) TP53 wild in GC
FMNL1	17q21.31	9.08	9.41	1.03	1.04	-0.33	1.60E-03	0.0111	(B) TP53 wild in GC
SLC35B4	7q33	8.64	8.81	0.52	0.56	-0.17	1.61E-03	0.0111	(B) TP53 wild in GC
SUSD6	14q24.1	10.48	10.66	0.54	0.58	-0.18	1.61E-03	0.0111	(B) TP53 wild in GC
RTL6	22q13.31	9.95	10.14	0.66	0.55	-0.19	1.62E-03	0.0111	(B) TP53 wild in GC
CD200R1	3q13.2	4.14	4.59	1.36	1.53	-0.45	1.62E-03	0.0111	(B) TP53 wild in GC
CFP	Xp11.23	4.84	5.25	1.23	1.38	-0.41	1.66E-03	0.0113	(B) TP53 wild in GC

ITPKB	1q42.12	9.56	9.89	0.96	1.14	-0.33	1.67E-03	0.0114	(B) TP53 wild in GC
USP16	21q21.3	9.66	9.81	0.5	0.45	-0.15	1.67E-03	0.0114	(B) TP53 wild in GC
APPL1	3p14.3	10.37	10.54	0.58	0.5	-0.17	1.68E-03	0.0114	(B) TP53 wild in GC
FMNL3	12q13.12	9.89	10.15	0.79	0.87	-0.26	1.69E-03	0.0115	(B) TP53 wild in GC
RGS1	1q31.2	9.72	10.16	1.46	1.35	-0.44	1.70E-03	0.0115	(B) TP53 wild in GC
ZNF621	3p22.1	9.05	9.22	0.54	0.54	-0.17	1.70E-03	0.0115	(B) TP53 wild in GC
P3H2	3q28	7.35	7.88	1.63	1.72	-0.52	1.70E-03	0.0115	(B) TP53 wild in GC
DDX5	17q23.3	12.92	13.04	0.39	0.37	-0.12	1.71E-03	0.0116	(B) TP53 wild in GC
ENDOV	17q25.3	6.27	6.52	0.85	0.77	-0.26	1.71E-03	0.0116	(B) TP53 wild in GC
ZFP90	16q22.1	8.54	8.71	0.52	0.55	-0.17	1.71E-03	0.0116	(B) TP53 wild in GC
MED31	17p13.1	6.55	6.74	0.59	0.56	-0.18	1.72E-03	0.0116	(B) TP53 wild in GC
ATP6V1B2	8p21.3	10.7	10.86	0.53	0.51	-0.16	1.73E-03	0.0117	(B) TP53 wild in GC
DENND1C	19p13.3	8.11	8.34	0.73	0.74	-0.23	1.73E-03	0.0117	(B) TP53 wild in GC
FAM160B2	8p21.3	10	10.17	0.53	0.51	-0.16	1.74E-03	0.0117	(B) TP53 wild in GC
PCDH18	4q28.3	8.9	9.22	1	1.06	-0.32	1.78E-03	0.012	(B) TP53 wild in GC
RBM43	2q23.3	7.22	7.47	0.81	0.85	-0.26	1.79E-03	0.012	(B) TP53 wild in GC

SYK	9q22.2	10.12	10.39	0.93	0.77	-0.27	1.79E-03	0.012	(B) TP53 wild in GC
LCP1	13q14.13	10.94	11.32	1.2	1.26	-0.38	1.79E-03	0.012	(B) TP53 wild in GC
CD274	9p24.1	5.01	5.49	1.43	1.65	-0.48	1.79E-03	0.012	(B) TP53 wild in GC
GPM6B	Xp22.2	5.67	6.1	1.35	1.44	-0.43	1.80E-03	0.012	(B) TP53 wild in GC
RELL1	4p14	9.16	9.37	0.63	0.69	-0.2	1.80E-03	0.012	(B) TP53 wild in GC
COX7C	5q14.3	10.88	11.09	0.7	0.66	-0.21	1.81E-03	0.0121	(B) TP53 wild in GC
EPB41L4A	5q22.1-q22	7.96	8.32	1.14	1.12	-0.35	1.82E-03	0.0121	(B) TP53 wild in GC
AFF4	5q31.1	11.3	11.49	0.57	0.68	-0.19	1.82E-03	0.0121	(B) TP53 wild in GC
ZNF136	19p13.2	7.32	7.55	0.74	0.74	-0.23	1.82E-03	0.0121	(B) TP53 wild in GC
CD84	1q23.3	6.64	7.14	1.59	1.63	-0.5	1.82E-03	0.0121	(B) TP53 wild in GC
HVCN1	12q24.11	6.9	7.25	1.09	1.17	-0.35	1.85E-03	0.0123	(B) TP53 wild in GC
SKP1	5q31.1	11.37	11.52	0.48	0.49	-0.15	1.85E-03	0.0123	(B) TP53 wild in GC
P2RY13	3q25.1	5.95	6.41	1.38	1.61	-0.46	1.85E-03	0.0123	(B) TP53 wild in GC
TCAF2	7q35	7.17	7.5	1.07	1.02	-0.33	1.85E-03	0.0123	(B) TP53 wild in GC
POU6F1	12q13.13	6.42	6.75	0.96	1.17	-0.33	1.86E-03	0.0123	(B) TP53 wild in GC
CYSLTR1	Xq21.1	4.73	5.2	1.51	1.51	-0.47	1.86E-03	0.0123	(B) TP53 wild in GC

BAG1	9p13.3	10.25	10.46	0.68	0.67	-0.21	1.87E-03	0.0124	(B) TP53 wild in GC
MYCBP2	13q22.3	10.74	10.94	0.63	0.63	-0.2	1.88E-03	0.0124	(B) TP53 wild in GC
DOK2	8p21.3	6.84	7.19	1.11	1.2	-0.36	1.89E-03	0.0124	(B) TP53 wild in GC
SGMS2	4q25	9.84	10.13	0.95	0.91	-0.29	1.89E-03	0.0125	(B) TP53 wild in GC
NCF4	22q12.3	7.11	7.46	1.09	1.13	-0.34	1.91E-03	0.0125	(B) TP53 wild in GC
CSF2RA	Xp22.33 an	6.38	6.81	1.31	1.5	-0.43	1.92E-03	0.0126	(B) TP53 wild in GC
AP1S2	Xp22.2	7.74	8.05	0.95	1.08	-0.31	1.92E-03	0.0126	(B) TP53 wild in GC
HEXB	5q13.3	11.17	11.34	0.55	0.52	-0.17	1.93E-03	0.0126	(B) TP53 wild in GC
AKAP13	15q25.3	11.88	12.07	0.6	0.66	-0.19	1.93E-03	0.0126	(B) TP53 wild in GC
USP4	3p21.31	9.86	9.98	0.41	0.35	-0.12	1.95E-03	0.0127	(B) TP53 wild in GC
PPM1D	17q23.2	8.39	8.52	0.4	0.42	-0.13	1.96E-03	0.0127	(B) TP53 wild in GC
NUDT12	5q21.2	8.03	8.42	1.31	1.2	-0.39	1.96E-03	0.0127	(B) TP53 wild in GC
HLA-DRA	6p21.32	13.28	13.72	1.44	1.35	-0.43	1.96E-03	0.0128	(B) TP53 wild in GC
PRRC1	5q23.2	10.68	10.82	0.45	0.49	-0.15	1.97E-03	0.0128	(B) TP53 wild in GC
ADCY6	12q13.12	9.9	10.1	0.67	0.59	-0.2	1.99E-03	0.0129	(B) TP53 wild in GC
ITPRID2	2q31.3	11.67	11.89	0.68	0.73	-0.22	2.00E-03	0.0129	(B) TP53 wild in GC

ZADH2	18q22.3	9.3	9.49	0.63	0.53	-0.18	2.00E-03	0.013	(B) TP53 wild in GC
BTG2	1q32.1	11.2	11.5	0.92	1.03	-0.3	2.01E-03	0.013	(B) TP53 wild in GC
BTN3A1	6p22.2	9.4	9.67	0.91	0.82	-0.27	2.01E-03	0.013	(B) TP53 wild in GC
S100B	21q22.3	5.11	5.57	1.39	1.64	-0.46	2.02E-03	0.0131	(B) TP53 wild in GC
HLA-DPA1	6p21.32	11.89	12.35	1.48	1.51	-0.46	2.03E-03	0.0131	(B) TP53 wild in GC
PIK3R5	17p13.1	7.1	7.49	1.26	1.32	-0.4	2.03E-03	0.0131	(B) TP53 wild in GC
ANKRD28	3p25.1	10.02	10.18	0.5	0.52	-0.16	2.03E-03	0.0131	(B) TP53 wild in GC
LY9	1q23.3	5.14	5.72	1.79	1.96	-0.57	2.04E-03	0.0131	(B) TP53 wild in GC
PLS3	Xq23	11.13	11.35	0.76	0.65	-0.22	2.04E-03	0.0131	(B) TP53 wild in GC
SEMA3B	3p21.31	9.23	9.66	1.51	1.24	-0.43	2.04E-03	0.0131	(B) TP53 wild in GC
ABHD10	3q13.2	9.18	9.33	0.46	0.52	-0.15	2.05E-03	0.0132	(B) TP53 wild in GC
HELQ	4q21.23	7.29	7.42	0.4	0.43	-0.13	2.05E-03	0.0132	(B) TP53 wild in GC
KDM4B	19p13.3	9.6	9.77	0.55	0.51	-0.16	2.07E-03	0.0133	(B) TP53 wild in GC
CABIN1	22q11.23	11.22	11.41	0.61	0.65	-0.19	2.08E-03	0.0133	(B) TP53 wild in GC
GNG7	19p13.3	6.3	6.74	1.38	1.53	-0.45	2.09E-03	0.0133	(B) TP53 wild in GC
LIAS	4p14	7.09	7.27	0.6	0.58	-0.18	2.09E-03	0.0133	(B) TP53 wild in GC

TGFBR1	9q22.33	10.33	10.56	0.69	0.79	-0.23	2.09E-03	0.0134	(B) TP53 wild in GC
BTRC	10q24.32	8.79	8.94	0.44	0.52	-0.15	2.10E-03	0.0134	(B) TP53 wild in GC
FER	5q21.3	5.87	6.16	0.94	0.94	-0.29	2.10E-03	0.0134	(B) TP53 wild in GC
WDR55	5q31.3	9.08	9.22	0.46	0.45	-0.14	2.11E-03	0.0134	(B) TP53 wild in GC
RHOH	4p14	6.48	6.91	1.31	1.47	-0.42	2.12E-03	0.0135	(B) TP53 wild in GC
MCTP1	5q15	6.64	7.02	1.1	1.36	-0.37	2.12E-03	0.0135	(B) TP53 wild in GC
ETF1	5q31.2	11.08	11.2	0.44	0.37	-0.13	2.13E-03	0.0135	(B) TP53 wild in GC
RUFY2	10q21.3	7.83	8	0.62	0.51	-0.18	2.13E-03	0.0135	(B) TP53 wild in GC
EFCAB14	1p33	11.98	12.13	0.49	0.45	-0.15	2.14E-03	0.0136	(B) TP53 wild in GC
SOCS2	12q22	7.91	8.2	0.95	0.97	-0.29	2.14E-03	0.0136	(B) TP53 wild in GC
APOL6	22q12.3	11.6	11.85	0.77	0.84	-0.25	2.15E-03	0.0136	(B) TP53 wild in GC
SPN	16p11.2	7.79	8.18	1.23	1.39	-0.4	2.15E-03	0.0136	(B) TP53 wild in GC
LINC02693	17p11.2	7.28	7.57	0.94	0.95	-0.29	2.16E-03	0.0136	(B) TP53 wild in GC
PIGL	17p11.2	6.54	6.75	0.74	0.67	-0.22	2.16E-03	0.0137	(B) TP53 wild in GC
CSF2RB	22q12.3	8.42	8.83	1.26	1.45	-0.41	2.17E-03	0.0137	(B) TP53 wild in GC
NCALD	8q22.3	7.5	7.85	1.07	1.2	-0.35	2.17E-03	0.0137	(B) TP53 wild in GC

TLR8	Xp22.2	5.51	6.03	1.62	1.81	-0.52	2.18E-03	0.0137	(B) TP53 wild in GC
CNOT6	5q35.3	9.92	10.06	0.46	0.44	-0.14	2.20E-03	0.0138	(B) TP53 wild in GC
ZNF224	19q13.31	7.13	7.38	0.83	0.73	-0.24	2.20E-03	0.0138	(B) TP53 wild in GC
KIAA0586	14q23.1	8.37	8.54	0.57	0.54	-0.17	2.20E-03	0.0138	(B) TP53 wild in GC
INPP5F	10q26.11	8.64	8.82	0.55	0.65	-0.18	2.22E-03	0.0139	(B) TP53 wild in GC
KLHL28	14q21.2	8.5	8.66	0.54	0.48	-0.16	2.22E-03	0.0139	(B) TP53 wild in GC
CD59	11p13	12.35	12.59	0.75	0.81	-0.24	2.22E-03	0.0139	(B) TP53 wild in GC
CLUAP1	16p13.3	8.13	8.31	0.61	0.57	-0.18	2.23E-03	0.0139	(B) TP53 wild in GC
C12ORF4	12p13.32	8.29	8.45	0.53	0.48	-0.16	2.23E-03	0.0139	(B) TP53 wild in GC
ICAM2	17q23.3	7.96	8.25	0.91	1.04	-0.3	2.24E-03	0.014	(B) TP53 wild in GC
HLA-F-AS1	6p22.1	4.76	5.07	0.97	1.09	-0.31	2.25E-03	0.014	(B) TP53 wild in GC
RAPGEF2	4q32.1	9.59	9.76	0.54	0.55	-0.17	2.25E-03	0.014	(B) TP53 wild in GC
HNRNPA1	12q13.13	12.17	12.33	0.49	0.5	-0.15	2.27E-03	0.0141	(B) TP53 wild in GC
ISCU	12q23.3	10.32	10.47	0.48	0.52	-0.15	2.27E-03	0.0141	(B) TP53 wild in GC
USP15	12q14.1	9.93	10.08	0.49	0.49	-0.15	2.27E-03	0.0141	(B) TP53 wild in GC
MPEG1	11q12.1	9.36	9.78	1.32	1.44	-0.42	2.28E-03	0.0141	(B) TP53 wild in GC

ATP5F1A	18q21.1	12.55	12.73	0.62	0.57	-0.18	2.28E-03	0.0142	(B) TP53 wild in GC
IL21R	16p12.1	5.97	6.42	1.5	1.5	-0.46	2.30E-03	0.0143	(B) TP53 wild in GC
PCM1	8p22	11.07	11.28	0.74	0.6	-0.21	2.31E-03	0.0143	(B) TP53 wild in GC
DPYD	1p21.3	8.42	8.82	1.23	1.39	-0.4	2.31E-03	0.0143	(B) TP53 wild in GC
AIF1	6p21.33	8.09	8.43	1.11	1.2	-0.35	2.33E-03	0.0144	(B) TP53 wild in GC
NUMB	14q24.2-q2	10.85	10.95	0.35	0.37	-0.11	2.35E-03	0.0145	(B) TP53 wild in GC
CCDC66	3p14.3	7.52	7.72	0.71	0.63	-0.21	2.35E-03	0.0145	(B) TP53 wild in GC
RPL34	4q25	11.99	12.22	0.77	0.75	-0.23	2.36E-03	0.0145	(B) TP53 wild in GC
PARP3	3p21.2	8.63	8.86	0.8	0.76	-0.24	2.36E-03	0.0145	(B) TP53 wild in GC
POGLUT1	3q13.33	8.54	8.68	0.46	0.46	-0.14	2.36E-03	0.0146	(B) TP53 wild in GC
FOLR2	11q13.4	6.53	7.05	1.69	1.74	-0.52	2.37E-03	0.0146	(B) TP53 wild in GC
KLF12	13q22.1	8.01	8.4	1.27	1.28	-0.39	2.37E-03	0.0146	(B) TP53 wild in GC
QKI	6q26	10.07	10.34	0.85	0.95	-0.27	2.38E-03	0.0146	(B) TP53 wild in GC
SNX2	5q23.2	10.08	10.23	0.5	0.48	-0.15	2.38E-03	0.0146	(B) TP53 wild in GC
RPS14	5q33.1	13.45	13.64	0.66	0.61	-0.19	2.38E-03	0.0146	(B) TP53 wild in GC
SIDT1	3q13.2	6.71	7.23	1.81	1.55	-0.52	2.40E-03	0.0147	(B) TP53 wild in GC

PRDM1	6q21	9.78	10.06	0.94	0.92	-0.28	2.40E-03	0.0147	(B) TP53 wild in GC
FRYL	4p11	10.46	10.66	0.71	0.59	-0.2	2.42E-03	0.0148	(B) TP53 wild in GC
SLFN12L	17q12	4.37	4.81	1.43	1.48	-0.44	2.43E-03	0.0148	(B) TP53 wild in GC
BANK1	4q24	6.43	6.98	1.8	1.86	-0.55	2.43E-03	0.0149	(B) TP53 wild in GC
COPS3	17p11.2	9.69	9.83	0.48	0.44	-0.14	2.43E-03	0.0149	(B) TP53 wild in GC
ADARB1	21q22.3	8.45	8.72	0.89	0.93	-0.28	2.44E-03	0.0149	(B) TP53 wild in GC
TRANK1	3p22.2	9.28	9.57	0.91	0.99	-0.29	2.45E-03	0.015	(B) TP53 wild in GC
TLN2	15q22.2	9.56	9.88	1.11	0.96	-0.32	2.46E-03	0.015	(B) TP53 wild in GC
GBP2	1p22.2	10.47	10.79	1	1.1	-0.31	2.47E-03	0.015	(B) TP53 wild in GC
VCAM1	1p21.2	8.82	9.18	1.12	1.24	-0.36	2.47E-03	0.015	(B) TP53 wild in GC
PTPRCAP	11q13.2	7.76	8.23	1.55	1.57	-0.47	2.48E-03	0.0151	(B) TP53 wild in GC
CIITA	16p13.13	8.69	9.16	1.54	1.6	-0.47	2.52E-03	0.0152	(B) TP53 wild in GC
ARID5B	10q21.2	10.6	10.85	0.84	0.79	-0.25	2.53E-03	0.0153	(B) TP53 wild in GC
EEF1A1P9	4q24	7.37	7.85	1.59	1.64	-0.49	2.55E-03	0.0154	(B) TP53 wild in GC
CST7	20p11.21	7.03	7.45	1.42	1.44	-0.43	2.60E-03	0.0156	(B) TP53 wild in GC
STIM2	4p15.2	9.2	9.38	0.61	0.53	-0.17	2.61E-03	0.0157	(B) TP53 wild in GC

ARHGDIB	12p12.3	10.68	10.97	0.95	0.97	-0.29	2.62E-03	0.0157	(B) TP53 wild in GC
NUDT2	9p13.3	7.47	7.68	0.7	0.7	-0.21	2.65E-03	0.0159	(B) TP53 wild in GC
TLCD3A	17p13.3	9.39	9.6	0.64	0.78	-0.21	2.69E-03	0.016	(B) TP53 wild in GC
SNX20	16q12.1	5.76	6.15	1.24	1.37	-0.39	2.70E-03	0.0161	(B) TP53 wild in GC
ABL1	9q34.12	11.31	11.48	0.56	0.62	-0.18	2.70E-03	0.0161	(B) TP53 wild in GC
GUF1	4p12	9.76	9.92	0.55	0.51	-0.16	2.72E-03	0.0162	(B) TP53 wild in GC
TRIM38	6p22.2	9.02	9.22	0.71	0.63	-0.2	2.72E-03	0.0162	(B) TP53 wild in GC
PLA2G4A	1q31.1	7.94	8.4	1.63	1.43	-0.46	2.74E-03	0.0162	(B) TP53 wild in GC
PARVG	22q13.31	7.25	7.59	1.12	1.16	-0.34	2.75E-03	0.0163	(B) TP53 wild in GC
LRRN3	7q31.1	3.45	3.89	1.42	1.51	-0.44	2.77E-03	0.0164	(B) TP53 wild in GC
ALDH3A2	17p11.2	10.54	10.76	0.72	0.7	-0.21	2.78E-03	0.0164	(B) TP53 wild in GC
GIN1	5q21.1	6.43	6.6	0.51	0.61	-0.17	2.78E-03	0.0164	(B) TP53 wild in GC
TMEM131L	4q31.3	8.3	8.57	0.87	0.91	-0.26	2.80E-03	0.0165	(B) TP53 wild in GC
SLC35A5	3q13.2	9.16	9.31	0.47	0.52	-0.15	2.80E-03	0.0165	(B) TP53 wild in GC
TOP2B	3p24.2	11.14	11.3	0.56	0.55	-0.17	2.83E-03	0.0166	(B) TP53 wild in GC
CCL4	17q12	7.17	7.61	1.47	1.46	-0.44	2.85E-03	0.0167	(B) TP53 wild in GC

C3AR1	12p13.31	7.77	8.16	1.28	1.38	-0.39	2.87E-03	0.0168	(B) TP53 wild in GC
NUP88	17p13.2	9.7	9.85	0.48	0.49	-0.14	2.87E-03	0.0168	(B) TP53 wild in GC
CXORF21	Xp21.2	4.86	5.23	1.18	1.27	-0.36	2.88E-03	0.0168	(B) TP53 wild in GC
ZNF652	17q21.32-q	10.38	10.52	0.46	0.48	-0.14	2.89E-03	0.0169	(B) TP53 wild in GC
ZNF45	19q13.31	7.63	7.81	0.69	0.5	-0.18	2.90E-03	0.0169	(B) TP53 wild in GC
GNB4	3q26.33	9.44	9.77	1.04	1.17	-0.33	2.90E-03	0.0169	(B) TP53 wild in GC
BDH2	4q24	8.02	8.29	0.92	0.83	-0.26	2.91E-03	0.017	(B) TP53 wild in GC
LIMA1	12q13.12	11.7	11.94	0.79	0.81	-0.24	2.93E-03	0.0171	(B) TP53 wild in GC
CFL2	14q13.1	8.81	9.2	1.18	1.43	-0.38	2.93E-03	0.0171	(B) TP53 wild in GC
ADH5	4q23	10.65	10.81	0.53	0.52	-0.16	2.94E-03	0.0171	(B) TP53 wild in GC
KDM4C	9p24.1	9.18	9.36	0.64	0.53	-0.18	2.94E-03	0.0171	(B) TP53 wild in GC
ARHGAP25	2p13.3	7.63	7.96	1.1	1.17	-0.34	2.96E-03	0.0172	(B) TP53 wild in GC
RACK1	5q35.3	14.23	14.41	0.59	0.62	-0.18	2.96E-03	0.0172	(B) TP53 wild in GC
ITGB2	21q22.3	9.87	10.27	1.38	1.33	-0.4	2.98E-03	0.0173	(B) TP53 wild in GC
ZFAND5	9q21.13	11.59	11.73	0.51	0.42	-0.14	2.98E-03	0.0173	(B) TP53 wild in GC
EIF4E3	3p13	8.9	9.17	0.88	0.97	-0.27	2.98E-03	0.0173	(B) TP53 wild in GC

PTEN	10q23.31	10.68	10.86	0.57	0.63	-0.18	2.99E-03	0.0173	(B) TP53 wild in GC
ERAP2	5q15	9.38	9.93	1.88	1.81	-0.55	2.99E-03	0.0173	(B) TP53 wild in GC
WASHC2C	10q11.22	10.12	10.25	0.47	0.44	-0.14	3.00E-03	0.0174	(B) TP53 wild in GC
ZNF791	19p13.13	8.22	8.38	0.55	0.55	-0.16	3.00E-03	0.0174	(B) TP53 wild in GC
DIS3L	15q22.31	9.05	9.22	0.58	0.49	-0.16	3.02E-03	0.0175	(B) TP53 wild in GC
TPK1	7q35	6.87	7.17	1.08	0.94	-0.3	3.03E-03	0.0175	(B) TP53 wild in GC
TRIM35	8p21.2	8.76	8.91	0.52	0.48	-0.15	3.03E-03	0.0175	(B) TP53 wild in GC
ZFAND6	15q25.1	9.82	9.95	0.4	0.45	-0.12	3.04E-03	0.0175	(B) TP53 wild in GC
EPS15	1p32.3	10.49	10.62	0.45	0.45	-0.13	3.04E-03	0.0175	(B) TP53 wild in GC
SEPTIN8	5q31.1	10.36	10.49	0.44	0.42	-0.13	3.05E-03	0.0175	(B) TP53 wild in GC
SAMD9L	7q21.2	9.68	10.08	1.36	1.32	-0.4	3.07E-03	0.0176	(B) TP53 wild in GC
TARSL2	15q26.3	7.58	7.78	0.64	0.68	-0.2	3.07E-03	0.0176	(B) TP53 wild in GC
ZNF627	19p13.2	7.96	8.11	0.51	0.44	-0.14	3.08E-03	0.0177	(B) TP53 wild in GC
SCIMP	17p13.2	5.04	5.49	1.48	1.6	-0.45	3.11E-03	0.0178	(B) TP53 wild in GC
SLC4A5	2p13.1	7.33	7.55	0.72	0.73	-0.21	3.13E-03	0.0179	(B) TP53 wild in GC
RBM27	5q32	9.9	10	0.37	0.34	-0.1	3.15E-03	0.0179	(B) TP53 wild in GC

PPP1R12B	1q32.1	10.85	11.33	1.48	1.81	-0.48	3.15E-03	0.0179	(B) TP53 wild in GC
APPBP2	17q23.2	9.66	9.79	0.42	0.47	-0.13	3.17E-03	0.018	(B) TP53 wild in GC
NPR2	9p13.3	7.09	7.36	0.91	0.93	-0.27	3.18E-03	0.018	(B) TP53 wild in GC
IDO1	8p11.21	7.51	8.2	2.17	2.52	-0.69	3.18E-03	0.018	(B) TP53 wild in GC
DNAJB14	4q23	8.23	8.38	0.52	0.51	-0.15	3.18E-03	0.0181	(B) TP53 wild in GC
POLR2B	4q12	10.88	11	0.43	0.43	-0.13	3.21E-03	0.0182	(B) TP53 wild in GC
USP38	4q31.21	9.91	10.05	0.54	0.45	-0.15	3.22E-03	0.0182	(B) TP53 wild in GC
KIAA2026	9p24.1	9.83	10.03	0.73	0.6	-0.2	3.23E-03	0.0182	(B) TP53 wild in GC
RGMB	5q15	9.38	9.67	0.94	1.08	-0.29	3.23E-03	0.0182	(B) TP53 wild in GC
GBE1	3p12.2	8.43	8.6	0.58	0.62	-0.18	3.24E-03	0.0183	(B) TP53 wild in GC
AP5M1	14q22.3	9.65	9.79	0.48	0.46	-0.14	3.26E-03	0.0184	(B) TP53 wild in GC
LINC00663	19p13.11	4.31	4.59	0.89	1.01	-0.28	3.27E-03	0.0184	(B) TP53 wild in GC
LRFN3	19q13.12	8.68	8.88	0.78	0.54	-0.2	3.27E-03	0.0184	(B) TP53 wild in GC
PHTF2	7q11.23-q2	8.8	8.97	0.57	0.56	-0.17	3.27E-03	0.0184	(B) TP53 wild in GC
NOL8	9q22.31	9.39	9.54	0.54	0.49	-0.15	3.27E-03	0.0184	(B) TP53 wild in GC
DENND4C	9p22.1	10.41	10.6	0.65	0.58	-0.18	3.30E-03	0.0185	(B) TP53 wild in GC

LAIR1	19q13.42	8.45	8.81	1.19	1.23	-0.35	3.32E-03	0.0186	(B) TP53 wild in GC
CLEC2B	12p13.31	7.26	7.58	1.07	1.14	-0.32	3.32E-03	0.0186	(B) TP53 wild in GC
POLK	5q13.3	8.59	8.77	0.56	0.66	-0.18	3.33E-03	0.0186	(B) TP53 wild in GC
EVI5	1p22.1	9.17	9.32	0.52	0.55	-0.16	3.38E-03	0.0188	(B) TP53 wild in GC
COPS4	4q21.22	9.23	9.34	0.39	0.41	-0.12	3.38E-03	0.0188	(B) TP53 wild in GC
HLA-DOA	6p21.32	8.23	8.73	1.7	1.75	-0.5	3.42E-03	0.019	(B) TP53 wild in GC
CTTNBP2N	1p13.2	9.87	10.02	0.49	0.53	-0.15	3.43E-03	0.0191	(B) TP53 wild in GC
PSTPIP1	15q24.3	5.86	6.19	1.11	1.19	-0.33	3.46E-03	0.0192	(B) TP53 wild in GC
SETD2	3p21.31	10.89	11.03	0.48	0.49	-0.14	3.47E-03	0.0192	(B) TP53 wild in GC
GSAP	7q11.23	8.11	8.35	0.86	0.78	-0.24	3.47E-03	0.0192	(B) TP53 wild in GC
FAM49A	2p24.2	6.06	6.44	1.21	1.4	-0.38	3.49E-03	0.0193	(B) TP53 wild in GC
ADAM19	5q33.3	9.33	9.61	0.93	1.05	-0.29	3.52E-03	0.0194	(B) TP53 wild in GC
ZNF430	19p12	7.08	7.29	0.78	0.69	-0.22	3.53E-03	0.0195	(B) TP53 wild in GC
ZNF230	19q13.31	6.15	6.34	0.72	0.6	-0.2	3.56E-03	0.0196	(B) TP53 wild in GC
ANKRD34A	1q21.1	4.65	4.94	0.99	1.02	-0.29	3.56E-03	0.0196	(B) TP53 wild in GC
CLIC4	1p36.11	11.56	11.85	0.92	1.05	-0.28	3.58E-03	0.0196	(B) TP53 wild in GC

HCST	19q13.12	6.65	7.05	1.34	1.38	-0.39	3.68E-03	0.0201	(B) TP53 wild in GC
GTPBP8	3q13.2	7.12	7.25	0.45	0.45	-0.13	3.68E-03	0.0201	(B) TP53 wild in GC
PEAK1	15q24.3	10.55	10.75	0.63	0.7	-0.19	3.72E-03	0.0203	(B) TP53 wild in GC
ABI3	17q21.32	7.98	8.25	0.93	1	-0.28	3.74E-03	0.0204	(B) TP53 wild in GC
PPP1R12A	12q21.2-q2	10.71	10.9	0.62	0.74	-0.19	3.74E-03	0.0204	(B) TP53 wild in GC
EXOC5	14q22.3	10.08	10.22	0.46	0.44	-0.13	3.75E-03	0.0204	(B) TP53 wild in GC
NAV1	1q32.1	10.03	10.28	0.83	0.92	-0.25	3.76E-03	0.0204	(B) TP53 wild in GC
RECK	9p13.3	7.17	7.51	1.1	1.31	-0.35	3.77E-03	0.0205	(B) TP53 wild in GC
HAGHL	16p13.3	6.18	6.58	1.48	1.25	-0.4	3.79E-03	0.0206	(B) TP53 wild in GC
CLASP2	3p22.3	9.64	9.79	0.52	0.5	-0.15	3.83E-03	0.0207	(B) TP53 wild in GC
TUBGCP5	15q11.2	8	8.16	0.58	0.54	-0.16	3.83E-03	0.0207	(B) TP53 wild in GC
BMPR2	2q33.1-q33	11.32	11.5	0.58	0.64	-0.18	3.85E-03	0.0208	(B) TP53 wild in GC
CCDC69	5q33.1	9.02	9.41	1.26	1.48	-0.39	3.89E-03	0.021	(B) TP53 wild in GC
SAT1	Xp22.11	12.23	12.47	0.8	0.87	-0.24	3.90E-03	0.021	(B) TP53 wild in GC
ETHE1	19q13.31	9.99	10.27	0.95	0.97	-0.28	3.90E-03	0.021	(B) TP53 wild in GC
EIF4EBP2	10q22.1	12.09	12.24	0.56	0.46	-0.15	3.90E-03	0.021	(B) TP53 wild in GC

CX3CR1	3p22.2	4.87	5.33	1.44	1.76	-0.46	3.91E-03	0.021	(B) TP53 wild in GC
HARS	5q31.3	9.8	9.91	0.4	0.35	-0.11	3.92E-03	0.021	(B) TP53 wild in GC
TBX21	17q21.32	3.84	4.28	1.52	1.56	-0.44	3.92E-03	0.021	(B) TP53 wild in GC
INTS12	4q24	8.42	8.55	0.49	0.45	-0.14	3.93E-03	0.0211	(B) TP53 wild in GC
LAPTM5	1p35.2	11.58	11.92	1.15	1.21	-0.34	3.93E-03	0.0211	(B) TP53 wild in GC
SRFBP1	5q23.1	7.86	8	0.51	0.46	-0.14	3.95E-03	0.0211	(B) TP53 wild in GC
ERCC6L2	9q22.32	7.24	7.47	0.81	0.8	-0.23	3.96E-03	0.0212	(B) TP53 wild in GC
MMP28	17q12	7.23	7.73	1.61	1.86	-0.5	3.97E-03	0.0212	(B) TP53 wild in GC
KLHL6	3q27.1	7.41	7.81	1.3	1.47	-0.39	3.98E-03	0.0213	(B) TP53 wild in GC
ZNF223	19q13.31	5.62	5.92	1.16	0.9	-0.31	3.98E-03	0.0213	(B) TP53 wild in GC
RCHY1	4q21.1	8.53	8.68	0.53	0.54	-0.15	3.99E-03	0.0213	(B) TP53 wild in GC
ZNF330	4q31.21	8.74	8.88	0.51	0.47	-0.14	3.99E-03	0.0213	(B) TP53 wild in GC
POLR1E	9p13.2	8.47	8.62	0.54	0.52	-0.15	4.01E-03	0.0214	(B) TP53 wild in GC
LAX1	1q32.1	5.67	6.15	1.69	1.69	-0.49	4.02E-03	0.0214	(B) TP53 wild in GC
DPCD	10q24.32	7.31	7.52	0.74	0.69	-0.21	4.03E-03	0.0214	(B) TP53 wild in GC
LYST	1q42.3	9.21	9.42	0.68	0.79	-0.21	4.03E-03	0.0215	(B) TP53 wild in GC

ZBTB46	20q13.33	7.11	7.39	0.97	0.98	-0.28	4.04E-03	0.0215	(B) TP53 wild in GC
KLHL9	9p21.3	9.49	9.72	0.83	0.79	-0.23	4.06E-03	0.0216	(B) TP53 wild in GC
RPAIN	17p13.2	8.77	8.93	0.58	0.53	-0.16	4.06E-03	0.0216	(B) TP53 wild in GC
SEC22C	3p22.1	8.26	8.39	0.48	0.43	-0.13	4.07E-03	0.0216	(B) TP53 wild in GC
UBA5	3q22.1	9.23	9.35	0.45	0.39	-0.12	4.08E-03	0.0216	(B) TP53 wild in GC
TFAP2A	6p24.3	8.01	8.52	1.95	1.52	-0.51	4.08E-03	0.0216	(B) TP53 wild in GC
PTCD2	5q13.2	6.68	6.83	0.55	0.47	-0.15	4.08E-03	0.0216	(B) TP53 wild in GC
RYR3	15q13.3-q1	3.3	3.81	1.66	1.99	-0.52	4.10E-03	0.0217	(B) TP53 wild in GC
MIS12	17p13.2	8.31	8.44	0.5	0.41	-0.13	4.10E-03	0.0217	(B) TP53 wild in GC
EMB	5q11.1	9.64	10.03	1.39	1.28	-0.39	4.11E-03	0.0217	(B) TP53 wild in GC
GBP5	1p22.2	8.34	8.85	1.76	1.75	-0.5	4.11E-03	0.0217	(B) TP53 wild in GC
ERCC5	13q33.1	10.13	10.27	0.54	0.47	-0.15	4.13E-03	0.0218	(B) TP53 wild in GC
ANK3	10q21.2	8.97	9.25	1.08	0.79	-0.28	4.15E-03	0.0219	(B) TP53 wild in GC
RBPJ	4p15.2	9.88	10.02	0.49	0.47	-0.14	4.16E-03	0.0219	(B) TP53 wild in GC
ATP9B	18q23	8.69	8.86	0.66	0.54	-0.17	4.17E-03	0.022	(B) TP53 wild in GC
SLC30A5	5q13.1-q13	9.9	10.01	0.4	0.39	-0.11	4.18E-03	0.022	(B) TP53 wild in GC

ZNF543	19q13.43	7.14	7.35	0.8	0.72	-0.22	4.20E-03	0.0221	(B) TP53 wild in GC
SPTLC2	14q24.3	10.7	10.87	0.61	0.63	-0.18	4.22E-03	0.0221	(B) TP53 wild in GC
ZC3HAV1	7q34	10.78	10.9	0.41	0.4	-0.12	4.27E-03	0.0224	(B) TP53 wild in GC
SLC8A1	2p22.1	7.94	8.28	1.08	1.33	-0.34	4.30E-03	0.0225	(B) TP53 wild in GC
SLC25A23	19p13.3	10.94	11.17	0.72	0.86	-0.22	4.30E-03	0.0225	(B) TP53 wild in GC
ZNF317	19p13.2	9.69	9.83	0.57	0.31	-0.14	4.34E-03	0.0227	(B) TP53 wild in GC
CAAP1	9p21.2	8.64	8.87	0.81	0.81	-0.23	4.37E-03	0.0228	(B) TP53 wild in GC
ADAM28	8p21.2	8.19	8.7	1.79	1.81	-0.51	4.39E-03	0.0229	(B) TP53 wild in GC
TOX	8q12.1	7.2	7.7	1.86	1.65	-0.5	4.40E-03	0.0229	(B) TP53 wild in GC
GPR155	2q31.1	7.9	8.28	1.33	1.37	-0.38	4.41E-03	0.023	(B) TP53 wild in GC
SAR1A	10q22.1	10.5	10.61	0.44	0.38	-0.12	4.41E-03	0.023	(B) TP53 wild in GC
ANKHD1-EI	5q31.3	9.84	10.03	0.71	0.67	-0.2	4.42E-03	0.023	(B) TP53 wild in GC
GPR107	9q34.11	11.13	11.27	0.45	0.55	-0.14	4.44E-03	0.0231	(B) TP53 wild in GC
SLAMF1	1q23.3	5.02	5.43	1.43	1.45	-0.41	4.47E-03	0.0232	(B) TP53 wild in GC
MICU3	8p22	4.7	5.11	1.31	1.59	-0.41	4.47E-03	0.0232	(B) TP53 wild in GC
CD244	1q23.3	3.92	4.35	1.46	1.57	-0.43	4.50E-03	0.0233	(B) TP53 wild in GC

IRF4	6p25.3	6.58	7.06	1.68	1.75	-0.48	4.51E-03	0.0233	(B) TP53 wild in GC
ISOC1	5q23.3	9.2	9.36	0.56	0.53	-0.16	4.54E-03	0.0234	(B) TP53 wild in GC
PPA2	4q24	9.77	9.92	0.52	0.54	-0.15	4.55E-03	0.0235	(B) TP53 wild in GC
SHPK	17p13.2	8.92	9.08	0.57	0.57	-0.16	4.56E-03	0.0235	(B) TP53 wild in GC
CALHM6	6q22.1	6.1	6.54	1.57	1.54	-0.44	4.57E-03	0.0235	(B) TP53 wild in GC
PARP15	3q21.1	4.71	5.2	1.73	1.79	-0.5	4.57E-03	0.0235	(B) TP53 wild in GC
OXNAD1	3p25.1-p24	7.93	8.07	0.51	0.48	-0.14	4.59E-03	0.0236	(B) TP53 wild in GC
FGL2	7q11.23	9.37	9.82	1.5	1.69	-0.45	4.60E-03	0.0236	(B) TP53 wild in GC
SLC25A38	3p22.1	9.18	9.3	0.47	0.41	-0.13	4.64E-03	0.0238	(B) TP53 wild in GC
PDE8B	5q13.3	4.73	5.17	1.49	1.63	-0.44	4.66E-03	0.0239	(B) TP53 wild in GC
IFFO1	12p13.31	6.78	7.05	0.9	1.02	-0.27	4.67E-03	0.0239	(B) TP53 wild in GC
GPR183	13q32.3	7.81	8.16	1.21	1.31	-0.35	4.67E-03	0.0239	(B) TP53 wild in GC
SNHG29	17p11.2	11.6	11.82	0.79	0.81	-0.23	4.72E-03	0.0241	(B) TP53 wild in GC
HTR2B	2q37.1	4.51	5.01	1.62	1.91	-0.49	4.74E-03	0.0241	(B) TP53 wild in GC
CNIH1	14q22.2	10.64	10.78	0.49	0.49	-0.14	4.75E-03	0.0242	(B) TP53 wild in GC
RAPGEF1	9q34.13	11.09	11.25	0.57	0.58	-0.16	4.77E-03	0.0242	(B) TP53 wild in GC

C9ORF85	9q21.13	7.11	7.26	0.6	0.5	-0.16	4.78E-03	0.0243	(B) TP53 wild in GC
POLI	18q21.2	7.75	7.95	0.71	0.7	-0.2	4.79E-03	0.0243	(B) TP53 wild in GC
ESYT2	7q36.3	11.41	11.57	0.58	0.6	-0.16	4.83E-03	0.0245	(B) TP53 wild in GC
FILIP1L	3q12.1	10.63	10.94	1	1.22	-0.31	4.83E-03	0.0245	(B) TP53 wild in GC
ZNF215	11p15.4	4.76	5.26	1.89	1.63	-0.5	4.84E-03	0.0245	(B) TP53 wild in GC
APOL3	22q12.3	8.8	9.13	1.11	1.23	-0.33	4.85E-03	0.0245	(B) TP53 wild in GC
AASDH	4q12	7.5	7.64	0.53	0.49	-0.14	4.85E-03	0.0245	(B) TP53 wild in GC
GFI1	1p22.1	5.59	5.96	1.38	1.22	-0.37	4.87E-03	0.0246	(B) TP53 wild in GC
RBM3	Xp11.23	11.31	11.48	0.61	0.53	-0.16	4.87E-03	0.0246	(B) TP53 wild in GC
SNHG8	4q26	8.64	8.85	0.74	0.76	-0.21	4.88E-03	0.0246	(B) TP53 wild in GC
C1R	12p13.31	11.65	12.01	1.2	1.33	-0.35	4.89E-03	0.0246	(B) TP53 wild in GC
FRS2	12q15	9.75	9.93	0.59	0.75	-0.19	4.89E-03	0.0246	(B) TP53 wild in GC
ZNF575	19q13.31	5.82	6.01	0.7	0.71	-0.2	4.90E-03	0.0247	(B) TP53 wild in GC
CD27	12p13.31	6.76	7.25	1.77	1.68	-0.49	4.90E-03	0.0247	(B) TP53 wild in GC
TNFAIP8L2	1q21.3	5.79	6.1	1.09	1.13	-0.31	4.93E-03	0.0248	(B) TP53 wild in GC
TVP23C	17p12	8.29	8.48	0.7	0.68	-0.19	4.94E-03	0.0248	(B) TP53 wild in GC

NEK9	14q24.3	10.13	10.27	0.53	0.52	-0.15	4.95E-03	0.0249	(B) TP53 wild in GC
CD180	5q12.3	6.49	6.92	1.53	1.55	-0.43	4.97E-03	0.0249	(B) TP53 wild in GC
CLINT1	5q33.3	11.47	11.65	0.69	0.61	-0.18	4.97E-03	0.0249	(B) TP53 wild in GC
NFIB	9p23-p22.3	10.75	11.04	1.02	1.05	-0.29	5.00E-03	0.025	(B) TP53 wild in GC
RWDD2B	21q21.3	7.99	8.17	0.61	0.68	-0.18	5.02E-03	0.0251	(B) TP53 wild in GC
CPEB2	4p15.32	8.78	8.97	0.67	0.71	-0.19	5.03E-03	0.0251	(B) TP53 wild in GC
RCN1	11p13	11.22	11.45	0.83	0.79	-0.23	5.05E-03	0.0252	(B) TP53 wild in GC
BORCS7	10q24.32	8.71	8.87	0.59	0.55	-0.16	5.06E-03	0.0253	(B) TP53 wild in GC
IKZF2	2q34	7.27	7.6	1.2	1.16	-0.33	5.08E-03	0.0253	(B) TP53 wild in GC
PLGRKT	9p24.1	7.95	8.17	0.76	0.83	-0.22	5.08E-03	0.0253	(B) TP53 wild in GC
LTA	6p21.33	3.63	4.07	1.55	1.57	-0.44	5.09E-03	0.0254	(B) TP53 wild in GC
WDR11	10q26.12	9.95	10.1	0.56	0.52	-0.15	5.12E-03	0.0255	(B) TP53 wild in GC
HMGN5	Xq21.1	6.94	7.35	1.55	1.43	-0.42	5.16E-03	0.0256	(B) TP53 wild in GC
RPS6KB1	17q23.1	9.41	9.54	0.47	0.45	-0.13	5.19E-03	0.0257	(B) TP53 wild in GC
PIK3C3	18q12.3	8.64	8.8	0.61	0.5	-0.16	5.19E-03	0.0257	(B) TP53 wild in GC
METTL7A	12q13.12	9.7	10.04	1.16	1.29	-0.34	5.22E-03	0.0258	(B) TP53 wild in GC

TPM1	15q22.2	12.14	12.43	0.95	1.17	-0.29	5.26E-03	0.026	(B) TP53 wild in GC
DMAC1	9p24.1	9.15	9.34	0.69	0.64	-0.19	5.26E-03	0.026	(B) TP53 wild in GC
LRP10	14q11.2	12.64	12.84	0.73	0.68	-0.2	5.26E-03	0.026	(B) TP53 wild in GC
KIAA0825	5q15	4.75	5	0.84	0.92	-0.24	5.28E-03	0.026	(B) TP53 wild in GC
PTPRM	18p11.23	9.15	9.42	0.88	1.1	-0.27	5.28E-03	0.026	(B) TP53 wild in GC
MCCC2	5q13.2	11.09	11.25	0.57	0.57	-0.16	5.28E-03	0.026	(B) TP53 wild in GC
CD52	1p36.11	8.21	8.63	1.39	1.64	-0.42	5.30E-03	0.0261	(B) TP53 wild in GC
ZNF540	19q13.12	4.22	4.56	1.19	1.26	-0.34	5.30E-03	0.0261	(B) TP53 wild in GC
HNRNPA1L	13q14.3	10.47	10.62	0.55	0.51	-0.15	5.31E-03	0.0261	(B) TP53 wild in GC
SMAD4	18q21.2	10.23	10.42	0.73	0.66	-0.19	5.34E-03	0.0262	(B) TP53 wild in GC
MXD4	4p16.3	10.19	10.37	0.64	0.66	-0.18	5.38E-03	0.0264	(B) TP53 wild in GC
CARF	2q33.2	6.67	6.9	0.83	0.87	-0.24	5.40E-03	0.0265	(B) TP53 wild in GC
ZNF425	7q36.1	5.06	5.34	1.03	0.93	-0.27	5.46E-03	0.0267	(B) TP53 wild in GC
C3ORF62	3p21.31	6.73	6.92	0.72	0.65	-0.19	5.46E-03	0.0267	(B) TP53 wild in GC
GPC4	Xq26.2	9.31	9.62	1.16	1.12	-0.32	5.50E-03	0.0269	(B) TP53 wild in GC
DDIT4	10q22.1	10.6	10.88	1	1.03	-0.28	5.52E-03	0.027	(B) TP53 wild in GC

FEZ2	2p22.2	9.14	9.29	0.5	0.63	-0.15	5.52E-03	0.027	(B) TP53 wild in GC
RNF38	9p13.2	9.97	10.14	0.62	0.65	-0.18	5.53E-03	0.027	(B) TP53 wild in GC
YWHAH	22q12.3	11.89	12.02	0.49	0.46	-0.13	5.53E-03	0.027	(B) TP53 wild in GC
PPP3CC	8p21.3	7.66	7.87	0.83	0.73	-0.22	5.57E-03	0.0271	(B) TP53 wild in GC
MS4A4A	11q12.2	7.13	7.53	1.45	1.41	-0.4	5.57E-03	0.0271	(B) TP53 wild in GC
TRIM13	13q14.2	9.45	9.59	0.54	0.47	-0.14	5.59E-03	0.0272	(B) TP53 wild in GC
TPT1	13q14.13	15.2	15.37	0.63	0.6	-0.17	5.63E-03	0.0273	(B) TP53 wild in GC
KITLG	12q21.32	10.01	10.29	1.01	1.05	-0.28	5.66E-03	0.0274	(B) TP53 wild in GC
HLA-DQA1	6p21.32	10.07	10.57	1.89	1.71	-0.5	5.67E-03	0.0275	(B) TP53 wild in GC
SEPTIN1	16p11.2	7.14	7.49	1.2	1.32	-0.35	5.69E-03	0.0275	(B) TP53 wild in GC
EIF3F	11p15.4	10.86	10.99	0.45	0.46	-0.12	5.69E-03	0.0275	(B) TP53 wild in GC
RSBN1	1p13.2	9.24	9.36	0.44	0.44	-0.12	5.70E-03	0.0276	(B) TP53 wild in GC
SNX1	15q22.31	10.73	10.84	0.39	0.43	-0.11	5.70E-03	0.0276	(B) TP53 wild in GC
WDFY4	10q11.23	7.33	7.84	1.84	1.87	-0.51	5.73E-03	0.0277	(B) TP53 wild in GC
RASGRP4	19q13.2	4.92	5.28	1.23	1.36	-0.35	5.74E-03	0.0277	(B) TP53 wild in GC
ACADSB	10q26.13	8.94	9.15	0.74	0.77	-0.21	5.74E-03	0.0277	(B) TP53 wild in GC

BBS7	4q27	7.81	7.95	0.5	0.45	-0.13	5.79E-03	0.0279	(B) TP53 wild in GC
REST	4q12	9.21	9.34	0.49	0.47	-0.13	5.81E-03	0.028	(B) TP53 wild in GC
GPRC5A	12p13.1	12.29	12.73	1.67	1.52	-0.44	5.85E-03	0.0282	(B) TP53 wild in GC
ITGA1	5q11.2	10.24	10.53	1.01	1.09	-0.29	5.87E-03	0.0282	(B) TP53 wild in GC
IGSF6	16p12.2	6.91	7.26	1.26	1.35	-0.36	5.87E-03	0.0282	(B) TP53 wild in GC
LY86	6p25.1	6.27	6.65	1.3	1.49	-0.38	5.92E-03	0.0284	(B) TP53 wild in GC
INTS6L	Xq26.3	6.84	7.13	1.1	1.01	-0.29	5.92E-03	0.0284	(B) TP53 wild in GC
DPT	1q24.2	5.68	6.3	2.16	2.4	-0.62	5.96E-03	0.0286	(B) TP53 wild in GC
GNS	12q14.3	11.98	12.14	0.59	0.64	-0.17	5.96E-03	0.0286	(B) TP53 wild in GC
AFF3	2q11.2	5.15	5.72	1.95	2.19	-0.56	5.96E-03	0.0286	(B) TP53 wild in GC
SUCLG2	3p14.1	10.17	10.33	0.64	0.56	-0.17	6.02E-03	0.0287	(B) TP53 wild in GC
RFX3	9p24.2	5.84	6.13	1.06	1	-0.28	6.02E-03	0.0288	(B) TP53 wild in GC
XAF1	17p13.1	9.99	10.31	1.21	1.13	-0.32	6.04E-03	0.0288	(B) TP53 wild in GC
ATP8A1	4p13	8.79	9.17	1.49	1.26	-0.38	6.12E-03	0.0291	(B) TP53 wild in GC
CXXC1	18q21.1	9.26	9.39	0.49	0.49	-0.13	6.13E-03	0.0292	(B) TP53 wild in GC
IPO8	12p11.21	10.33	10.47	0.53	0.5	-0.14	6.14E-03	0.0292	(B) TP53 wild in GC

SMAP2	1p34.2	9.92	10.11	0.69	0.7	-0.19	6.14E-03	0.0292	(B) TP53 wild in GC
TSTD2	9q22.33	8.65	8.79	0.52	0.52	-0.14	6.14E-03	0.0292	(B) TP53 wild in GC
PABPC4L	4q28.3	4.54	4.88	1.11	1.41	-0.34	6.17E-03	0.0293	(B) TP53 wild in GC
FBXL3	13q22.3	10.16	10.29	0.46	0.45	-0.13	6.18E-03	0.0293	(B) TP53 wild in GC
DZIP3	3q13.13	8.02	8.23	0.75	0.77	-0.21	6.18E-03	0.0293	(B) TP53 wild in GC
N4BP1	16q12.1	10.29	10.41	0.44	0.41	-0.12	6.20E-03	0.0294	(B) TP53 wild in GC
ARSK	5q15	6.8	6.95	0.59	0.52	-0.15	6.21E-03	0.0294	(B) TP53 wild in GC
RTRAF	14q22.1	10.82	10.97	0.56	0.48	-0.14	6.22E-03	0.0295	(B) TP53 wild in GC
TM2D2	8p11.22	9.4	9.55	0.58	0.51	-0.15	6.26E-03	0.0296	(B) TP53 wild in GC
SH2B3	12q24.12	10.13	10.31	0.66	0.66	-0.18	6.29E-03	0.0297	(B) TP53 wild in GC
SRA1	5q31.3	9.12	9.27	0.55	0.51	-0.14	6.30E-03	0.0297	(B) TP53 wild in GC
SUFU	10q24.32	8.81	8.95	0.53	0.52	-0.14	6.30E-03	0.0297	(B) TP53 wild in GC
FNDC3A	13q14.2	11.05	11.22	0.66	0.56	-0.17	6.30E-03	0.0297	(B) TP53 wild in GC
SYNE2	14q23.2	11.73	11.96	0.79	0.96	-0.24	6.30E-03	0.0297	(B) TP53 wild in GC
ZNF490	19p13.2-p1	7.69	7.85	0.57	0.54	-0.15	6.37E-03	0.03	(B) TP53 wild in GC
CD163L1	12p13.31	5.72	6.18	1.72	1.61	-0.46	6.38E-03	0.03	(B) TP53 wild in GC

ABCA1	9q31.1	9.72	9.98	0.96	1.02	-0.27	6.38E-03	0.03	(B) TP53 wild in GC
DHRS7B	17p11.2	8.39	8.56	0.67	0.57	-0.17	6.39E-03	0.0301	(B) TP53 wild in GC
DAPP1	4q23	6.96	7.35	1.43	1.41	-0.39	6.40E-03	0.0301	(B) TP53 wild in GC
NBAS	2p24.3	9.83	9.97	0.52	0.55	-0.14	6.41E-03	0.0301	(B) TP53 wild in GC
FZD4	11q14.2	9.17	9.41	0.85	0.93	-0.24	6.41E-03	0.0301	(B) TP53 wild in GC
SIGLEC10	19q13.41	7.37	7.77	1.47	1.47	-0.4	6.42E-03	0.0301	(B) TP53 wild in GC
ENC1	5q13.3	11.24	11.49	0.88	1	-0.25	6.42E-03	0.0301	(B) TP53 wild in GC
ZNF564	19p13.2	7.71	7.84	0.51	0.42	-0.13	6.42E-03	0.0301	(B) TP53 wild in GC
SLAMF7	1q23.3	8.28	8.72	1.54	1.65	-0.43	6.46E-03	0.0303	(B) TP53 wild in GC
SIRT1	10q21.3	9.04	9.18	0.54	0.49	-0.14	6.47E-03	0.0303	(B) TP53 wild in GC
ZNF366	5q13.2|5q	5	5.35	1.2	1.36	-0.34	6.47E-03	0.0303	(B) TP53 wild in GC
ST3GAL5	2p11.2	6.83	7.1	0.99	0.99	-0.27	6.53E-03	0.0304	(B) TP53 wild in GC
TMEM245	9q31.3	10.98	11.16	0.58	0.79	-0.18	6.53E-03	0.0304	(B) TP53 wild in GC
MOB3C	1p33	8.37	8.51	0.57	0.49	-0.15	6.54E-03	0.0305	(B) TP53 wild in GC
BTAF1	10q23.32	9.74	9.92	0.64	0.69	-0.18	6.55E-03	0.0305	(B) TP53 wild in GC
ELAC1	18q21.2	6.37	6.56	0.73	0.67	-0.19	6.59E-03	0.0307	(B) TP53 wild in GC

NFIC	19p13.3	10.47	10.7	0.8	0.93	-0.23	6.60E-03	0.0307	(B) TP53 wild in GC
JMY	5q14.1	9.11	9.31	0.64	0.85	-0.2	6.62E-03	0.0308	(B) TP53 wild in GC
UGCG	9q31.3	8.4	8.61	0.75	0.86	-0.22	6.62E-03	0.0308	(B) TP53 wild in GC
MAP3K1	5q11.2	9.67	9.85	0.63	0.73	-0.18	6.63E-03	0.0308	(B) TP53 wild in GC
TEC	19q13.2	5.76	5.98	0.86	0.76	-0.22	6.64E-03	0.0308	(B) TP53 wild in GC
MEF2C	5q14.3	8.85	9.16	1.05	1.21	-0.3	6.66E-03	0.0309	(B) TP53 wild in GC
TRIM21	11p15.4	8.89	9.06	0.65	0.63	-0.17	6.66E-03	0.0309	(B) TP53 wild in GC
SGCD	5q33.2-q33	7.95	8.37	1.47	1.69	-0.43	6.66E-03	0.0309	(B) TP53 wild in GC
EPM2A	6q24.3	6.41	6.66	0.87	0.99	-0.25	6.68E-03	0.0309	(B) TP53 wild in GC
SETD7	4q31.1	10.77	10.99	0.8	0.84	-0.22	6.69E-03	0.031	(B) TP53 wild in GC
ZEB1	10p11.22	9.59	9.91	1.08	1.32	-0.32	6.70E-03	0.031	(B) TP53 wild in GC
C3	19p13.3	12.84	13.33	1.73	1.93	-0.49	6.73E-03	0.0311	(B) TP53 wild in GC
LMBRD1	6q13	10.02	10.15	0.48	0.48	-0.13	6.77E-03	0.0312	(B) TP53 wild in GC
ZBTB4	17p13.1	10.74	10.94	0.68	0.79	-0.2	6.77E-03	0.0312	(B) TP53 wild in GC
IRAK3	12q14.3	8.13	8.52	1.34	1.56	-0.39	6.78E-03	0.0313	(B) TP53 wild in GC
CD14	5q31.3	9.98	10.27	1.05	1.11	-0.29	6.79E-03	0.0313	(B) TP53 wild in GC

LRIG1	3p14.1	10.74	11.01	0.92	1.1	-0.27	6.81E-03	0.0314	(B) TP53 wild in GC
APP	21q21.3	13.78	13.94	0.61	0.51	-0.15	6.83E-03	0.0314	(B) TP53 wild in GC
POPDC2	3q13.33	5.16	5.63	1.57	1.92	-0.47	6.83E-03	0.0314	(B) TP53 wild in GC
XPA	9q22.33	7.8	7.94	0.53	0.53	-0.14	6.83E-03	0.0314	(B) TP53 wild in GC
CARD16	11q22.3	6.72	7.03	1.18	1.09	-0.31	6.85E-03	0.0315	(B) TP53 wild in GC
STK10	5q35.1	9.72	9.9	0.65	0.66	-0.18	6.86E-03	0.0315	(B) TP53 wild in GC
TOR1AIP1	1q25.2	10.45	10.6	0.47	0.6	-0.14	6.89E-03	0.0316	(B) TP53 wild in GC
ASB7	15q26.3	8.74	8.87	0.48	0.47	-0.13	6.89E-03	0.0316	(B) TP53 wild in GC
HECTD2	10q23.32	7.21	7.49	1	1.08	-0.28	6.95E-03	0.0318	(B) TP53 wild in GC
YPEL5	2p23.1	10.12	10.26	0.49	0.56	-0.14	6.96E-03	0.0318	(B) TP53 wild in GC
SLC35A4	5q31.3	11.27	11.39	0.43	0.41	-0.11	6.96E-03	0.0318	(B) TP53 wild in GC
TAB2	6q25.1	10.91	11.03	0.43	0.47	-0.12	6.97E-03	0.0319	(B) TP53 wild in GC
TWSG1	18p11.22	10.21	10.42	0.73	0.78	-0.2	6.99E-03	0.0319	(B) TP53 wild in GC
PRKD3	2p22.2	9.36	9.59	0.87	0.88	-0.24	7.06E-03	0.0322	(B) TP53 wild in GC
CXCL13	4q21.1	7.54	8.19	2.39	2.42	-0.65	7.08E-03	0.0323	(B) TP53 wild in GC
MORC4	Xq22.3	9.23	9.46	0.85	0.87	-0.23	7.09E-03	0.0323	(B) TP53 wild in GC

DOI: 10.26502/jcsct.50790107

LAG3	12p13.31	6.63	7.05	1.56	1.56	-0.42	7.13E-03	0.0324	(B) TP53 wild in GC
ABO	9q34.2	6.86	7.43	2.12	2.12	-0.57	7.15E-03	0.0324	(B) TP53 wild in GC
EIF4G2	11p15.4	14.17	14.27	0.37	0.38	-0.1	7.15E-03	0.0324	(B) TP53 wild in GC
UBA7	3p21.31	9.56	9.8	0.93	0.86	-0.24	7.15E-03	0.0324	(B) TP53 wild in GC
KDM5A	12p13.33	10.51	10.63	0.46	0.48	-0.13	7.19E-03	0.0326	(B) TP53 wild in GC
LOC90784	2p11.2	8.08	8.26	0.63	0.7	-0.18	7.23E-03	0.0327	(B) TP53 wild in GC
APOBEC3C	22q13.1	8.34	8.63	1.04	1.09	-0.28	7.23E-03	0.0327	(B) TP53 wild in GC
FBXO10	9p13.2	7.35	7.55	0.74	0.71	-0.19	7.24E-03	0.0327	(B) TP53 wild in GC
PCGF6	10q24.33	7.11	7.26	0.56	0.54	-0.15	7.24E-03	0.0327	(B) TP53 wild in GC
SIT1	9p13.3	5.56	5.98	1.53	1.59	-0.42	7.24E-03	0.0327	(B) TP53 wild in GC
ST6GALNA	17q25.1	9.03	9.63	2.5	1.92	-0.61	7.24E-03	0.0327	(B) TP53 wild in GC
PTGFRN	1p13.1	11.12	11.3	0.61	0.72	-0.18	7.32E-03	0.033	(B) TP53 wild in GC
C1QBP	17p13.2	10.97	11.16	0.7	0.71	-0.19	7.32E-03	0.033	(B) TP53 wild in GC
LRRK2	12q12	6.65	7.09	1.52	1.79	-0.44	7.33E-03	0.033	(B) TP53 wild in GC
NAALADL2	3q26.31	6.73	7.13	1.55	1.38	-0.4	7.35E-03	0.033	(B) TP53 wild in GC
COL6A1	21q22.3	12.78	13.06	1.01	1.15	-0.29	7.36E-03	0.0331	(B) TP53 wild in GC

ZNF518A	10q24.1	9.53	9.74	0.81	0.73	-0.21	7.37E-03	0.0331	(B) TP53 wild in GC
ANKRD22	10q23.31	8.82	9.2	1.41	1.43	-0.38	7.39E-03	0.0331	(B) TP53 wild in GC
MDFIC	7q31.1-q31	8.8	9.08	0.99	1.12	-0.28	7.40E-03	0.0332	(B) TP53 wild in GC
MSL3	Xp22.2	9.2	9.32	0.46	0.42	-0.12	7.41E-03	0.0332	(B) TP53 wild in GC
CRIM1	2p22.2	10.74	10.95	0.76	0.76	-0.2	7.44E-03	0.0333	(B) TP53 wild in GC
LAMA4	6q21	10.8	11.05	0.86	1.06	-0.25	7.46E-03	0.0333	(B) TP53 wild in GC
SH2D3C	9q34.11	8.06	8.31	0.89	0.96	-0.25	7.46E-03	0.0333	(B) TP53 wild in GC
CD81	11p15.5	12.61	12.78	0.68	0.63	-0.18	7.50E-03	0.0335	(B) TP53 wild in GC
C5ORF15	5q31.1	10.47	10.63	0.6	0.57	-0.16	7.51E-03	0.0335	(B) TP53 wild in GC
PLEKHO2	15q22.31	9.43	9.64	0.77	0.84	-0.21	7.54E-03	0.0336	(B) TP53 wild in GC
LTA4H	12q23.1	10.76	10.89	0.45	0.54	-0.13	7.54E-03	0.0336	(B) TP53 wild in GC
PPARGC1B	5q32	6.82	7.12	1.18	1.02	-0.3	7.55E-03	0.0337	(B) TP53 wild in GC
DENND6A	3p14.3	9.1	9.22	0.45	0.47	-0.12	7.56E-03	0.0337	(B) TP53 wild in GC
MYO5A	15q21.2	8.76	9.03	0.91	1.11	-0.27	7.59E-03	0.0338	(B) TP53 wild in GC
TTLL11	9q33.2	6.33	6.54	0.75	0.83	-0.21	7.59E-03	0.0338	(B) TP53 wild in GC
CNOT4	7q33	9.08	9.17	0.34	0.32	-0.09	7.59E-03	0.0338	(B) TP53 wild in GC

FLT3LG	19q13.33	6.56	6.82	0.94	1.02	-0.26	7.60E-03	0.0338	(B) TP53 wild in GC
ATG4C	1p31.3	7.36	7.48	0.45	0.44	-0.12	7.60E-03	0.0338	(B) TP53 wild in GC
GTF2H2C	5q13.2	8.12	8.29	0.64	0.65	-0.17	7.63E-03	0.0339	(B) TP53 wild in GC
ZNF484	9q22.31	6.82	6.98	0.63	0.62	-0.17	7.64E-03	0.0339	(B) TP53 wild in GC
BACE2	21q22.2-q2	11.44	11.72	1.05	1.02	-0.28	7.64E-03	0.0339	(B) TP53 wild in GC
IQGAP2	5q13.3	10.4	10.77	1.43	1.3	-0.37	7.65E-03	0.0339	(B) TP53 wild in GC
GYPC	2q14.3	8.21	8.54	1.14	1.38	-0.33	7.67E-03	0.034	(B) TP53 wild in GC
HDAC3	5q31.3	9.37	9.47	0.41	0.39	-0.11	7.68E-03	0.034	(B) TP53 wild in GC
FGD2	6p21.2	7.01	7.34	1.16	1.29	-0.32	7.69E-03	0.0341	(B) TP53 wild in GC
RPSAP58	19p12	12.75	12.91	0.62	0.58	-0.16	7.73E-03	0.0342	(B) TP53 wild in GC
CNRIP1	2p14	7.05	7.32	0.9	1.2	-0.27	7.74E-03	0.0342	(B) TP53 wild in GC
RABGAP1	9q33.2-q33	10.06	10.22	0.55	0.6	-0.15	7.76E-03	0.0343	(B) TP53 wild in GC
NMUR1	2q37.1	4.48	4.87	1.39	1.6	-0.39	7.77E-03	0.0343	(B) TP53 wild in GC
CFD	19p13.3	8.31	8.71	1.46	1.61	-0.4	7.78E-03	0.0343	(B) TP53 wild in GC
CORO1A	16p11.2	9.53	9.85	1.17	1.23	-0.32	7.78E-03	0.0343	(B) TP53 wild in GC
SENP5	3q29	9.37	9.5	0.48	0.47	-0.13	7.83E-03	0.0345	(B) TP53 wild in GC

ICE2	15q22.2	9.58	9.7	0.46	0.47	-0.12	7.85E-03	0.0346	(B) TP53 wild in GC
8-Mar	10q11.21-q	8.1	8.32	0.83	0.8	-0.22	7.91E-03	0.0348	(B) TP53 wild in GC
FCMR	1q32.1	7.38	7.76	1.34	1.57	-0.38	7.96E-03	0.035	(B) TP53 wild in GC
LAMB2	3p21.31	11.67	11.9	0.83	0.86	-0.22	7.99E-03	0.0351	(B) TP53 wild in GC
RC3H2	9q33.2	9.12	9.29	0.67	0.64	-0.17	8.01E-03	0.0352	(B) TP53 wild in GC
KIF2A	5q12.1	9.21	9.38	0.65	0.61	-0.17	8.01E-03	0.0352	(B) TP53 wild in GC
SRGAP3	3p25.3	6.4	6.71	1.21	1.16	-0.31	8.03E-03	0.0352	(B) TP53 wild in GC
TMX1	14q22.1	10.18	10.31	0.5	0.45	-0.13	8.09E-03	0.0354	(B) TP53 wild in GC
SPCS3	4q34.2	11.37	11.5	0.46	0.49	-0.13	8.09E-03	0.0354	(B) TP53 wild in GC
ISCA1	9q21.33	8.98	9.11	0.48	0.51	-0.13	8.09E-03	0.0354	(B) TP53 wild in GC
ZNF266	19p13.2	8.74	8.92	0.69	0.69	-0.18	8.11E-03	0.0355	(B) TP53 wild in GC
NACC2	9q34.3	7.24	7.48	0.85	0.97	-0.24	8.14E-03	0.0356	(B) TP53 wild in GC
MORC3	21q22.12	9.45	9.59	0.49	0.53	-0.13	8.16E-03	0.0357	(B) TP53 wild in GC
SRGN	10q22.1	10.58	10.89	1.11	1.25	-0.31	8.17E-03	0.0357	(B) TP53 wild in GC
ADAMTSL1	9p22.2-p22	6.21	6.6	1.39	1.61	-0.39	8.19E-03	0.0357	(B) TP53 wild in GC
MRNIP	5q35.3	5.94	6.14	0.74	0.78	-0.2	8.20E-03	0.0358	(B) TP53 wild in GC

DAAM1	14q23.1	8.91	9.08	0.61	0.67	-0.17	8.22E-03	0.0358	(B) TP53 wild in GC
FAM102B	1p13.3	10.01	10.2	0.75	0.69	-0.19	8.24E-03	0.0359	(B) TP53 wild in GC
ALCAM	3q13.11	9.79	10.09	1.12	1.16	-0.3	8.24E-03	0.0359	(B) TP53 wild in GC
PELO	5q11.2	8.89	9.01	0.45	0.47	-0.12	8.26E-03	0.036	(B) TP53 wild in GC
RIPK1	6p25.2	9.89	9.99	0.38	0.36	-0.1	8.28E-03	0.036	(B) TP53 wild in GC
USP46	4q12	8.38	8.52	0.58	0.51	-0.14	8.35E-03	0.0362	(B) TP53 wild in GC
DOCK4	7q31.1	8.14	8.38	0.88	0.95	-0.24	8.37E-03	0.0363	(B) TP53 wild in GC
DOCK5	8p21.2	9.66	9.9	0.93	0.91	-0.24	8.43E-03	0.0365	(B) TP53 wild in GC
ZNF20	19p13.2	6.49	6.64	0.57	0.53	-0.15	8.44E-03	0.0366	(B) TP53 wild in GC
LYRM7	5q23.3-q31	8.55	8.71	0.58	0.61	-0.16	8.46E-03	0.0366	(B) TP53 wild in GC
CCR7	17q21.2	6.11	6.59	1.7	1.98	-0.48	8.46E-03	0.0366	(B) TP53 wild in GC
TGFBR2	3p24.1	11.62	11.85	0.77	0.97	-0.23	8.48E-03	0.0366	(B) TP53 wild in GC
PHC3	3q26.2	10.75	10.91	0.63	0.57	-0.16	8.48E-03	0.0367	(B) TP53 wild in GC
ZNF565	19q13.12	6.14	6.29	0.62	0.53	-0.15	8.50E-03	0.0367	(B) TP53 wild in GC
DPY19L1	7p14.2	10.06	10.26	0.73	0.75	-0.19	8.51E-03	0.0367	(B) TP53 wild in GC
ARHGEF28	5q13.2	8.35	8.61	1.03	0.94	-0.26	8.55E-03	0.0369	(B) TP53 wild in GC

SMAD1	4q31.21	9.12	9.25	0.44	0.5	-0.12	8.56E-03	0.0369	(B) TP53 wild in GC
IFT27	22q12.3	7.82	8	0.72	0.67	-0.18	8.56E-03	0.0369	(B) TP53 wild in GC
RAB27B	18q21.2	7.15	7.61	1.87	1.64	-0.47	8.58E-03	0.037	(B) TP53 wild in GC
CAMK2D	4q26	10.06	10.22	0.62	0.6	-0.16	8.64E-03	0.0371	(B) TP53 wild in GC
JAK3	19p13.11	8.7	8.99	1.04	1.18	-0.29	8.66E-03	0.0372	(B) TP53 wild in GC
SSH1	12q24.11	10.32	10.46	0.51	0.56	-0.14	8.67E-03	0.0372	(B) TP53 wild in GC
DISC1	1q42.2	6.83	7.07	0.9	0.92	-0.24	8.69E-03	0.0373	(B) TP53 wild in GC
HAVCR2	5q33.3	7.66	7.98	1.21	1.22	-0.32	8.71E-03	0.0374	(B) TP53 wild in GC
ZNF234	19q13.31	6.98	7.17	0.83	0.64	-0.2	8.72E-03	0.0374	(B) TP53 wild in GC
LSP1	11p15.5	9.83	10.13	1.09	1.21	-0.3	8.73E-03	0.0374	(B) TP53 wild in GC
NLRP3	1q44	5.28	5.64	1.33	1.37	-0.35	8.79E-03	0.0376	(B) TP53 wild in GC
SLC24A1	15q22.31	8.16	8.32	0.61	0.59	-0.16	8.84E-03	0.0378	(B) TP53 wild in GC
MLLT3	9p21.3	8.21	8.47	0.98	0.93	-0.25	8.94E-03	0.0381	(B) TP53 wild in GC
HOMEZ	14q11.2	8.39	8.53	0.56	0.51	-0.14	8.95E-03	0.0381	(B) TP53 wild in GC
CXCR3	Xq13.1	6.55	6.91	1.31	1.52	-0.36	8.97E-03	0.0382	(B) TP53 wild in GC
CNN2	19p13.3	11.47	11.66	0.73	0.72	-0.19	8.97E-03	0.0382	(B) TP53 wild in GC

ADORA3	1p13.2	5.89	6.27	1.39	1.49	-0.37	9.01E-03	0.0384	(B) TP53 wild in GC
ITGAV	2q32.1	11.57	11.77	0.78	0.78	-0.2	9.04E-03	0.0385	(B) TP53 wild in GC
APBB2	4p14-p13	9.95	10.13	0.67	0.71	-0.18	9.05E-03	0.0385	(B) TP53 wild in GC
PDE4C	19p13.11	5.59	6.08	1.84	1.97	-0.49	9.05E-03	0.0385	(B) TP53 wild in GC
LINC02035	3q21.1	7.48	7.68	0.73	0.83	-0.2	9.07E-03	0.0386	(B) TP53 wild in GC
BNIP2	15q22.2	9.44	9.59	0.58	0.56	-0.15	9.09E-03	0.0386	(B) TP53 wild in GC
SGMS1	10q11.23	9.4	9.55	0.59	0.55	-0.15	9.09E-03	0.0386	(B) TP53 wild in GC
RAB33A	Xq26.1	3.51	3.8	1.13	1.12	-0.29	9.11E-03	0.0387	(B) TP53 wild in GC
TMEM173	5q31.2	8.89	9.13	0.86	0.94	-0.23	9.11E-03	0.0387	(B) TP53 wild in GC
TCF4	18q21.2	9.92	10.16	0.85	1.06	-0.25	9.12E-03	0.0387	(B) TP53 wild in GC
RPSA	3p22.1	11.06	11.22	0.62	0.6	-0.16	9.12E-03	0.0387	(B) TP53 wild in GC
ETFBKMT	12p11.21	4.98	5.17	0.76	0.72	-0.19	9.16E-03	0.0388	(B) TP53 wild in GC
METTL8	2q31.1	8.63	8.79	0.63	0.55	-0.16	9.17E-03	0.0389	(B) TP53 wild in GC
AFAP1	4p16.1	10.49	10.68	0.69	0.78	-0.19	9.17E-03	0.0389	(B) TP53 wild in GC
ELL2	5q15	9.46	9.67	0.8	0.85	-0.21	9.21E-03	0.039	(B) TP53 wild in GC
HS3ST1	4p15.33	8.54	8.86	1.19	1.27	-0.32	9.22E-03	0.039	(B) TP53 wild in GC

HDAC4	2q37.3	9.11	9.34	0.84	0.88	-0.22	9.23E-03	0.039	(B) TP53 wild in GC
IFIT5	10q23.31	8.81	8.99	0.67	0.72	-0.18	9.26E-03	0.0391	(B) TP53 wild in GC
SLC25A28	10q24.2	8.87	9.01	0.51	0.56	-0.14	9.28E-03	0.0392	(B) TP53 wild in GC
ATF7	12q13.13	10.79	10.91	0.4	0.46	-0.11	9.29E-03	0.0392	(B) TP53 wild in GC
CUL1	7q36.1	10.37	10.48	0.45	0.38	-0.11	9.32E-03	0.0393	(B) TP53 wild in GC
ADGRE2	19p13.12	7.43	7.73	1.07	1.26	-0.3	9.37E-03	0.0395	(B) TP53 wild in GC
ZNF275	Xq28	9.47	9.62	0.58	0.51	-0.14	9.38E-03	0.0395	(B) TP53 wild in GC
INKA2	1p13.2	5.2	5.5	1.15	1.19	-0.3	9.46E-03	0.0398	(B) TP53 wild in GC
GRHPR	9p13.2	10	10.14	0.53	0.52	-0.14	9.48E-03	0.0398	(B) TP53 wild in GC
EPHA3	3p11.1	7.2	7.64	1.59	1.8	-0.44	9.50E-03	0.0399	(B) TP53 wild in GC
SEPTIN11	4q21.1	11.35	11.5	0.59	0.59	-0.15	9.58E-03	0.0402	(B) TP53 wild in GC
YLPM1	14q24.3	10.48	10.58	0.4	0.4	-0.1	9.59E-03	0.0402	(B) TP53 wild in GC
TLL1	4q32.3	4.1	4.54	1.45	1.98	-0.44	9.62E-03	0.0403	(B) TP53 wild in GC
UBE2J1	6q15	11.18	11.3	0.43	0.45	-0.11	9.63E-03	0.0404	(B) TP53 wild in GC
PYGM	11q13.1	4.04	4.59	2.02	2.27	-0.55	9.63E-03	0.0404	(B) TP53 wild in GC
NR3C1	5q31.3	9.57	9.83	0.97	1.07	-0.26	9.66E-03	0.0404	(B) TP53 wild in GC

DDX42	17q23.3	11.02	11.1	0.34	0.33	-0.09	9.68E-03	0.0405	(B) TP53 wild in GC
FBXO22	15q24.2	8.59	8.7	0.46	0.46	-0.12	9.68E-03	0.0405	(B) TP53 wild in GC
INPP5B	1p34.3	8.5	8.65	0.58	0.6	-0.15	9.69E-03	0.0405	(B) TP53 wild in GC
KYAT3	1p22.2	8.77	8.93	0.56	0.64	-0.15	9.71E-03	0.0406	(B) TP53 wild in GC
KLHDC1	14q21.3	5.23	5.51	0.96	1.2	-0.28	9.72E-03	0.0406	(B) TP53 wild in GC
TCEAL7	Xq22.2	4.23	4.6	1.31	1.61	-0.37	9.75E-03	0.0407	(B) TP53 wild in GC
ZNF800	7q31.33	9.26	9.38	0.5	0.44	-0.12	9.76E-03	0.0407	(B) TP53 wild in GC
ZNF445	3p21.31	9.12	9.26	0.55	0.53	-0.14	9.78E-03	0.0407	(B) TP53 wild in GC
ZFP30	19q13.12	6.48	6.82	1.38	1.24	-0.34	9.79E-03	0.0407	(B) TP53 wild in GC
CREB3L2	7q33	11.01	11.2	0.72	0.72	-0.19	9.81E-03	0.0408	(B) TP53 wild in GC
NUDT13	10q22.2	5.14	5.4	1.09	0.87	-0.26	9.82E-03	0.0408	(B) TP53 wild in GC
CYP4X1	1p33|1	5.87	6.41	2.06	2.17	-0.54	9.84E-03	0.0409	(B) TP53 wild in GC
SMYD4	17p13.3	8.03	8.15	0.49	0.45	-0.12	9.88E-03	0.041	(B) TP53 wild in GC
PECAM1	17q23.3	9.63	9.85	0.82	0.95	-0.23	9.88E-03	0.041	(B) TP53 wild in GC
MRO	18q21.2	3.61	3.99	1.54	1.37	-0.38	9.90E-03	0.0411	(B) TP53 wild in GC
PLCD4	2q35	5.68	5.92	0.81	1.08	-0.24	9.90E-03	0.0411	(B) TP53 wild in GC

ALDH1B1	9p13.1	10.39	10.66	0.99	1.15	-0.27	9.93E-03	0.0411	(B) TP53 wild in GC
DTX1	12q24.13	6.73	7.1	1.38	1.52	-0.37	9.94E-03	0.0412	(B) TP53 wild in GC
ABLIM1	10q25.3	11.82	12.05	0.93	0.82	-0.23	9.97E-03	0.0413	(B) TP53 wild in GC
HACD4	9p21.3	7.53	7.83	1.08	1.25	-0.3	9.98E-03	0.0413	(B) TP53 wild in GC
CHMP7	8p21.3	9.73	9.86	0.53	0.48	-0.13	9.99E-03	0.0413	(B) TP53 wild in GC
GALNT12	9q22.33	9.41	9.67	1.03	0.96	-0.26	9.99E-03	0.0413	(B) TP53 wild in GC
PCMTD1	8q11.23	9.98	10.14	0.67	0.63	-0.17	1.00E-02	0.0413	(B) TP53 wild in GC
TRAPPC13	5q12.3	8.38	8.5	0.48	0.42	-0.12	0.01	0.0413	(B) TP53 wild in GC
MPDZ	9p23	7.65	8.02	1.29	1.58	-0.36	0.01	0.0414	(B) TP53 wild in GC
LOXL1	15q24.1	9.37	9.65	1.09	1.13	-0.29	0.01	0.0414	(B) TP53 wild in GC
FAM13A-A	4q22.1	4.83	5.1	1.05	1.1	-0.27	0.0102	0.0418	(B) TP53 wild in GC
MIR22HG	17p13.3	6.95	7.18	0.93	0.85	-0.23	0.0102	0.042	(B) TP53 wild in GC
NCF1	7q11.23	6.9	7.28	1.4	1.53	-0.37	0.0102	0.042	(B) TP53 wild in GC
DNAJC18	5q31.2	6.33	6.57	0.88	0.99	-0.24	0.0102	0.0421	(B) TP53 wild in GC
UBP1	3p22.3	10.61	10.72	0.45	0.42	-0.11	0.0103	0.0421	(B) TP53 wild in GC
STYX	14q22.1	9.08	9.2	0.48	0.44	-0.12	0.0104	0.0427	(B) TP53 wild in GC

APOBEC3F	22q13.1	7.04	7.3	1.03	1.03	-0.26	0.0105	0.0428	(B) TP53 wild in GC
RBMXL1	1p22.2	9.52	9.63	0.42	0.43	-0.11	0.0105	0.0429	(B) TP53 wild in GC
ZNF501	3p21.31	5.75	5.99	0.93	0.94	-0.24	0.0105	0.043	(B) TP53 wild in GC
USP8	15q21.2	10.28	10.38	0.38	0.37	-0.1	0.0105	0.043	(B) TP53 wild in GC
MACF1	1p34.3	11.98	12.17	0.67	0.89	-0.2	0.0106	0.0431	(B) TP53 wild in GC
HLA-DRB1	6p21.32	11.17	11.57	1.6	1.44	-0.39	0.0106	0.0433	(B) TP53 wild in GC
RAI1	17p11.2	10.58	10.74	0.62	0.64	-0.16	0.0106	0.0433	(B) TP53 wild in GC
NDFIP1	5q31.3	11.33	11.44	0.43	0.4	-0.11	0.0106	0.0433	(B) TP53 wild in GC
ZNF510	9q22.33	8.08	8.22	0.57	0.53	-0.14	0.0107	0.0435	(B) TP53 wild in GC
GPR82	Xp11.4	4.04	4.36	1.17	1.43	-0.33	0.0107	0.0435	(B) TP53 wild in GC
CTDSPL	3p22.2	10.71	10.86	0.59	0.6	-0.15	0.0107	0.0436	(B) TP53 wild in GC
ADCK2	7q34	9.03	9.2	0.67	0.59	-0.16	0.0108	0.0438	(B) TP53 wild in GC
VPS39	15q15.1	10.45	10.55	0.37	0.42	-0.1	0.0108	0.044	(B) TP53 wild in GC
MZF1-AS1	19q13.43	4.25	4.48	0.97	0.83	-0.23	0.0109	0.044	(B) TP53 wild in GC

SCAF11	12q12	11.33	11.45	0.48	0.42	-0.12	0.0109	0.044	(B) TP53 wild in GC
FPR3	19q13.41	8.36	8.72	1.37	1.44	-0.35	0.011	0.0446	(B) TP53 wild in GC
LEPROTL1	8p12	9.97	10.11	0.53	0.52	-0.13	0.0111	0.0446	(B) TP53 wild in GC
VAV1	19p13.3	7.18	7.51	1.31	1.25	-0.33	0.0111	0.0446	(B) TP53 wild in GC
ACAP2	3q29	10.57	10.69	0.48	0.51	-0.13	0.0111	0.0447	(B) TP53 wild in GC
GMPR2	14q12	9.26	9.37	0.43	0.39	-0.11	0.0111	0.0447	(B) TP53 wild in GC
SELL	1q24.2	7.77	8.16	1.45	1.66	-0.39	0.0112	0.045	(B) TP53 wild in GC
PDS5A	4p14	11.54	11.65	0.45	0.44	-0.11	0.0112	0.045	(B) TP53 wild in GC
L3HYPDH	14q23.1	6.17	6.33	0.6	0.67	-0.16	0.0112	0.045	(B) TP53 wild in GC
TRAPPC2	Xp22.2	7.74	7.89	0.63	0.57	-0.15	0.0113	0.0452	(B) TP53 wild in GC
LRRC8C	1p22.2	8.74	8.94	0.77	0.83	-0.2	0.0113	0.0454	(B) TP53 wild in GC
SIMC1	5q35.2	8.68	8.88	0.78	0.85	-0.2	0.0113	0.0455	(B) TP53 wild in GC
C1QC	1p36.12	11.06	11.4	1.35	1.35	-0.34	0.0113	0.0455	(B) TP53 wild in GC
SQOR	15q21.1	10.33	10.53	0.84	0.76	-0.2	0.0113	0.0455	(B) TP53 wild in GC
STAG1	3q22.3	9.65	9.8	0.57	0.56	-0.14	0.0114	0.0457	(B) TP53 wild in GC

MGC16275	17q25.1	4.59	4.78	0.79	0.71	-0.19	0.0114	0.0458	(B) TP53 wild in GC
P2RY11	19p13.2	7.29	7.53	0.95	0.91	-0.24	0.0115	0.0459	(B) TP53 wild in GC
CBLB	3q13.11	9.31	9.45	0.57	0.56	-0.14	0.0115	0.0459	(B) TP53 wild in GC
DCLK2	4q31.23-q3	5.48	5.85	1.32	1.63	-0.37	0.0115	0.0459	(B) TP53 wild in GC
CEP170	1q43	9.51	9.71	0.76	0.77	-0.19	0.0115	0.0461	(B) TP53 wild in GC
ARHGAP22	10q11.22-q	5.14	5.44	1.13	1.26	-0.3	0.0115	0.0461	(B) TP53 wild in GC
RPS15	19p13.3	12.21	12.4	0.8	0.74	-0.19	0.0115	0.0461	(B) TP53 wild in GC
ERMAP	1p34.2	7.75	7.9	0.6	0.62	-0.15	0.0115	0.0461	(B) TP53 wild in GC
MNDA	1q23.1	7	7.33	1.22	1.43	-0.33	0.0116	0.0462	(B) TP53 wild in GC
MAFB	20q12	9.34	9.63	1.09	1.2	-0.29	0.0116	0.0463	(B) TP53 wild in GC
PHF7	3p21.1	5.48	5.67	0.77	0.75	-0.19	0.0116	0.0465	(B) TP53 wild in GC
SAMM50	22q13.31	9.7	9.81	0.47	0.42	-0.11	0.0117	0.0465	(B) TP53 wild in GC
ZCCHC10	5q31.1	8.22	8.33	0.48	0.45	-0.12	0.0117	0.0465	(B) TP53 wild in GC
PLEK	2p14	8.4	8.76	1.36	1.46	-0.35	0.0117	0.0467	(B) TP53 wild in GC
SH3RF2	5q32	9.18	9.57	1.79	1.21	-0.39	0.0117	0.0467	(B) TP53 wild in GC
TANK	2q24.2	9.77	9.89	0.5	0.43	-0.12	0.0118	0.0468	(B) TP53 wild in GC

PRRT1	6p21.32	5.5	5.82	1.21	1.32	-0.32	0.0118	0.047	(B) TP53 wild in GC
HAUS4	14q11.2	8.79	8.92	0.54	0.53	-0.13	0.0119	0.0471	(B) TP53 wild in GC
CIPC	14q24.3	9.17	9.3	0.45	0.56	-0.13	0.0119	0.0471	(B) TP53 wild in GC
MRAS	3q22.3	8.1	8.38	1.05	1.21	-0.28	0.0119	0.0471	(B) TP53 wild in GC
CITED2	6q24.1	9.64	9.84	0.76	0.86	-0.2	0.0119	0.0471	(B) TP53 wild in GC
PIP4K2A	10p12.2	9.54	9.71	0.64	0.68	-0.16	0.012	0.0474	(B) TP53 wild in GC
AOPEP	9q22.32	8.98	9.18	0.74	0.81	-0.19	0.012	0.0474	(B) TP53 wild in GC
GOLGB1	3q13.33	11.78	11.93	0.56	0.61	-0.15	0.012	0.0476	(B) TP53 wild in GC
STX12	1p35.3	10.05	10.15	0.41	0.42	-0.1	0.012	0.0477	(B) TP53 wild in GC
RRAGA	9p22.1	10.19	10.31	0.5	0.49	-0.12	0.012	0.0477	(B) TP53 wild in GC
MSN	Xq12	12.4	12.62	0.81	0.95	-0.22	0.0121	0.0478	(B) TP53 wild in GC
PTPRB	12q15	9.45	9.67	0.87	0.89	-0.22	0.0122	0.0483	(B) TP53 wild in GC
SLC25A53	Xq22.2	3.87	4.14	1.08	1.12	-0.27	0.0124	0.0486	(B) TP53 wild in GC
GPR68	14q32.11	7.27	7.54	1.04	1.11	-0.27	0.0124	0.0487	(B) TP53 wild in GC
UBASH3B	11q24.1	7.77	8.04	1.14	1.04	-0.27	0.0124	0.0487	(B) TP53 wild in GC
FGD4	12p11.21	9.75	9.96	0.88	0.76	-0.21	0.0124	0.0489	(B) TP53 wild in GC

EFR3A	8q24.22	11.07	11.18	0.46	0.47	-0.12	0.0124	0.0489	(B) TP53 wild in GC
CALCRL	2q32.1	8.99	9.24	0.88	1.13	-0.25	0.0125	0.049	(B) TP53 wild in GC
ZSWIM6	5q12.1	8.9	9.04	0.56	0.56	-0.14	0.0125	0.0491	(B) TP53 wild in GC
ARID2	12q12	10.02	10.16	0.57	0.51	-0.14	0.0125	0.0491	(B) TP53 wild in GC
SETD9	5q11.2	6.28	6.51	0.93	0.96	-0.24	0.0125	0.0491	(B) TP53 wild in GC
ANKRD17	4q13.3	11.45	11.55	0.42	0.41	-0.1	0.0125	0.0492	(B) TP53 wild in GC
FRMD6	14q22.1	8.48	8.79	1.19	1.37	-0.31	0.0126	0.0495	(B) TP53 wild in GC
NCK1	3q22.3	9.13	9.26	0.52	0.48	-0.12	0.0128	0.05	(B) TP53 wild in GC

Table S1: 1363 mRNA highly expressed in TP53 wild-type gastric cancer.

This article is an open access article distributed under the terms and conditions of the
Creative Commons Attribution (CC-BY) license 4.0
